# Supplementary material for: Comparison of chloroplast genomes and phylogenomics in the Ficus sarmentosa complex (Moraceae)
Source: PLoS One. 2022 Dec 30;17(12):e0279849. doi: 10.1371/journal.pone.0279849 (PMC9803296; doi:10.1371/journal.pone.0279849)
Supplement: Attachment — Submitted filename: PONE-D-22-13870_R2_edit.pdf [file pone.0279849.s005.pdf]

PLOS ONE

Comparison of chloroplast genomes and phylogenomics in the Ficus sarmentosa complex (Moraceae)

--Manuscript Draft--

**Manuscript Number:**	PONE-D-22-13870R2
**Article Type:**	Research Article
**Full Title:**	Comparison of chloroplast genomes and phylogenomics in the Ficus sarmentosa complex (Moraceae)
**Short Title:**	Comparative chloroplast genomics of the Ficus sarmetosa complex
**Corresponding Author:**	Chi-Yuan Yao<br>Tongji University<br>Shanghai, --请选择-- CHINA
**Keywords:**	Ficus; plastome; Comparative genomics; Phylogenomics; high-variable regions
**Abstract:**	Due to maternal inheritance and minimal rearrangement, the chloroplast genome is an important genetic resource for evolutionary studies. However, the evolutionary dynamics and phylogenetic performance of chloroplast genomes in closely related species are poorly characterized, particularly in taxonomically complex and species-rich group. The taxonomically unresolved Ficussarmentosa species complex (Moraceae) comprises approximately 20 taxa with genetic background unclear. In this study, we explored the evolutionary dynamics, hotspot loci, and phylogenetic performance of thirteen chloroplast genomes (including eleven newly obtained and two downloaded from NCBI) representing the F. sarmentosa complex. Their sequence lengths, IR boundaries, repeat sequences, and codon usage were compared. Both sequence length and IR boundaries were found to be highly conserved. All four categories of long repeat sequences were found across all 13 chloroplast genomes, with palindromic and forward sequences being the most common. The number of simple sequence repeat (SSR) loci varied from 175 (F. dinganensis and F. howii) to 190 (F. polynervis), with the dinucleotide motif appearing the most frequently. Relative synonymous codon usage (RSCU) analysis indicated that codons ending with A/T were prior to those ending with C/T. The majority of coding sequence regions were found to have undergone negative selection with the exception of ten genes (accD, clpP, ndhK, rbcL, rpl20, rpl22, rpl23, rpoC1, rps15, and rps4) which exhibited potential positive selective signatures. Five hypervariable genic regions (rps15, ycf1, rpoA, ndhF, and rpl22) and five hypervariable intergenic regions (trnH-GUG-psbA, rpl32-trnL-UAG, psbZ-trnG-GCC, trnK-UUU-rps16 and ndhF-rpl32) were identified. Overall, phylogenomic analysis based on 123 Ficus chloroplast genomes showed promise for studying the evolutionary relationships in Ficus, despite cyto-nuclear discordance. Furthermore, based on the phylogenetic performance of the F. sarmentosa complex and F. auriculata complex, the chloroplast genome also exhibited a promising phylogenetic resolution in closely related species.
**Order of Authors:**	Zhen Zhang
	De-Shun Zhang
	Lu Zou
	Chi-Yuan Yao
**Opposed Reviewers:**	
**Response to Reviewers:**	None.
**Additional Information:**	
**Question**	**Response**
**Financial Disclosure**<br><br>Enter a financial disclosure statement that	This research was funded by National Natural Science Foundation of China from Dr. De-Shun Zhang (grant number 32071824). The funder De-Shun Zhang implemented the conceptualization, visualization, and draft review of the study.

Powered by Editorial Manager® and ProduXion Manager® from Aries Systems Corporation

describes the sources of funding for the work included in this submission. Review the submission guidelines for detailed requirements. View published research articles from *PLOS ONE* for specific examples.<br><br>This statement is required for submission and **will appear in the published article** if the submission is accepted. Please make sure it is accurate.<br><br>**Unfunded studies**<br>Enter: *The author(s) received no specific funding for this work.*<br><br>**Funded studies**<br>Enter a statement with the following details:<br>• Initials of the authors who received each award<br>• Grant numbers awarded to each author<br>• The full name of each funder<br>• URL of each funder website<br>• Did the sponsors or funders play any role in the study design, data collection and analysis, decision to publish, or preparation of the manuscript?<br>• **NO** - Include this sentence at the end of your statement: *The funders had no role in study design, data collection and analysis, decision to publish, or preparation of the manuscript.*<br>• **YES** - Specify the role(s) played.<br><br>* typeset	
**Competing Interests**<br><br>Use the instructions below to enter a competing interest statement for this submission. On behalf of all authors, disclose any competing interests that could be perceived to bias this work—acknowledging all financial support and any other relevant financial or non-financial competing interests.<br><br>This statement is required for submission and **will appear in the published article** if the submission is accepted. Please make sure it is accurate and that any funding	The authors have declared that no competing interests exist.

sources listed in your Funding Information later in the submission form are also declared in your Financial Disclosure statement.<br><br>View published research articles from *PLOS ONE* for specific examples.<br><br>**NO authors have competing interests**<br><br>Enter: *The authors have declared that no competing interests exist.*<br><br>**Authors with competing interests**<br><br>Enter competing interest details beginning with this statement:<br><br>*I have read the journal's policy and the authors of this manuscript have the following competing interests: [insert competing interests here]*<br><br>* typeset	
**Ethics Statement**<br><br>Enter an ethics statement for this submission. This statement is required if the study involved:<br><br>• Human participants<br>• Human specimens or tissue<br>• Vertebrate animals or cephalopods<br>• Vertebrate embryos or tissues<br>• Field research<br><br>Write "N/A" if the submission does not require an ethics statement.<br><br>General guidance is provided below. Consult the submission guidelines for detailed instructions. **Make sure that all information entered here is included in the Methods section of the manuscript.**	N/A

**Format for specific study types**<br><br>**Human Subject Research (involving human participants and/or tissue)**<br>• Give the name of the institutional review board or ethics committee that approved the study<br>• Include the approval number and/or a statement indicating approval of this research<br>• Indicate the form of consent obtained (written/oral) or the reason that consent was not obtained (e.g. the data were analyzed anonymously)<br><br>**Animal Research (involving vertebrate animals, embryos or tissues)**<br>• Provide the name of the Institutional Animal Care and Use Committee (IACUC) or other relevant ethics board that reviewed the study protocol, and indicate whether they approved this research or granted a formal waiver of ethical approval<br>• Include an approval number if one was obtained<br>• If the study involved *non-human primates*, add *additional details* about animal welfare and steps taken to ameliorate suffering<br>• If anesthesia, euthanasia, or any kind of animal sacrifice is part of the study, include briefly which substances and/or methods were applied<br><br>**Field Research**<br><br>Include the following details if this study involves the collection of plant, animal, or other materials from a natural setting:<br>• Field permit number<br>• Name of the institution or relevant body that granted permission	
**Data Availability**<br><br>Authors are required to make all data underlying the findings described fully available, without restriction, and from the time of publication. PLOS allows rare exceptions to address legal and ethical concerns. See the PLOS Data Policy and FAQ for detailed information.	Yes - all data are fully available without restriction

Powered by Editorial Manager® and ProduXion Manager® from Aries Systems Corporation

A Data Availability Statement describing where the data can be found is required at submission. Your answers to this question constitute the Data Availability Statement and **will be published in the article**, if accepted.<br><br>**Important:** Stating 'data available on request from the author' is not sufficient. If your data are only available upon request, select 'No' for the first question and explain your exceptional situation in the text box.<br><br>Do the authors confirm that all data underlying the findings described in their manuscript are fully available without restriction?	
**Describe where the data may be found in full sentences. If you are copying our sample text, replace any instances of XXX with the appropriate details.**<br><br>• If the data are **held or will be held in a public repository**, include URLs, accession numbers or DOIs. If this information will only be available after acceptance, indicate this by ticking the box below. For example: *All XXX files are available from the XXX database (accession number(s) XXX, XXX.)*.<br>• If the data are all contained **within the manuscript and/or Supporting Information files**, enter the following: *All relevant data are within the manuscript and its Supporting Information files.*<br>• If neither of these applies but you are able to provide **details of access elsewhere**, with or without limitations, please do so. For example:<br><br>*Data cannot be shared publicly because of [XXX]. Data are available from the XXX Institutional Data Access / Ethics Committee (contact via XXX) for researchers who meet the criteria for access to confidential data.*<br><br>*The data underlying the results presented in the study are available from (include the name of the third party*	The eleven newly obtained complete chloroplast genome sequences that support the findings in the study are deposited in the NCBI with accession numbers as followed: ON257105-ON257115. The corresponding high throughput Illumina sequencing reads are deposited to Genome Sequence Archive (GSA) in BIG Data Center (https://bigd.big.ac.cn/gsa/) under BioProject accession: PRJCA012975.

and contact information or URL).<br>• This text is appropriate if the data are owned by a third party and authors do not have permission to share the data.<br><br>* typeset	
Additional data availability information:	Tick here if the URLs/accession numbers/DOIs will be available only after acceptance of the manuscript for publication so that we can ensure their inclusion before publication.

Powered by Editorial Manager® and ProduXion Manager® from Aries Systems Corporation

Dear Editor,

We sought a professional editing service to improve our language. All the changes were tracked, so another clean version was provided meanwhile. We hope that the revised manuscript could meet the language requirement of PLoS One.

We look forward to hearing from you at your earliest convenience.

Sincerely,

Chi-Yuan Yao
College of Architecture and Urban Planning, Tongji University, Shanghai, China
cyyao@tongji.edu.cn

Manuscript

Click here to access/download;Manuscript;manuscript-clean version.docx

Comparison of chloroplast genomes and phylogenomics in the *Ficus sarmentosa* complex (Moraceae)

Zhen Zhang[1], De-Shun Zhang[1], Zou Lu[2], Chi-Yuan Yao[*1]

[1] College of Architecture and Urban Planning, Tongji University, Shanghai, China

[2] School of Life Sciences, East China Normal University, Shanghai, China

* Corresponding author

E-mail: cyyao@tongji.edu.cn (CYY)

Abstract

Due to maternal inheritance and minimal rearrangement, the chloroplast genome is an important genetic resource for evolutionary studies. However, the evolutionary dynamics and phylogenetic performance of chloroplast genomes in closely related species are poorly characterized, particularly in taxonomically complex and species-rich group. The taxonomically unresolved *Ficus sarmentosa* species complex (Moraceae) comprises approximately 20 taxa with genetic background unclear. In this study, we explored the evolutionary dynamics, hotspot loci, and phylogenetic performance of thirteen chloroplast genomes (including eleven newly obtained and two downloaded from NCBI) representing the *F. sarmentosa* complex. Their sequence lengths, IR boundaries, repeat sequences, and codon usage were compared. Both sequence length and IR boundaries were found to be highly conserved. All four categories of long repeat sequences were found across all 13 chloroplast genomes, with palindromic and forward sequences being the most common. The number of simple sequence repeat (SSR) loci varied from 175 (*F. dinganensis* and *F. howii*) to 190 (*F. polynervis*), with the dinucleotide motif appearing the most frequently. Relative synonymous codon usage (RSCU) analysis indicated that codons ending with A/T were prior to those ending with C/T. The majority of coding sequence regions were found to have undergone negative selection with the exception of ten genes (*accD*, *clpP*, *ndhK*, *rbcL*, *rpl20*, *rpl22*, *rpl23*, *rpoC1*, *rps15*, and *rps4*) which exhibited potential positive selective signatures. Five hypervariable genic regions (*rps15*, *ycf1*, *rpoA*, *ndhF*, and *rpl22*) and five hypervariable intergenic regions (*trnH-GUG-psbA*, *rpl32-trnL-UAG*, *psbZ-trnG-GCC*, *trnK-UUU-rps*16 and *ndhF-rpl32*) were identified. Overall, phylogenomic analysis based on 123 *Ficus* chloroplast genomes showed promise for studying the evolutionary relationships in *Ficus*, despite cyto-nuclear discordance. Furthermore, based on the phylogenetic performance of the *F. sarmentosa* complex and *F.*

auriculata complex, the chloroplast genome also exhibited a promising phylogenetic resolution in closely related species.

Introduction

The genus *Ficus* L. (Moraceae) is a species-rich taxa which contains at least 800 species and is widely distributing across tropical and subtropical regions [1-3]. Due to insufficient genetic differentiation, the genus *Ficus* is taxonomically complex and contains many sympatric species, , including the *F. pedunculosa* group, *F. punctata* group, *F. chartacea* group, and *F. subulata* group, among others [1, 3, 4]. Meanwhile, widely not-strict one-to-one obligate mutualism between fig trees and fig wasps has results in frequent hybridization and introgression among *Ficus* species [5-8], which have so far hindered research on the taxonomy and evolutionary history [9]. Our current understanding of the *Ficus* phylogenetic framework is the result of research on a few nuclear loci, such as ITS, ETS, *G3pdh*, GBSSI, and *waxy* [3, 4, 10-14]. Although nuclear genome data have been used to reconstruct the *Ficus* phylogeny [15-17], these studies represent less than ten percent of *Ficus* species, which is unlikely to accurately represent the evolutionary relationship in the genus *Ficus*.

While the use of nuclear genome data has advantages for the detection of hybridization and introgression, organellar genomic resources are also of great importance to evolutionary research due to maternal inheritance as a single unit [18, 19]. In the last decade, due to the rapidly decreasing cost of whole-genome sequencing (WGS) and the development of chloroplast genomic assembling pipelines, such as GerOrganelle [20], Fast-Plast (https://github.com/mrmckain/Fast-Plast), NOVOPlasty [21], and ORG.asm (https://git.metabarcoding.org/org-asm/org-asm), plastome-based evolutionary research have become easier and more cost-effective [22-24]. However, chloroplast genomes are publicly available for less than five percent of species in *Ficus* [25]. Bruun-Lund et al. [26] published a novel and

innovative *Ficus* phylogenetic framework based on 59 newly obtained chloroplast genomes, and the results of which were obvious inconsistent with phylogenies based on the nuclear genome. Unfortunately, many of the genome sequences used by Bruun-Lund et al. contained gaps (an average of 7% missing data with a maximum of 65%), leading to difficulty in comparative chloroplast genomics and possible phylogenetic artifacts [27]. Even so, the chloroplast framework of Brunn-Lund et al. have been replicated by subsequent research generally with an extended dataset [17]. Overall, according to studies in both *Ficus* and the other taxa [28-30], it has been verified to be effective for chloroplast genomes to reconstrcut the phylogenetic reference at infra-, inter-generic, and higher ranks. However, little is known about the phylogenetic performance of complete chloroplast genomes in studies of closely related species or even infra-species, particularly in the taxonomically complex genera such as *Ficus*.

The *Ficus* subg. *Synoecia* sect. *Rhizocladus* subsect. *Plagiostigma* comprises approximately 10 species and 11 varieties which are widely distributed across east Asia, parapatric to the distribution center of *Ficus* southeast Asia [1, 3, 13, 16, 31]. Subsect. *Plagiostigma* is genetically unclear and taxonomically unresolved, forming the *Ficus sarmentosa* species complex [4]. After the first systematic treatment of the *F. sarmentosa* complex by Corner in 1960 and 1965 [32, 33], few studies have attempted to unravel the taxonomic complexity of this group with the exception of descriptions of several controversial taxa in 1980s [1, 34-37] (such as *F. dinganensis*, *F. guizhouensis*, and *F. polynervis*) and the rank elevation of some varieties (such as *F. pubigera* var. *anserina* and *F. sarmentosa* var. *thunbergii*) [38-40]. More recently, some of this phylogenetic ambiguity was resolved through the molecular work of Zhang et al. [4]. Zhang’s work resulted in the rank elevation of *F. pubigera* var. *anserina* and the discovery that *F. sarmentosa* is not monophyletic, complicating the

relationship between the complex and these previously described species in 1980s. However, because only three loci (ITS, ETS, and *G3pdh*) were used to resolve the genetic background of the complex, the results were neither stable nor high-resolution [4]. The inclusion of more variable genetic loci or genome data should be helpful to resolve the taxonomic uncertainty of the *F. sarmentosa* complex. In this study, we utilized both comparative chloroplast genomics and phylogenomics to characterize 1) the diversity of hotspot loci, 2) the variation among chloroplast genomes, and 3) the potential of chloroplast genomes to resolve the evolutionary relationships between closely related species of the F. sarmentosa species complex.

Materials and methods

Sample collection, DNA extraction and resequencing, and genome assembly and annotation

Healthy, young leaves were collected from the field in 2015-2021, and each was sealed in silica gel. Based on the phylogenetic relationships outlined in Zhang et al. [4], we sampled eleven taxa within the *F. sarmentosa* species complex in order to maximize genetic coverage. Detailed sample information is shown in S1 Table. All voucher specimens were stored at the herbarium of East China Normal University (HSNU).

Total DNA was extracted from 100 mg of dry leaf tissue using the CTAB method [41]. After quality detection with NanoDrop and Qubit 2.0, purified DNA samples were randomly ultrasonicated into ~350 bp segments, which were subsequently used to construct paired-end libraries. Whole-genome resequencing (WGS) was carried out using the Illumina NovaSeq 6000 platform, according to the PE150 sequencing strategy. Raw reads were filtered and cleaned according to the following criteria: reads containing > 10% unidentified nucleotides, > 50% low-quality bases (Q≤5), or adapter sequences

were omitted for further analyses. Finally, the chloroplast genome sequences of two more taxa in the *F. sarmentosa* complex, *F. sarmentosa* var. *henryi* (GenBank accession no. OL415083) and *F. dinganensis* (GenBank accession no. OK375500), were included for further comparative analyses.

Clean data were used to assemble the complete circular chloroplast genome with GetOrganelle v1.6.4 [20], utilizing the "embplant_pt" model. In the case that the output sequence was not circular, the R (number of runs) and w (word size) parameters were adjusted until circularity was achieved. Chloroplast genome annotation was carried out using PGA [42], with the default parameters. The chloroplast genome was visualized using the online OGDRAW tool (https://chlorobox.mpimp-golm.mpg.de/OGDraw.html) [43]. For consistency, the two supplementary plastome sequences (OL415083 and OK375500) were re-annotated according to the same routine.

Analysis of chloroplast genome structure

All 13 *F. sarmentosa* complex genomes were analyzed to determine the lengths and GC contents of the whole genomes, four quadripartite regions (large single copy (LSC), small single copy (SSC), and two inverted repeats (IRs)), and coding sequence (CDS) regions.

Analysis of IR contraction and expansion

The online R Shiny application IRscope (https://irscope.shinyapps.io/irapp/) [44] was used to examine and visualize the boundary variation of LSC/IR/SSC of all 13 *F. sarmentosa* complex genomes.

Analysis of long repeat sequences and SSRs

The four categories of long repeat sequences, forward (F), reverse (R), complement (C), and palindromic (P), were analyzed using the REPuter online tool (https://bibiserv.cebitec.uni-bielefeld.de/reputer) [45], with 50 maximum computed repeats and a minimal repeat size of 8. One of

the inverted repeat regions (IRb) was removed in the REPuter analysis to avoid repeatable results. Simple sequence repeats (SSRs) were detected using MISA (https://webblast.ipk-gatersleben.de/misa/) [46], with the following parameters: 8 repeat units for mononucleotide SSRs, 5 repeat units for dinucleotide SSRs, 4 repeat units for trinucleotide SSRs, and 3 repeat units for tetra-, penta-, and hexanucleotide SSRs. The maximal sequence length between two SSRs was set to 100 bp.

Comparison of complete chloroplast genomes and diversity hotspot analysis

All 13 *F. sarmentosa* complex genomes were compared using the mVISTA online tool (https://genome.lbl.gov/vista/mvista/submit.shtml) [47], with the global multiple alignment model (LAGAN). The *F. anserine* chloroplast genome was used as the reference and the RankVISTA probability threshold was set to 0.5. All genes and intergenic regions were extracted from the Genbank annotation files in batches using Perl scripts created by Xiao-Jian Qu (https://github.com/quxiaojian/Bioinformatic_Scripts). Alignments of the genic and intergenic loci were carried out using MUSCLE v5.1 [48], with default parameters. After alignment, the nucleotide diversity (π) was calculated for all genic and intergenic loci.

Codon usage analysis

To compare codon usage patterns of all the CDS sequences across 13 *F. sarmentosa* complex genomes, the relative synonymous codon usage (RSCU) was calculated using DAMBE v7.3.11 [49].

Selective pressure analysis

The ratio of nonsynonymous (Ka) to synonymous (Ks) substitution can be used to quantify evolutionary selective pressure. The Ka/Ks ratio was calculated for all 79 unique CDSs using TBtools v1.09876 [50]. Positive selection was indicated by Ka/Ks > 1, negative (purifying) selection was

indicated by Ka/Ks < 1, and neutral selection was indicated by Ka/Ks = 1. Based on the phylogenetic framework outlined in Zhang et al. [4], *F. simplicissima* was chosen as the reference to calculate Ka and Ks between *F. simplicissima* and our sampled representatives of the *F. sarmentosa* complex. For visualization purposes, the NaN value (i.e., both Ks and Ka = 0) was manually set as "1" to denote neutral selection. Finally, the infinity value (Ks > 0 and Ka = 0) was counted alone.

Phylogenetic analysis

The 13 *F. sarmentosa* complex chloroplast genomes were combined with 36 *Ficus* chloroplast genomes from GenBank and 59 *Ficus* genomes published by Bruun-Lund et al. [26]. Additionally, 18 genomes from the China National GeneBank (accession number: CNP0001337) and Genome Sequence Archive (accession number: PRJCA002187) [15, 51], including 8 samples belonging to the *F. sarmentosa* complex, were assembled to further explore the *Ficus* phylogenomics and the potential of chloroplast genomes to resolve the evolutionary relationships among the closely related species. Seven samples from the Olmedieae tribe were chosen as the outgroup, according to the previous studies [26, 52]. After discarding four samples found to be more than half (> 50%) missing data (three in the genus *Ficus* and one in the outgroup), a total of 129 chloroplast genomes were used to construct the phylogenetic tree (detailed samples information shown in S1 Table). Additionally, one of the two IRs was removed. The 129 genomes were aligned using MAFFT v7.490 [53], with the "auto" model. The aligned sequences were trimmed with trimAl and sites with > 10% gaps were removed [54]. The maximum likelihood (ML) tree was constructed using IQ-TREE 2 with ultrafast bootstrap (-bb) and aLRT test (-alrt) numbers set at 10000. The optimal nucleotide substitution model was chosen with ModelFinder [55].

Results

Summary of all 13 complete chloroplast genomes in the *F. sarmentosa* complex

For the eleven newly resequenced samples, a total of 79G bases were obtained from 19,379,830 (*F. sarmentosa* var. *sarmentosa*) to 23,945,593 (*F. sarmentosa* var. *henryi*) clean paired-end reads. The ratios of chloroplast paired reads to whole reads ranged from 0.87% (*F. sarmentosa* var. *henryi*) to 4.05% (*F. guizhouensis*). The average kmer-coverage values ranged from 41.5 (*F. sarmentosa* var. *henryi*) to 128.2 (*F. sarmentosa* var. *thunbergii* and *F. pubigera*). Detailed information on high-throughput sequencing data can be found in S2 Table.

All *F. sarmentosa* complex genomes, including 11 newly obtained genomes and two genomes downloaded from GenBank, exhibited a typical quadripartite structure (Fig 1), containing one LSC, one SSC, and two IRs. The lengths of the complete chloroplast genomes ranged from 160,018 (*F. dinganensis*) to 160,385 bp (*F. sarmentosa* var. *lacrymans*) (Table 1). The LSCs accounted for 55.12-55.20% of the total genome size and ranged from 88,200 (*F. dinganensis*) to 88,535 bp (*F. sarmentosa* var. *lacrymans*) in length. The SSCs accounted for 12.51-12.55% of the total genome size and ranged from 20,064 (*F. sarmentosa* var. *lacrymans*) to 20,108 bp (*F. pubigera*) in length. The IRs accounted for 16.14-16.17% of the total genome size and varied from 25,866 (*F. dinganensis*) to 25,898 bp (*F. sarmentosa* var. *nipponica* and *F. sarmentosa* var. *thunbergii*). The CDS regions ranged from 79,149 (*F. dinganensis*) to 79,308 bp (*F. sarmentosa* var. *impressa*) in accumulated lengths. The GC content of the whole genome ranged from 35.94 to 35.99 %, with *F. sarmentosa* var. *henryi* having the highest GC content, and *F. anserine* and *F. pubigera* having the lowest. The GC contents of LSCs, SSCs, and IRs were also calculated. Overall, the IRs had the highest GC content, which ranged from 42.63% (*F.*

anserina, *F. howii*, and *F. pubigera*) to 42.65% (*F. pumila*, *F. sarmentosa* var. *lacrymans*, and *F. sarmentosa* var. *sarmentosa*). SSCs had the lowest GC content, which ranged from 28.94% (*F. sarmentosa* var. *lacrymans* and *F. sarmentosa* var. *sarmentosa*) to 29.09% (*F. sarmentosa* var. *henryi*, *F. sarmentosa* var. *impressa*, and *F. sarmentosa* var. *nipponica*). The same to all 13 genomes, 131 genes were annotated, including 86 coding genes, 37 transfer RNA (tRNA) genes, and 8 ribosomal RNA (rRNA) genes. Finally, it was discovered that the *infA* gene was intensively pseudogenized [17].

Fig 1. Chloroplast gene maps of *Ficus sarmentosa* var. *sarmentosa*, *Ficus guizhouensis*, and *Ficus howii*. Genes drawn inside are transcribed clockwise and genes drawn outside are counterclockwise. Genes belonging to different functional groups are color coded. In the inner circle, dark gray and light gray indicate the GC content and AT content, respectively. The boundaries of the large single copy (LSC), small single copy (SSC), and two inverted regions (IRa, IRb) are also shown in the inner circle.

Table 1. Summary of complete chloroplast genomes of all thirteen taxa in the *F. sarmentosa* species complex.

Taxon	Total Length (bp)	LSC Length (bp)	SLC Length (bp)	IR Length (bp)	CDS Length (bp)	Total GC (%)	LSC GC (%)	SSC GC (%)	IR GC (%)
F. anserina	160267	88395	20106	25883	79173	35.94	33.61	28.97	42.63
F. dinganensis	160018	88200	20086	25866	79149	35.98	33.67	29.00	42.64
F. guizhouensis	160209	88330	20103	25888	79272	35.98	33.66	29.02	42.64
F. howii	160169	88328	20076	25882	79167	35.98	33.65	29.03	42.63
F. polynervis	160356	88507	20063	25893	79155	35.95	33.62	28.95	42.64

F. pubigera	160299	88425	20108	25883	79167	35.94	33.61	28.98	42.63
F. pumila	160229	88369	20100	25880	79152	35.98	33.64	29.08	42.65
F. sarmentosa* var. *henryi	160183	88307	20080	25898	79170	35.99	33.66	29.09	42.64
F. sarmentosa* var. *impressa	160293	88417	20080	25898	79308	35.97	33.63	29.09	42.64
F. sarmentosa* var. *lacrymans	160385	88535	20064	25893	79155	35.95	33.63	28.94	42.65
F. sarmentosa* var. *nipponica	160314	88436	20082	25898	79170	35.97	33.63	29.09	42.64
F. sarmentosa* var. *sarmentosa	160355	88501	20068	25893	79155	35.95	33.62	28.94	42.65
F. sarmentosa* var. *thunbergii	160282	88397	20089	25898	79179	35.98	33.65	29.06	42.64

IR contraction and expansion

Across all 13 *F. sarmentosa* complex genomes, four junctions among IR, LSC, and SSC regions were compared (Fig 2). Invariably and consistently across all 13 genomes, the boundary between the LSC and IRb regions (JLB) was located within the *rps19* gene, 171 bp away from its starting base and 108 bp away from its ending base. The boundary between the IRb and SSC regions (JSB) was located within the *ndhF* gene, either 17 or 25 bp (*F. pumila*) away from its starting base and 2,236 (*F. anserina*, *F. pubigera*, *F. sarmentosa* var. *henryi*, and *F. sarmentosa* var. *sarmentosa*), 2,237 (*F. pumila*), or 2,245 bp (all other taxa) away from its ending base. The boundary between the SSC and IRa regions (JSA) was located within the *ycf1* gene, either 4,713, 4,722, or 4,724 bp away from its starting base and 1,024 (*F. pumila*) or 1,026 bp away from its ending base. For the IRa and LSC regions, the junction between them was located between the *rpl2* and *trnH* genes, either 62 or 63 bp away from the starting base of the *trnH* gene.

Fig 2. Comparison of the boundaries between the large single copy (LSC), small single copy

(SSC), and two inverted repeat regions (IRs) among 13 chloroplast genomes in the *F. sarmentosa* complex. The numbers around the vertical lines indicate the distances between the boundaries and the starting or ending bases of their nearest genes.

Long repeat sequences and simple sequence repeats (SSRs)

Across all 13 *F. sarmentosa* complex genomes, a total of 373 long repeat sequences were identified, representing all four repeat categories: forward (F), reverse (R), complement (C), and palindromic (P) (Fig 3A). All four categories of repeats were detected within all 13 chloroplast genomes. Four taxa, *F. anserina*, *F. dinganensis*, *F. polynervis*, and *F. sarmentosa* var. *sarmentosa*, contained the greatest number of long repeat sequences (31), while *F. sarmentosa* var. *thunbergii* contained the least (26). Among all four repeats, P repeats were the most common across all 13 genomes, ranging from 14 (*F. guizhouensis*) to 16. There were relatively fewer R and C repeats, with *F. polynervis*, *F. sarmentosa* var. *lacrymans*, and *F. sarmentosa* var. *sarmentosa* containing the most (4). For the lengths of long repeat sequences, 30-39 bp is the most common ranging from 21 (*F. sarmentosa* var. *thunbergii*) to 26 (*F. anserina* and *F. dinganensis*) times (Fig 3 B). The long repeat sequences over 60 bp in length was the least, appearing in only once. The maximum length among all the long repeat sequences was 64 bp.

Fig 3. Comparison of long repeat sequences among 13 *F. sarmentosa* complex genomes. A, The number of each of four long repeat types (P, palindromic; F, forward; R; reverse; C complement); B, The number of long repeat sequences of different lengths.

Simple sequence repeats (SSRs) consisting of the 1- to 6- nucleotide motifs were surveyed across the 13 *F. sarmentosa* complex genomes (Fig 4 A). Mononucleotide SSRs were the most abundant (78.71%) across all 13 genomes. Dinucleotide SSRs, the most commonly used motif in population genetics and phylogenetics, were also relatively abundant, appearing from 19 (*F. howii*, *F. polynervis*, *F. sarmentosa* var. *lacrymans*, and *F. sarmentosa* var. *sarmentosa*) to 22 (*F. pumila* and *F. sarmentosa* var. *thunbergii*) times. It is noteworthy that tetranucleotide SSRs were more common than trinucleotide SSRs (Fig 4 A), considering the former appeared from 9 (*F. guizhouensis*, *F. polynervis*, *F. sarmentosa* var. *lacrymans*, and *F. sarmentosa* var. *sarmentosa*) to 11 (*F. pumila*, *F. sarmentosa* var. *henryi*, *F. sarmentosa* var. *impressa*, and *F. sarmentosa* var. *nipponica*) times. Hexanucleotide SSRs were absent from all genomes. The total number of SSRs varied from 175 (*F. dinganensis* and *F. howii*) to 190 (*F. polynervis*). Of the repeat motifs, A/T was the most common mononucleotide motif, appearing from132 (*F. guizhouensis*) to 149 (*F. polynervis* and *F. sarmentosa* var. *sarmentosa*) times (Fig 4 B). AT/AT was the second most common motif, appearing between 18 (*F. howii*, *F. polynervis*, *F. sarmentosa* var. *lacrymans*, and *F. sarmentosa* var. *sarmentosa*) and 21 (*F. pumila* and *F. sarmentosa* var. *thunbergii*) times. The marjority of the ~~remainding~~ motifs appeared only once to four times, with the exception of AATT/AATT appearing up to seven times (Fig 4 B).

Fig 4. Comparison of simple sequence repeats (SSRs) among 13 *F. sarmentosa* complex genomes. A, The number of SSRs containing one- to five- nucleotide motifs; B, The number of different SSR motifs.

Codon Usage

Across all 13 *F. sarmentosa* complex genomes, the 79 unique protein-coding CDS regions were encoded by between 22,867 (*F. polynervis* and *F. sarmentosa* var. *lacrymans*) and 22,918 (*F. sarmentosa* var. *impressa*) codons. The codon usage among all 81 protein-coding genes is summarized in Table 2. Among these codons, CAU encoding histidine (H) was the most frequent, appearing 12,800 times across all 13 taxa. Except the stop codon, UGU encoding L-Cysteine (C) was the next rarest, appearing 718 times only. According to the RSCU analysis, GCU and CUU had the highest average values of 1.858 and 1.818, respectively (Table 2), whereas UAC and CGC had the lowest average values of 0.364 and 0.400, respectively. Among all three stop codons, UAA was the most common (53.64%). Thirty out of the 64 codons with RSCU > 1 ended with either A or U, while 32 out of 64 codons with RSCU < 1 ended with either G or C, with the exception of the AUA codon.

Table 2. The relative synonymous codon usage (RSCU) of all 64 codons. The taxa are represented by the numbers indicated in Table 1.

Codon	AA	RSCU (relative synonymous codon usage)												
		1	2	3	4	5	6	7	8	9	10	11	12	13
UGA	*	0.662	0.662	0.662	0.662	0.662	0.662	0.662	0.662	0.662	0.662	0.662	0.662	0.662
UAG	*	0.701	0.701	0.74	0.701	0.74	0.701	0.74	0.74	0.74	0.74	0.74	0.74	0.74
UAA	*	1.636	1.636	1.597	1.636	1.597	1.636	1.597	1.597	1.597	1.597	1.597	1.597	1.597
GCU	A	1.853	1.853	1.853	1.853	1.862	1.853	1.854	1.861	1.863	1.869	1.861	1.862	1.857
GCG	A	0.413	0.413	0.413	0.413	0.415	0.413	0.412	0.404	0.404	0.411	0.404	0.415	0.413
GCC	A	0.594	0.594	0.6	0.597	0.589	0.594	0.592	0.591	0.591	0.589	0.591	0.589	0.59
GCA	A	1.14	1.14	1.135	1.136	1.134	1.14	1.142	1.144	1.143	1.132	1.144	1.134	1.141
UGU	C	1.57	1.575	1.569	1.569	1.561	1.561	1.569	1.569	1.569	1.561	1.569	1.561	1.569
UGC	C	0.43	0.425	0.431	0.431	0.439	0.439	0.431	0.431	0.431	0.439	0.431	0.439	0.431
GAU	D	1.596	1.596	1.596	1.596	1.596	1.598	1.594	1.595	1.595	1.597	1.595	1.598	1.593
GAC	D	0.404	0.404	0.404	0.404	0.404	0.402	0.406	0.405	0.405	0.403	0.405	0.402	0.407
GAG	E	0.462	0.467	0.464	0.464	0.459	0.463	0.464	0.465	0.466	0.461	0.465	0.459	0.463
GAA	E	1.538	1.533	1.536	1.536	1.541	1.537	1.536	1.535	1.534	1.539	1.535	1.541	1.537
UUU	F	1.368	1.367	1.374	1.367	1.373	1.369	1.372	1.37	1.37	1.372	1.371	1.373	1.37
UUC	F	0.632	0.633	0.626	0.633	0.627	0.631	0.628	0.63	0.63	0.628	0.629	0.627	0.63
GGU	G	1.34	1.34	1.338	1.338	1.341	1.334	1.342	1.337	1.338	1.342	1.337	1.341	1.342
GGG	G	0.609	0.606	0.61	0.612	0.612	0.609	0.609	0.612	0.61	0.613	0.612	0.612	0.609
GGC	G	0.404	0.402	0.404	0.407	0.397	0.407	0.402	0.405	0.404	0.396	0.405	0.397	0.402
GGA	G	1.647	1.652	1.648	1.643	1.651	1.649	1.647	1.647	1.648	1.649	1.647	1.651	1.647
CAC	H	0.463	0.468	0.46	0.465	0.457	0.463	0.465	0.464	0.464	0.457	0.464	0.457	0.464
CAU	H	1.537	1.532	1.54	1.535	1.543	1.537	1.535	1.536	1.536	1.543	1.536	1.543	1.536
AUU	I	1.471	1.471	1.471	1.477	1.47	1.473	1.471	1.468	1.467	1.47	1.468	1.47	1.47
AUA	I	0.965	0.967	0.959	0.965	0.965	0.966	0.962	0.964	0.963	0.963	0.964	0.965	0.964
AUC	I	0.564	0.562	0.57	0.558	0.565	0.561	0.567	0.568	0.57	0.567	0.568	0.565	0.566
AAA	K	1.531	1.534	1.534	1.533	1.528	1.53	1.534	1.536	1.537	1.527	1.536	1.528	1.539
AAG	K	0.469	0.466	0.466	0.467	0.472	0.47	0.466	0.464	0.463	0.473	0.464	0.472	0.461
CUA	L	1.162	1.16	1.169	1.166	1.145	1.169	1.158	1.155	1.154	1.146	1.155	1.145	1.16
CUC	L	0.521	0.522	0.528	0.518	0.52	0.521	0.518	0.516	0.516	0.52	0.516	0.52	0.515
CUG	L	0.504	0.501	0.5	0.501	0.513	0.504	0.504	0.506	0.505	0.513	0.506	0.513	0.505
CUU	L	1.813	1.816	1.802	1.814	1.823	1.806	1.82	1.823	1.825	1.821	1.823	1.823	1.819
UUA	L	1.249	1.251	1.25	1.249	1.254	1.249	1.25	1.254	1.254	1.251	1.254	1.252	1.25
UUG	L	0.751	0.749	0.75	0.751	0.746	0.751	0.75	0.746	0.746	0.749	0.746	0.748	0.75
AUG	M	1	1	1	1	1	1	1	1	1	1	1	1	1
AAC	N	0.446	0.444	0.442	0.446	0.452	0.446	0.444	0.443	0.443	0.454	0.443	0.453	0.445
AAU	N	1.554	1.556	1.558	1.554	1.548	1.554	1.556	1.557	1.557	1.546	1.557	1.547	1.555
CCA	P	1.166	1.16	1.165	1.163	1.154	1.166	1.162	1.162	1.165	1.158	1.162	1.154	1.16
CCC	P	0.68	0.681	0.679	0.677	0.678	0.676	0.68	0.684	0.684	0.678	0.684	0.678	0.684
CCU	P	1.592	1.596	1.586	1.598	1.596	1.597	1.592	1.592	1.591	1.596	1.592	1.596	1.591
CCG	P	0.562	0.563	0.57	0.562	0.573	0.562	0.566	0.562	0.561	0.568	0.562	0.573	0.565
CAA	Q	1.585	1.588	1.58	1.586	1.587	1.585	1.582	1.585	1.583	1.584	1.585	1.587	1.582
CAG	Q	0.415	0.412	0.42	0.414	0.413	0.415	0.418	0.415	0.417	0.416	0.415	0.413	0.418
AGA	R	1.496	1.5	1.5	1.497	1.502	1.494	1.496	1.499	1.501	1.502	1.499	1.502	1.497
AGG	R	0.504	0.5	0.5	0.503	0.498	0.506	0.504	0.501	0.499	0.498	0.501	0.498	0.503
CGA	R	1.6	1.596	1.604	1.596	1.602	1.601	1.596	1.591	1.594	1.602	1.591	1.602	1.596
CGC	R	0.405	0.399	0.398	0.399	0.403	0.399	0.399	0.399	0.398	0.403	0.399	0.403	0.399
CGG	R	0.426	0.425	0.419	0.425	0.423	0.43	0.414	0.419	0.419	0.423	0.419	0.423	0.414
CGU	R	1.569	1.581	1.579	1.581	1.571	1.57	1.591	1.591	1.589	1.571	1.591	1.571	1.591
AGC	S	0.447	0.456	0.455	0.46	0.451	0.45	0.454	0.454	0.454	0.45	0.454	0.447	0.454
AGU	S	1.553	1.544	1.545	1.54	1.549	1.55	1.546	1.546	1.546	1.55	1.546	1.553	1.546
UCA	S	1.111	1.111	1.115	1.114	1.116	1.107	1.111	1.109	1.108	1.116	1.11	1.116	1.109
UCC	S	0.817	0.817	0.809	0.817	0.803	0.814	0.806	0.812	0.816	0.8	0.813	0.803	0.808
UCG	S	0.449	0.449	0.448	0.452	0.448	0.452	0.45	0.448	0.447	0.448	0.448	0.448	0.446
UCU	S	1.624	1.622	1.628	1.617	1.632	1.627	1.632	1.631	1.629	1.635	1.629	1.632	1.636
ACC	T	0.701	0.699	0.701	0.701	0.701	0.701	0.702	0.695	0.697	0.702	0.695	0.701	0.699
ACA	T	1.187	1.189	1.182	1.185	1.177	1.185	1.176	1.177	1.179	1.179	1.177	1.177	1.174
ACG	T	0.417	0.416	0.422	0.417	0.417	0.417	0.422	0.426	0.425	0.418	0.426	0.417	0.426
ACU	T	1.695	1.696	1.695	1.697	1.706	1.697	1.7	1.701	1.698	1.702	1.701	1.706	1.701
GUU	V	1.463	1.462	1.461	1.468	1.466	1.466	1.461	1.458	1.461	1.46	1.458	1.466	1.461
GUG	V	0.513	0.511	0.522	0.513	0.513	0.51	0.513	0.515	0.521	0.516	0.515	0.513	0.513
GUC	V	0.405	0.406	0.405	0.403	0.409	0.405	0.406	0.411	0.407	0.415	0.411	0.409	0.406
GUA	V	1.618	1.621	1.613	1.616	1.612	1.618	1.62	1.616	1.612	1.609	1.616	1.612	1.62
UGG	W	1	1	1	1	1	1	1	1	1	1	1	1	1
UAC	Y	0.365	0.364	0.364	0.365	0.364	0.361	0.366	0.366	0.365	0.364	0.366	0.364	0.363
UAU	Y	1.635	1.636	1.636	1.635	1.636	1.639	1.634	1.634	1.635	1.636	1.634	1.636	1.637

Genomic divergence and hotspot regions

The divergence of whole sequence among the 13 *F. sarmentosa* complex genomes was analyzed using the mVISTA online platform with *F. anserina* as a reference. The results showed that the full-length chloroplast genomes were largely conserved across all 13 taxa. The majority of variable sites were located in intergenic spacer regions (marked red in Fig 5). Interestingly, IRs were found to be more conserved than either LSCs or SSCs.

Fig 5. Comparison of complete chloroplast genomes among 13 taxa in the *F. sarmentosa* complex with *F. anserina* as a reference. Thick, gray arrows above the alignment indicate the orientation and position of each gene. A cut-off of 70% identity was chosen for the plots. The Y-axis represents the identity percentage, ranging from 50 to 100%.

Nucleotide diversity (π) was calculated for each gene and intergenic region to evaluate genetic differentiation and detect hyper-variable segments. Among 60 genes > 200 bp in length, *rps15*, *ycf1*, *rpoA*, *ndhF*, and *rpl22* exhibited the highest nucleotide diversity: 0.00535, 0.00462, 0.00456, 0.00427 and 0.00377, respectively (Fig 6). The alignment lengths of these five genes ~~radically~~ ranged from 282 to 5,748 bp. Seven genes (*atpI*, *psbE*, *rpl33*, *rps18*, *psbH*, *rrn23*, and *psaC*) were found to be identical, with a nucleotide diversity of zero. The average diversity of the 60 genes was 0.001676, while the average diversity of intergenic spacer regions was 0.00435, approximately 2.6 times that of the genes (Fig 6). Among the intergenic spacer regions, the highest nucleotide diversity was exhibited by *trnH-GUG-psbA* (0.01458), followed by *rpl32-trnL-UAG* (0.01225), *psbZ-trnG-GCC* (0.01148), *trnK-UUU-rps*16 (0.01144), and *ndhF-rpl32* (0.01112). The alignment lengths of these regions ranged from 366 to

1,829 bp (Fig 6).

Fig 6. Nucleotide diversity of genes and intergenic spacer regions among 13 taxa in the *F. sarmentosa* complex. The alignment lengths are indicated on the bars. The horizontal lines indicate the average nucleotide diversity of genes and intergenic spacer regions, respectively. The top five genes or intergenic spacers with the highest nucleotide diversity are highlighted in blue.

Selective pressure analysis

The ratio of nonsynonymous (Ka) to synonymous (Ks) ~~substitution~~ was calculated to quantify the evolutionary selective pressure on the *F. sarmentosa* complex, with *F. simplicissima* used as the reference genome. Overall, Ka/Ks ratios of most genes were < 1 (Fig 7). Additionally, twenty genes were found to contain no substitutions, i.e., both Ka and Ks are zero (shown as "1" in Fig 7). However, five genes (*accD*, *matK*, *ndhF*, *rpoA*, and *ycf1*) had partial Ka/Ks ratios over 1, which are potential signals of positive selection. An infinite Ka/Ks ratios (Ka > 0 and Ks = 0) existed in 27 genes (Fig 7). Notably, ten genes (*accD*, *clpP*, *ndhK*, *rbcL*, *rpl20*, *rpl22*, *rpl23*, *rpoC1*, *rps15*, and *rps4*) possessed infinite Ka/Ks ratios in more than half of the taxa.

Fig 7. Boxplot of Ka/Ks ratios for 80 unique CDS regions. The value 1.0 represents the situation where both Ka and Ks equal zero. The line chart superimposed upon the boxplot demonstrates the frequency of infinite Ka/Ks ratios (Ka > 0 and Ks = 0), with detailed numbers labeled simultaneously.

Phylogenetic analysis

The chloroplast phylogenomic ML tree illustrated a well-supported phylogenetic relationship in the genus *Ficus* (Fig 8). Subgenus *Pharmacosycea* sect. *Pharmacosycea* was strongly supported as a sister group to the rest of the genus *Ficus* (SH-aLRT = 100 and MLBS = 100) Additionally, a clade including subg. *Urostigma* sect. *Galoglychia* and *Americana*, subg. *Sycomorus* sect. *Sycomorus*, and a few species of subg. *Pharmacosycea* sect. *Oreosycea* (subser. *Ablbipilae* in Corner's system) (clade A) was found to be sister to the remainder of *Ficus* except sect. *Pharmacosycea*.Aside from these two clades, the remaining *Ficus* taxa formed three clades (clades B, C, and D in Fig 8), with unstable support (SH-aLRT = 64.9 and MLBS = 73). Clade B (SH-aLRT = 99.9 and MLBS = 100) was comprised of five different subgenera, while clade C (SH-aLRT = 100 and MLBS = 100) included all species of subg. *Sycidium* as well as members of each of the other five subgenera. Clade D (SH-aLRT = 100 and MLBS = 100) contained only two species in subg. *Pharmacosycea* sect. *Oreosycea*. On the whole, all six subgenera were found not to be monophyletic.

Fig 8. **The maximum likelihood (ML) phylogenetic tree of 123 chloroplast genomes in *Ficus* with six Olmoideae genomes as the outgroup.** Only the branches with either SH-aLRT or ultrafast bootstrap < 95% were annotated by corresponding values. The starred tip names indicate genomes obtained from Bruun-Lund et al.; the red names indicate genomes obtained in this study; the blue names indicate members of the *F. auriculata* complex; and the bold names indicate members of the *F. sarmentosa complex*. The subgenus and section division of *Ficus* are annotated to the right of tip names. The topology of the ML tree is shown in the upper left corner (excluding the outgroup).

With the inclusion of eleven additional samples, a phylogenomic analysis was carried out on 24

samples representing the 13 taxa in the *F. sarmentosa* complex (Fig 8). The results showed that the *F. sarmentosa* complex failed to form a monophyletic group. Except three samples which are unexpectedly embedded in a distinct clade with other members of subg. *Synoecia* (clade E), two distinct lineages were recognized, with the majority of nodes within these two lineages being well-supported (clades F and G). The six *F. sarmentosa* varieties were found to be scattered across clades E, F, and G, and only two individuals of *F. sarmentosa* var. *lacrymans* clustered together. The six *F. pumila* samples were also found not to be a monophyletic group embedded by *F. sarmentosa* var. *thunbergii*.

Discussion

The differentiation and diversity of the chloroplast genome in the *F. sarmentosa* complex

To date, vast comparative chloroplast genomic studies have been conducted in a wide range of taxonomic levels, such as order (such as Dipsacales [56] and Saxifragales [57]), family (such as Orchidaceae [58] and Zingiberaceae [59]), and genus (such as *Camellia* [60], *Lindera* [61], *Gossypium* [62], and *Ficus* [17, 51, 63]). However, less research has focused on the comparative genomics of taxa undergoing recent speciation, such as the species complex [29]. Although the structural conservation of the chloroplast genome at low taxonomic levels is well-characterized [64-67], the detailed patterns of genomic differentiation and diversity among closely related species remain largely unknown. Therefore, comprehensive comparisons between closely related species are necessary to improve our understanding of the mechanisms, rates, or directionality of genome evolution during the early stages after speciation [29].

In this study, we investigated the evolutionary dynamics of thirteen high-quality chloroplast

genomes from the *F. sarmentosa* complex. Overall, the length of both whole-genome and quadripartite regions were quite similar among taxa, with only 0.2288%, 0.3784%, 0.2188%, and 0.1236% variation among whole genomes, LSCs, SSCs, and IRs, respectively (Table 1, Fig 1). Furthermore, the number, content, and orientation of annotated genes among all 13 genomes were identical. The IR boundaries (JLB, JSB, JSA, and JLA) were also relatively coincident among the 13 genomes, being located at the same loci with only slight variation in the distance to the starting or ending bases. For long repeat sequences, all four repeat units were shared among all 13 plastomes, and the number of repeat units and their proportions exhibited only slight differentiation. For example, the proportion of palindromic sequences ranged from 45.16 to 61.54% (Fig 3). Similarly, in SSR regions, the proportion of the mononucleotide repeat units ranged from 72.63 to 85.96% (Fig 4).

The high conservation exhibited across the *F. sarmentosa* complex is consistent with other studies on closely related taxa. For example, a study of 22 closely related *Oryza* species indicated that conservation was common at lower taxonomic levels [29]. A study of four peanut varieties serves as a more extreme example, reporting perfectly identical IR boundary junction positions [68]. Although comparative chloroplast genomic studies have rarely focused on closely related species, the high conservation of chloroplast genome among closely related species is recognized based on our work and other related research.

Chloroplast genomic evolutionary hotspots

Although chloroplast genomes exhibit high conservation among closely related species, discrepancy and heterogeneity have also been widely observed across the whole genomes. Overall, the variable sites of the two single-copy regions (LSC and SSC) are more abundant than IR regions in both the genus *Ficus* (Fig 5) and most other plant groups [56, 69, 70]. Chloroplast regions with different mutation rates are

appropriate for a range of evolutionary research. In general, conservative coding genes are suitable for deep phylogenetic inferences at the family-level [28, 71, 72] or higher [73-75], whereas highly variable regions are appropriate for studies of biogeography, species delimitation, population genetics, and phylogenetic reconstruction at lower infra-generic levels [76, 77].

To date, more than 20 regions have been recommended as alternative loci for phylogenetics, species delimitation, and barcoding, including *matK*, *rbcL*, *trnH-psbA*, *ycf1*, *ycf1-ndhF*, among others [77-79]. However, these loci ~~appeared poor at the aspect of~~ informative sites in the *F. sarmentosa* complex (Fig 6), suggesting that evolutionary heterogeneity of chloroplast loci may be relatively common among different plant groups. In this study, we mined five hyper-variable intergenic regions at the level of the species complex, i.e., *trnH-GUG-psbA*, *rpl32-trnL-UAG*, *psb*Z-*trn*G-*GCC*, *trnK-UUU-rps16* and *ndhF-rpl32*. However, other studies of comparative chloroplast genomics in *Ficus* identified entirely different hyper-variable intergenic regions. For example, Xia et al. [63] identified *trnS-GCU-trnG-UCC*, *trnT-GGU-psbD*, *trnV-UAC-trnM-CAU*, *clpP-psbB*, *ndhF-trnL-UAG*, *trnL-UAG-ccsA*, *ndhD-psaC*, and *ycf1*, and Zhang et al. [17] identified t*rnL-UAG-rpl32*, *trnE-UUC-psbD*, *trnK-UUU-rps16*, *rpoB-trnC-GCA*, and *petN-psbM*. These disparate results suggest that the evolutionary dynamics of chloroplast genomes ~~vary~~ across both groups and taxonomic ranks. Therefore, we suggest that chloroplast loci should be chosen cautiously according to the research objective, plant group, and taxonomic level.

The plastomic phylogeny of the genus *Ficus* and the phylogenetic performance in closely related species

Based on a compilation of nearly all available *Ficus* chloroplast genomes, we obtained a robust ML phylogenetic tree with the majority of nodes exhibiting high bootstrap and SH-aLRT values, particularly the deep nodes (Fig 8). Overall, our ML phylogenetic tree is largely consistent with previous research

[17, 26], including the systematic position of subg. *Pharmacosycea* sect. *Pharmacosycea* and the mysterious displacment of certain individuals (*F. fulva, F. magnoliifolia, F. albipila, F. albert-smithii,* and *F. pumila*). However, analysis of our extended dataset highlighted an increase in non-monophyletic groups, such as subg. *Urostigma* sect. *Urostigma* and *Conosycea* (Fig 8). Notably, there were numerous incongruences between the chloroplast cladogram and previously published nuclear trees. For example, the chloroplast-based tree failed to support either *Ficus* subg. *Sycomorus* or subg. *Sycidium* as monophyletic groups, whereas these groups are well-confirmed in nuclear phylogenies [3, 12-14, 80]. Subg. *Synoecia* was also divided into three different clades (Fig 8). Further research into the displaced species, such as *F. fulva*, *F. ablert-smithii*, *F. magnoliilolia*, and *F. albipila*, may reveal host shifts or nonspecific pollination between fig trees and fig wasps [26]. Considering that a stable and comprehensively-sampled nuclear phylogenomic framework for the genus *Ficus* is still lacking, precise identification of these hybridization events will require more robust nuclear genome data as well as data from the associated fig wasps.

Compared to the nuclear phylogeny [4], we discovered a disparate evolutionary relationship among taxa in the *F. sarmentosa* complex, including three distinct clades (Fig 8, clades E, F, and G). Three samples from the complex dispersed into the clade E mixing with *F. sagittata* and the members of subg. *Synoecia* sect. *Apiosycea*. Unless more data support hybridization, misidentification may be an alternative explanation. Neither geographic nor morphological traits could be detected to support the split of clades F and G. Hybridization between *F. pumila* and *F. sarmentosa* var. *thunbergii* might exist, considering that the latter embedded into the former within clade G. Although our current samples are insufficient to fully resolve the taxonomy of the *F. sarmentosa* complex, the phylogenetic resolution provided by chloroplast genomes in the complex appears to be promising, as almost all the nodes are

strongly supported. The chloroplast genome has also shown high discriminability across the *F. auriculata* complex (Fig 8, blue labels) [81-83]. The relationships between four taxa (*F. auriculata*, *F. oligodon*, *F. hainanensis*, and *F. beipeiensis*) in the complex were well-resolved, while *F. beipeiensis* shared a distinct phylogenetic relationship with the climbing fig tree *F. tikoua*. Moreover, a new linage (*F. northern*) was identified based on chloroplast genomics, suggesting a promising segue into further exploration of the cryptic species (Fig 8, blue labels) [17]. Chloroplast genomes have been used to reconstruct high-resolution phylogenetic trees in other closely related species groups, such as peanut [84], rice [29], and Orchardgrass [85]. Taken together, the chloroplast genome appears to be a promising tool for exploring the evolutionary relationships between closely related species and even species complexes, although cyto-nuclear discordances often exist.

Conclusions

In this study, eleven *F. sarmentosa* complex chloroplast genomes were newly sequenced and characterized. Sequence lengths, IR boundaries, repeat sequences, and codon usage were compared among these eleven, and two previously-reported, chloroplast genomes, indicating that these parameters were highly conserved across taxa. However, heterogeneity was found in both nucleotide diversity and selective pressure among segments. We characterized ten evolutionary hotspot regions (*rps15*, *ycf1*, *rpoA*, *ndhF*, *rpl22*, *trnH*-GUG-*psbA*, *rpl32-trnL-UAG*, *psbZ-trnG-GCC*, *trnK-UUU-rps*16 and *ndhF-rpl32*). Phylogenomic analysis indicated that chloroplast genomes show promise for inferring the phylogenetic relationships between closely related groups, despite cyto-nuclear discordance.

Acknowledgments

We are grateful to Dr. Hong-Qing Li at East China Normal University, Dr. Zhi-Hui Su and Ms. Sasaki Ayako at Osaka University, Dr. Yong Chen and Dr. Kai-Liang Liu at Ningde Normal University, and Mr. Zhen Liu at Forestry and Grassland Administration of Motuo County for their support in wild collecting. The authors would like to thank TopEdit (www.topeditsci.com) for its linguistic assistance during the preparation of this manuscript.

Funding

This research was funded by National Natural Science Foundation of China from Dr. De-Shun Zhang (grant number 32071824). The funder De-Shun Zhang implemented the conceptualization, visualization, and draft review of this study.

Competing Interests

NO authors have competing interests.

Data Availability Statement

The eleven newly obtained complete chloroplast genome sequences that support the findings in the study are deposited in the NCBI with accession numbers as followed: ON257105-ON257115. The corresponding high throughput Illumina sequencing reads are deposited to Genome Sequence Archive (GSA) in BIG Data Center (https://bigd.big.ac.cn/gsa/) under BioProject accession: PRJCA012975.

Author Contributions

Conceptualization: Chi-Yuan Yao, De-Shun Zhang.

Data curation: Lu Zou.

Formal analysis: Zhen Zhang.

Funding acquisition: De-Shun Zhang.

Investigation: Zhen Zhang.

Methodology: Zhen Zhang.

Project administration: Chi-Yuan Yao.

Resources: Chi-Yuan Yao.

Software: Zhen Zhang.

Writing—original draft: Zhen Zhang.

Writing—review & editing: Zhen Zhang, De-Shun Zhang, Lu Zou, and Chi-Yuan Yao.

Visualization: De-Shun Zhang.

References

1. Berg CC, Corner EJH. Moraceae (*Ficus*). In: Nooteboom HP, editor. Flora Malesiana. 17. Leiden: National Herbarium of the Netherlands; 2005. p. 1-730.
2. Clement WL, Weiblen GD. Morphological evolution in the mulberry family (Moraceae). Syst Bot. 2009;34(3):530-52. doi: 10.1600/036364409789271155.
3. Clement WL, Bruun-Lund S, Cohen A, Kjellberg F, Weiblen GD, Rønsted N. Evolution and classification of figs (*Ficus*, Moraceae) and their close relatives (Castilleae) united by involucral

bracts. Bot J Linn Soc. 2020;193(3):316-39. doi: 10.1093/botlinnean/boaa022

4. Zhang Z, Wang XM, Liao S, Zhang JH, Li HQ. Phylogenetic reconstruction of *Ficus* subg. *Synoecia* and its allies (Moraceae), with implications on the origin of the climbing habit. Taxon. 2020;69(5):927-45. doi: 10.1002/tax.12282.

5. Machado CA, Robbins N, Gilbert MTP, Herre EA. Critical review of host specificity and its coevolutionary implications in the fig/fig-wasp mutualism. P Natl Acad Sci USA. 2005;102(suppl 1):6558-65. doi: 10.1073/pnas.0501840102.

6. Wachi N, Ku sumi J, Tzeng HY, Su ZH. Genome- wide sequence data suggest the possibility of pollinator sharing by host shift in dioecious figs (Moraceae, *Ficus*). Mol Ecol. 2016;25(22):5732-46. doi: 10.1111/mec.13876.

7. Wang G, Cannon CH, Chen J. Pollinator sharing and gene flow among closely related sympatric dioecious fig taxa. P Roy Soc B-Biol Sci. 2016;283(1828):20152963. doi: 10.1098/rspb.2015.2963.

8. Yu H, Liao YL, Cheng YF, Jia YJ, Compton SG. More examples of breakdown the 1:1 partner specificity between figs and fig wasps. Bot Stud. 2021;62(1):1-12. doi: 10.1186/s40529-021-00323-8.

9. Yu H, Zhao NX, Yao J, Chen YZ. The influence of species-specific coevolution of figs and fig wasps on *Ficus* (Moraceae) classification. J Trop Subtrop Bot. 2006;14(5):439-43. doi: 10.3969/j.issn.1005-3395.2006.5.014.

10. Rønsted N, Weiblen GD, Cook JM, Salamin N, Machado CA, Savolainen V. 60 million years of co-divergence in the fig–wasp symbiosis. P Roy Soc B-Biol Sci. 2005;272(1581):2593-9. doi: 10.1098/rspb.2005.3249.

11. Rønsted N, Weiblen GD, Clement W, Zerega N, Savolainen V. Reconstructing the phylogeny of figs (*Ficus*, Moraceae) to reveal the history of the fig pollination mutualism. Symbiosis. 2008;45:45-55.

12. Xu L, Harrison RD, Yang P, Yang DR. New insight into the phylogenetic and biogeographic history of genus *Ficus*: Vicariance played a relatively minor role compared with ecological opportunity and dispersal. J Syst Evol. 2011;49(6):546-57. doi: 10.1111/j.1759-6831.2011.00155.x.

13. Cruaud A, Rønsted N, Chantarasuwan B, Chou LS, Clement WL, Couloux A, et al. An extreme case of plant–insect codiversification: figs and fig-pollinating wasps. Syst Biol. 2012;61(6):1029-47. doi: 10.1093/sysbio/sys068.

14. Zhang Q, Onstein RE, Little SA, Sauquet H. Estimating divergence times and ancestral breeding systems in *Ficus* and Moraceae. Ann Bot. 2019;123(1):191-204. doi: 10.1093/aob/mcy159.

15. Zhang XT, Wang G, Zhang SC, Chen S, Wang YB, Wen P, et al. Genomes of the banyan tree and pollinator wasp provide insights into fig-wasp coevolution. Cell. 2020;183(4):875-89. e17. doi: 10.1016/j.cell.2020.09.043.

16. Rasplus JY, Rodriguez LJ, Sauné L, Peng YQ, Bain A, Kjellberg F, et al. Exploring systematic biases, rooting methods and morphological evidence to unravel the evolutionary history of the genus *Ficus* (Moraceae). Cladistics. 2021;37(4):402-22. doi: 10.1111/cla.12443.

17. Zhang ZR, Yang X, Li WY, Peng YQ, Gao J. Comparative chloroplast genome analysis of *Ficus* (Moraceae): Insight into adaptive evolution and mutational hotspot regions. Front Plant Sci. 2022;13:1-17. doi: 10.3389/fpls.2022.965335.

18. Soltis DE, Albert VA, Savolainen V, Hilu K, Qiu YL, Chase MW, et al. Genome-scale data,

angiosperm relationships, and ‘ending incongruence’: a cautionary tale in phylogenetics. Trends Plant Sci. 2004;9(10):477-83. doi: 10.1016/j.tplants.2004.08.008.

19. Jansen RK, Cai ZQ, Raubeson LA, Daniell H, Depamphilis CW, Leebens-Mack J, et al. Analysis of 81 genes from 64 plastid genomes resolves relationships in angiosperms and identifies genome-scale evolutionary patterns. P Natl Acad Sci USA. 2007;104(49):19369-74. doi: 10.1073/pnas.0709121104.

20. Jin JJ, Yu WB, Yang JB, Song Y, DePamphilis CW, Yi TS, et al. GetOrganelle: a fast and versatile toolkit for accurate de novo assembly of organelle genomes. Genome Biol. 2020;21(1):1-31. doi: 10.1186/s13059-020-02154-5.

21. Dierckxsens N, Mardulyn P, Smits G. NOVOPlasty: de novo assembly of organelle genomes from whole genome data. Nucleic Acids Res. 2017;45(4):e18-e. doi: 10.1093/nar/gkw955.

22. Yang JB, Tang M, Li HT, Zhang ZR, Li DZ. Complete chloroplast genome of the genus *Cymbidium*: lights into the species identification, phylogenetic implications and population genetic analyses. BMC Evol Biol. 2013;13(1):1-12. doi: 10.1186/1471-2148-13-84.

23. Li XW, Yang Y, Henry RJ, Rossetto M, Wang Y, Chen SL. Plant DNA barcoding: from gene to genome. Biol Rev. 2015;90(1):157-66. doi: 10.1111/brv.12104.

24. Tonti- Filippini J, Nevill PG, Dixon K, Small I. What can we do with 1000 plastid genomes? Plant J. 2017;90(4):808-18. doi: 10.1111/tpj.13491.

25. Zhang Z, Zhang DS. The complete chloroplast genome sequence of *Ficus sarmentosa* (Moraceae, Rosales), a widely distributed fig tree in East Asia. Mitochondrial DNA B. 2022;7(9):1597-8. doi: 10.1080/23802359.2022.2115860.

26. Bruun-Lund S, Clement WL, Kjellberg F, Rønsted N. First plastid phylogenomic study reveals

potential cyto-nuclear discordance in the evolutionary history of *Ficus* L. (Moraceae). Mol Phylogenet Evol. 2017;109:93-104. doi: 10.1016/j.ympev.2016.12.031.

27. Roure B, Baurain D, Philippe H. Impact of missing data on phylogenies inferred from empirical phylogenomic data sets. Mol Biol Evol. 2013;30(1):197-214. doi: 10.1093/molbev/mss208.

28. Zhang SD, Jin JJ, Chen SY, Chase MW, Soltis DE, Li HT, et al. Diversification of Rosaceae since the Late Cretaceous based on plastid phylogenomics. New Phytol. 2017;214(3):1355-67. doi: 10.1111/nph.14461.

29. Gao LZ, Liu YL, Zhang D, Li W, Gao J, Liu Y, et al. Evolution of *Oryza* chloroplast genomes promoted adaptation to diverse ecological habitats. Commun Biol. 2019;2(1):1-13. doi: 10.1038/s42003-019-0531-2.

30. Liu ZF, Ma H, Ci XQ, Li L, Song Y, Liu B, et al. Can plastid genome sequencing be used for species identification in Lauraceae? Bot J Linn Soc. 2021;197(1):1-14. doi: 10.1093/botlinnean/boab018.

31. Berg CC. Flora Malesiana precursor for the treatment of Moraceae 4: *Ficus* subgenus *Synoecia*. Blumea. 2003;48(3):551-71. doi: 10.3767/000651903X489546.

32. Corner EJH. Taxonomic notes on *Ficus* L., Asia and Australasia. Gard Bull Singapore. 1960;18(1):1-69.

33. Corner EJH. Check-list of *Ficus* in Asia and Australasia with keys to identification. Gard Bull Singapore. 1965;21:1-186.

34. Chang SS. Three new species of the Moraceae from China. Acta Phytotax Sin. 1982;20(1):95-8.

35. Chang SS. New taxa of Moraceae from China. Guihaia. 1983;3(4):295-306.

36. Chang SS. New Taxa of Moraceae from China and Vietnam. Acta Phytotax Sin. 1984;22(1):64-76.

37. Chang SS, Wu CY, Cao ZY. Moraceae (*Ficus*). In: Chang SS, Wu CY, editors. Flora Reipublicae Popularis Sinicae. 23. Beijing: Science Press; 1998. p. 66-169.

38. Yamazaki T. Taxonomic review of the Moraceae from Japan, Korea, Taiwan and adjacent areas (2). J Phytogeo Taxon. 1983;31(1):1-15.

39. Tsai L, Hayakawa H, Fukuda T, Yokoyama J. A breakdown of obligate mutualism on a small island: An interspecific hybridization between closely related fig species (*Ficus pumila* and *Ficus thunbergii*) in Western Japan. Amer J Pl Sci. 2015;6(01):126-31. doi: 10.4236/ajps.2015.61014.

40. Berg CC. Precursory taxonomic studies on *Ficus* (Moraceae) for the Flora of Thailand. Thai Forest Bull. 2007;35:4-28.

41. Doyle JJ, Doyle JL. A rapid DNA isolation procedure for small quantities of fresh leaf tissue. Phytochem Bull. 1987;19:11-5.

42. Qu XJ, Moore MJ, Li DZ, Yi TS. PGA: a software package for rapid, accurate, and flexible batch annotation of plastomes. Plant Methods. 2019;15(1):1-12. doi: 10.1186/s13007-019-0435-7.

43. Greiner S, Lehwark P, Bock R. Organellar Genome DRAW (OGDRAW) version 1.3.1: expanded toolkit for the graphical visualization of organellar genomes. Nucleic Acids Res. 2019;47(W1):W59-W64. doi: 10.1093/nar/gkz238.

44. Amiryousefi A, Hyvönen J, Poczai P. IRscope: an online program to visualize the junction sites of chloroplast genomes. Bioinformatics. 2018;34(17):3030-1. doi: 10.1093/bioinformatics/bty220.

45. Kurtz S, Choudhuri JV, Ohlebusch E, Schleiermacher C, Stoye J, Giegerich R. REPuter: the manifold applications of repeat analysis on a genomic scale. Nucleic Acids Res. 2001;29(22):4633-42. doi: 10.1093/nar/29.22.4633.

46. Beier S, Thiel T, Münch T, Scholz U, Mascher M. MISA-web: a web server for microsatellite

prediction. Bioinformatics. 2017;33(16):2583-5. doi: 10.1093/bioinformatics/btx198.

47. Frazer KA, Pachter L, Poliakov A, Rubin EM, Dubchak I. VISTA: computational tools for comparative genomics. Nucleic Acids Res. 2004;32(suppl_2):W273-W9. doi: 10.1093/nar/gkh458.

48. Edgar RC. MUSCLE: multiple sequence alignment with high accuracy and high throughput. Nucleic Acids Res. 2004;32(5):1792-7. doi: 10.1093/nar/gkh340.

49. Xia X. DAMBE 6: new tools for microbial genomics, phylogenetics, and molecular evolution. J Hered. 2017;108(4):431-7. doi: 10.1093/jhered/esx033.

50. Chen CJ, Chen H, Zhang Y, Thomas HR, Frank MH, He YH, et al. TBtools: an integrative toolkit developed for interactive analyses of big biological data. Mol Plant. 2020;13(8):1194-202. doi: 10.1016/j.molp.2020.06.009.

51. Huang YY, Li J, Yang ZR, An WL, Xie CZ, Liu SS, et al. Comprehensive analysis of complete chloroplast genome and phylogenetic aspects of ten *Ficus* species. BMC Plant Biol. 2022;22(1):1-15. doi: 10.1186/s12870-022-03643-4.

52. Gardner EM, Garner M, Cowan R, Dodsworth S, Epitawalage N, Arifiani D, et al. Repeated parallel losses of inflexed stamens in Moraceae: phylogenomics and generic revision of the tribe Moreae and the reinstatement of the tribe Olmedieae (Moraceae). Taxon. 2021:946-88. doi: 10.1002/tax.12526.

53. Katoh K, Standley DM. MAFFT multiple sequence alignment software version 7: improvements in performance and usability. Mol Biol Evol. 2013;30(4):772-80. doi: 10.1093/molbev/mst010.

54. Capella-Gutiérrez S, Silla-Martínez JM, Gabaldón T. trimAl: a tool for automated alignment trimming in large-scale phylogenetic analyses. Bioinformatics. 2009;25(15):1972-3. doi:

10.1093/bioinformatics/btp348.
55. Kalyaanamoorthy S, Minh BQ, Wong TK, Von Haeseler A, Jermiin LS. ModelFinder: fast model selection for accurate phylogenetic estimates. Nat Methods. 2017;14(6):587-9. doi: 10.1038/nmeth.4285.
56. Fan WB, Wu Y, Yang J, Shahzad K, Li ZH. Comparative chloroplast genomics of Dipsacales species: insights into sequence variation, adaptive evolution, and phylogenetic relationships. Front Plant Sci. 2018;9:1-13. doi: 10.3389/fpls.2018.00689.
57. Dong WP, Xu C, Cheng T, Zhou SL. Complete chloroplast genome of Sedum sarmentosum and chloroplast genome evolution in Saxifragales. PloS One. 2013;8(10):e77965. doi: 10.1371/journal.pone.0077965.
58. Luo J, Hou BW, Niu ZT, Liu W, Xue QY, Ding XY. Comparative chloroplast genomes of photosynthetic orchids: insights into evolution of the Orchidaceae and development of molecular markers for phylogenetic applications. PloS One. 2014;9(6):e99016. doi: 10.1371/journal.pone.0099016.
59. Gao BM, Yuan L, Tang TL, Hou J, Pan K, Wei N. The complete chloroplast genome sequence of *Alpinia oxyphylla* Miq. and comparison analysis within the Zingiberaceae family. PloS One. 2019;14(6):e0218817. doi: 10.1371/journal.pone.0218817.
60. Yang JB, Yang SX, Li HT, Yang J, Li DZ. Comparative chloroplast genomes of *Camellia* species. PloS One. 2013;8(8):e73053. doi: 10.1371/journal.pone.0073053.
61. Zhao ML, Song Y, Ni J, Yao X, Tan YH, Xu ZF. Comparative chloroplast genomics and phylogenetics of nine *Lindera* species (Lauraceae). Sci Rep. 2018;8(1):1-11. doi: 10.1038/s41598-018-27090-0.

62. Wu Y, Liu F, Yang DG, Li W, Zhou XJ, Pei XY, et al. Comparative chloroplast genomics of *Gossypium* species: insights into repeat sequence variations and phylogeny. Front Plant Sci. 2018;9:376. doi: 10.3389/fpls.2018.00376.

63. Xia X, Peng JY, Yang L, Zhao XL, Duan AA, Wang DW. Comparative Analysis of the Complete Chloroplast Genomes of Eight *Ficus* Species and Insights into the Phylogenetic Relationships of *Ficus*. Life. 2022;12(6):848. doi: 10.3390/life12060848.

64. Palmer JD. Comparative organization of chloroplast genomes. Annu Rev Genet. 1985;19(1):325-54. doi: 10.1146/annurev.ge.19.120185.001545.

65. Sugiura M. The chloroplast genome. Plant Mol Biol. 1992:149-68. doi: 10.1007/978-94-011-2656-4_10.

66. Jansen RK, Ruhlman TA. Plastid genomes of seed plants. In: Bock R, Knoop V, editors. Genomics of Chloroplasts and Mitochondria. Dordrecht: Springer; 2012. p. 103-26.

67. Mower JP, Vickrey TL. Structural diversity among plastid genomes of land plants. Adv Bot Res. 2018;85:263-92. doi: 10.1016/bs.abr.2017.11.013.

68. Wang J, Li CJ, Yan CX, Zhao XB, Shan SH. A comparative analysis of the complete chloroplast genome sequences of four peanut botanical varieties. PeerJ. 2018;6:e5349. doi: 10.7717/peerj.5349.

69. Li XQ, Zuo YJ, Zhu XX, Liao S, Ma JS. Complete chloroplast genomes and comparative analysis of sequences evolution among seven *Aristolochia* (Aristolochiaceae) medicinal species. Int J Mol Sci. 2019;20(5):1045. doi: 10.3390/ijms20051045. PubMed PMID: doi:10.3390/ijms20051045.

70. Guo XX, Qu XJ, Zhang XJ, Fan SJ. Comparative and phylogenetic analysis of complete plastomes among Aristidoideae species (Poaceae). Biology. 2022;11(1):63. doi:

10.3390/biology11010063. PubMed PMID: doi:10.3390/biology11010063.

71. Zhang R, Wang YH, Jin JJ, Stull GW, Bruneau A, Cardoso D, et al. Exploration of plastid phylogenomic conflict yields new insights into the deep relationships of Leguminosae. Syst Biol. 2020;69(4):613-22. doi: 10.1093/sysbio/syaa047.

72. Liu LM, Du XY, Guo C, Li DZ. Resolving robust phylogenetic relationships of core Brassicaceae using genome skimming data. J Syst Evol. 2021;59(3):442-53. doi: 10.1111/jse.12666.

73. Moore MJ, Soltis PS, Bell CD, Burleigh JG, Soltis DE. Phylogenetic analysis of 83 plastid genes further resolves the early diversification of eudicots. P Natl Acad Sci USA. 2010;107(10):4623-8. doi: 10.1073/pnas.0907801107.

74. Gitzendanner MA, Soltis PS, Wong GKS, Ruhfel BR, Soltis DE. Plastid phylogenomic analysis of green plants: a billion years of evolutionary history. Am J Bot. 2018;105(3):291-301. doi: 10.1002/ajb2.1048.

75. Li HT, Luo Y, Gan L, Ma PF, Gao LM, Yang JB, et al. Plastid phylogenomic insights into relationships of all flowering plant families. BMC Biology. 2021;19(1):1-13. doi: 10.1186/s12915-021-01166-2.

76. Dong WP, Liu J, Yu J, Wang L, Zhou SL. Highly variable chloroplast markers for evaluating plant phylogeny at low taxonomic levels and for DNA barcoding. PloS One. 2012;7(4):e35071. doi: 10.1371/journal.pone.0035071.

77. Shaw J, Shafer HL, Leonard OR, Kovach MJ, Schorr M, Morris AB. Chloroplast DNA sequence utility for the lowest phylogenetic and phylogeographic inferences in angiosperms: the tortoise and the hare IV. Am J Bot. 2014;101(11):1987-2004. doi: 10.3732/ajb.1400398.

78. Dong WP, Xu C, Li CH, Sun JH, Zuo YJ, Shi S, et al. *ycf1*, the most promising plastid DNA

barcode of land plants. Sci Rep. 2015;5(1):1-5. doi: 10.1038/srep08348.

79. Amar MH. *ycf1-ndhF* genes, the most promising plastid genomic barcode, sheds light on phylogeny at low taxonomic levels in *Prunus persica*. J Genet Eng Biotechnol. 2020;18(42):1-10. doi: 10.1186/s43141-020-00057-3.

80. Pederneiras LC, Gaglioti AL, Romaniuc-Neto S, Mansano VF. The role of biogeographical barriers and bridges in determining divergent lineages in *Ficus* (Moraceae). Bot J Linn Soc. 2018;187(4):594-613. doi: 10.1093/botlinnean/boy036.

81. Wei ZD, Kobmoo N, Cruaud A, Kjellberg F. Genetic structure and hybridization in the species group of *Ficus auriculata*: can closely related sympatric *Ficus* species retain their genetic identity while sharing pollinators? Mol Ecol. 2014;23(14):3538-50. doi: 10.1111/mec.12825.

82. Zhang LF, Zhang Z, Wang XM, Gao HY, Tian HZ, Li HQ. Molecular phylogeny of the *Ficus auriculata* complex (Moraceae). Phytotaxa. 2018;362(1):039-54. doi: 10.11646/phytotaxa.362.1.3.

83. Zhang Z, Wang XM, Liao S, Tian HZ, Li HQ. Taxonomic treatment of the *Ficus auriculata* complex (Moraceae) and typification of some related names. Phytotaxa. 2019;399(3):203-8. doi: 10.11646/phytotaxa.399.3.4.

84. Wang J, Li Y, Li C, Yan C, Zhao X, Yuan C, et al. Twelve complete chloroplast genomes of wild peanuts: great genetic resources and a better understanding of *Arachis* phylogeny. BMC Plant Biol. 2019;19(1):1-18. doi: 10.1186/s12870-019-2121-3.

85. Jiao YJ, Feng GY, Huang LK, Nie G, Li Z, Peng Y, et al. Complete Chloroplast Genomes of 14 Subspecies of *D. glomerata*: Phylogenetic and Comparative Genomic Analyses. Genes. 2022;13(9):1621. doi: 10.3390/genes13091621.

Supporting information

S1 Table. The collecting information of samples in the study. (xlsx)

S2 Table. Detailed information for the high-throughput sequencing data in the study. (xlsx)

Fig1

Click here to access/download;Figure;Fig1.pdf

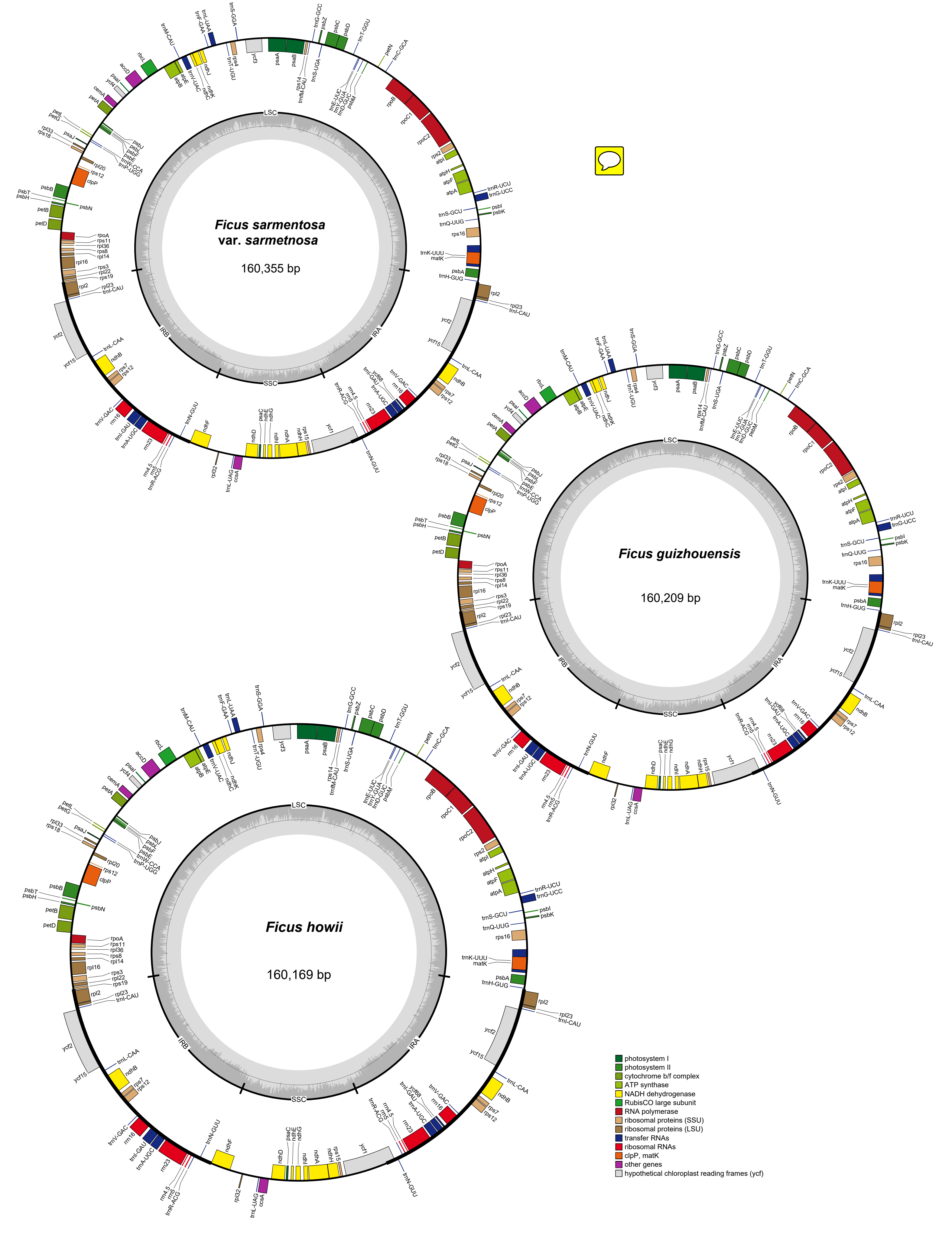

Fig2

Click here to access/download;Figure;Fig2.jpg

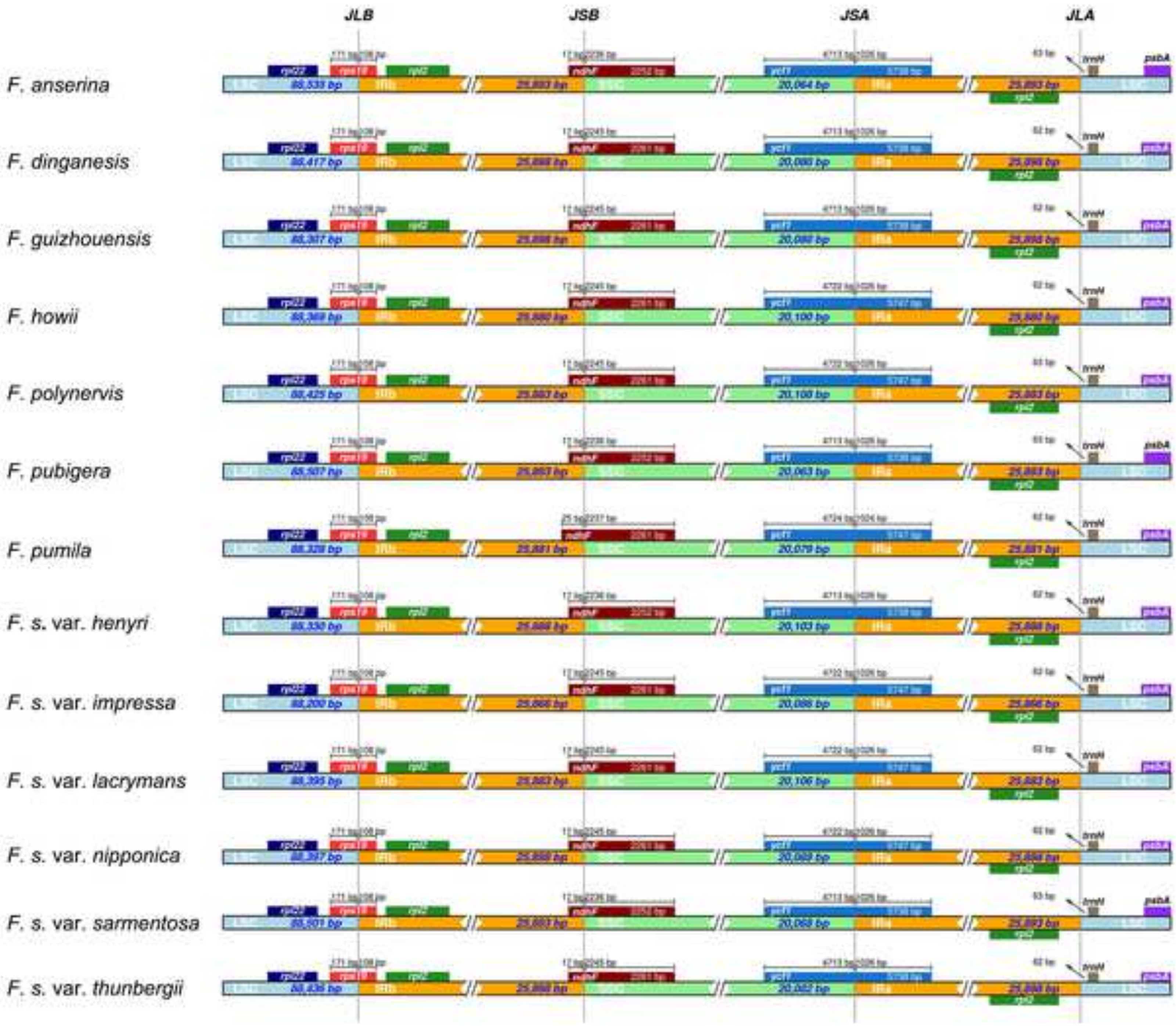

Fig3

Click here to access/download;Figure;Fig3.pdf

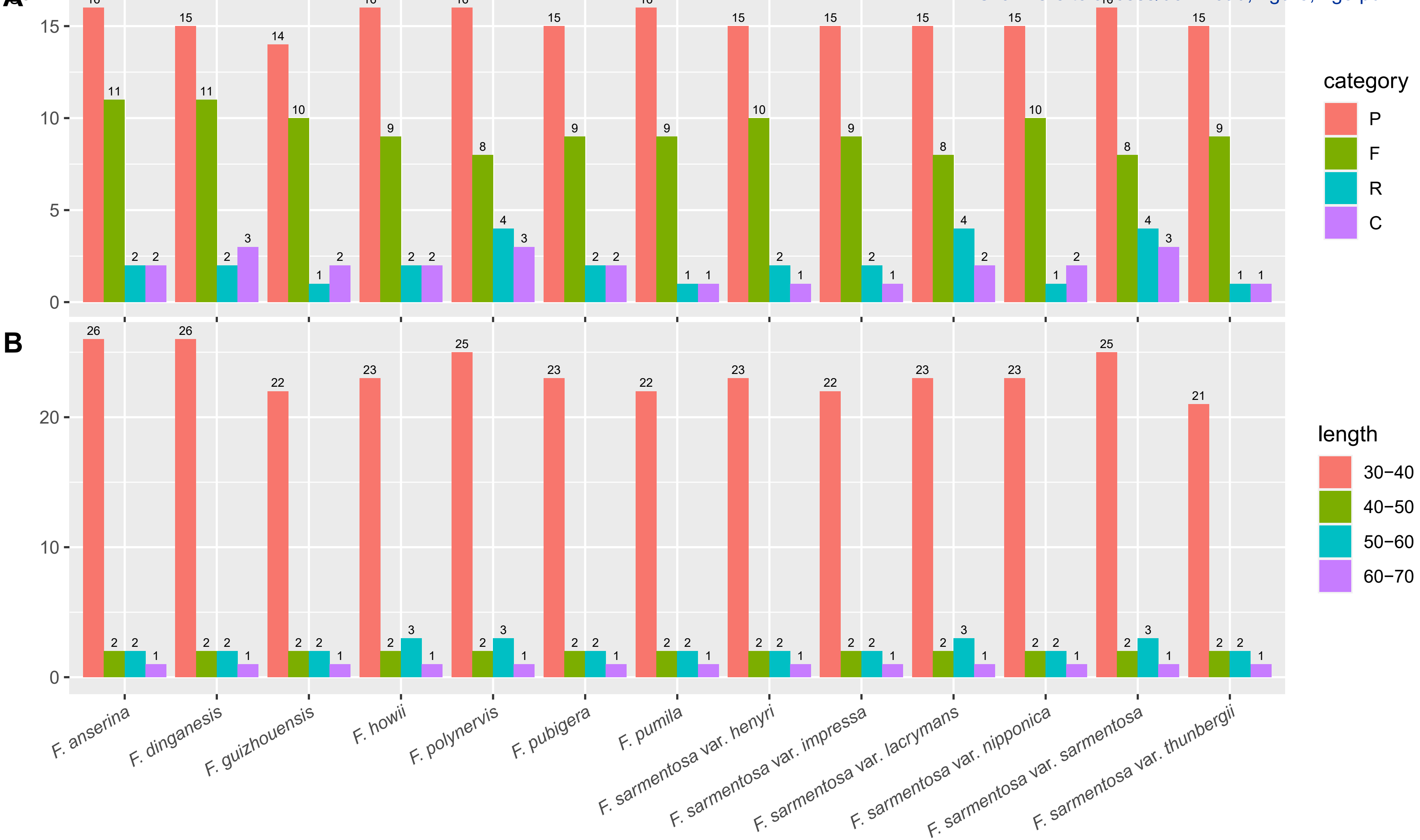

Fig4

Click here to access/download;Figure;Fig4.pdf

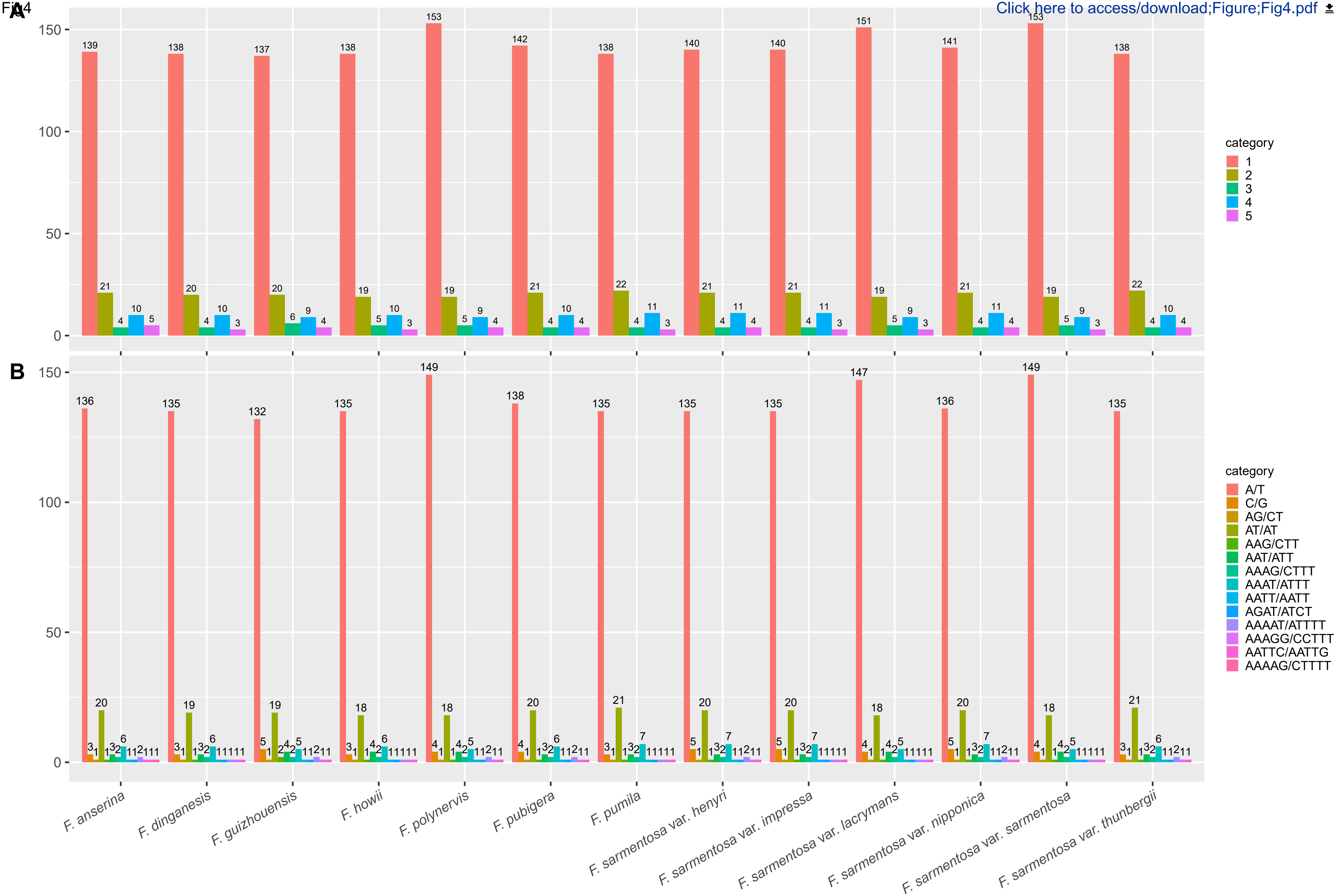

Fig5

Click here to access/download;Figure;Fig5.pdf

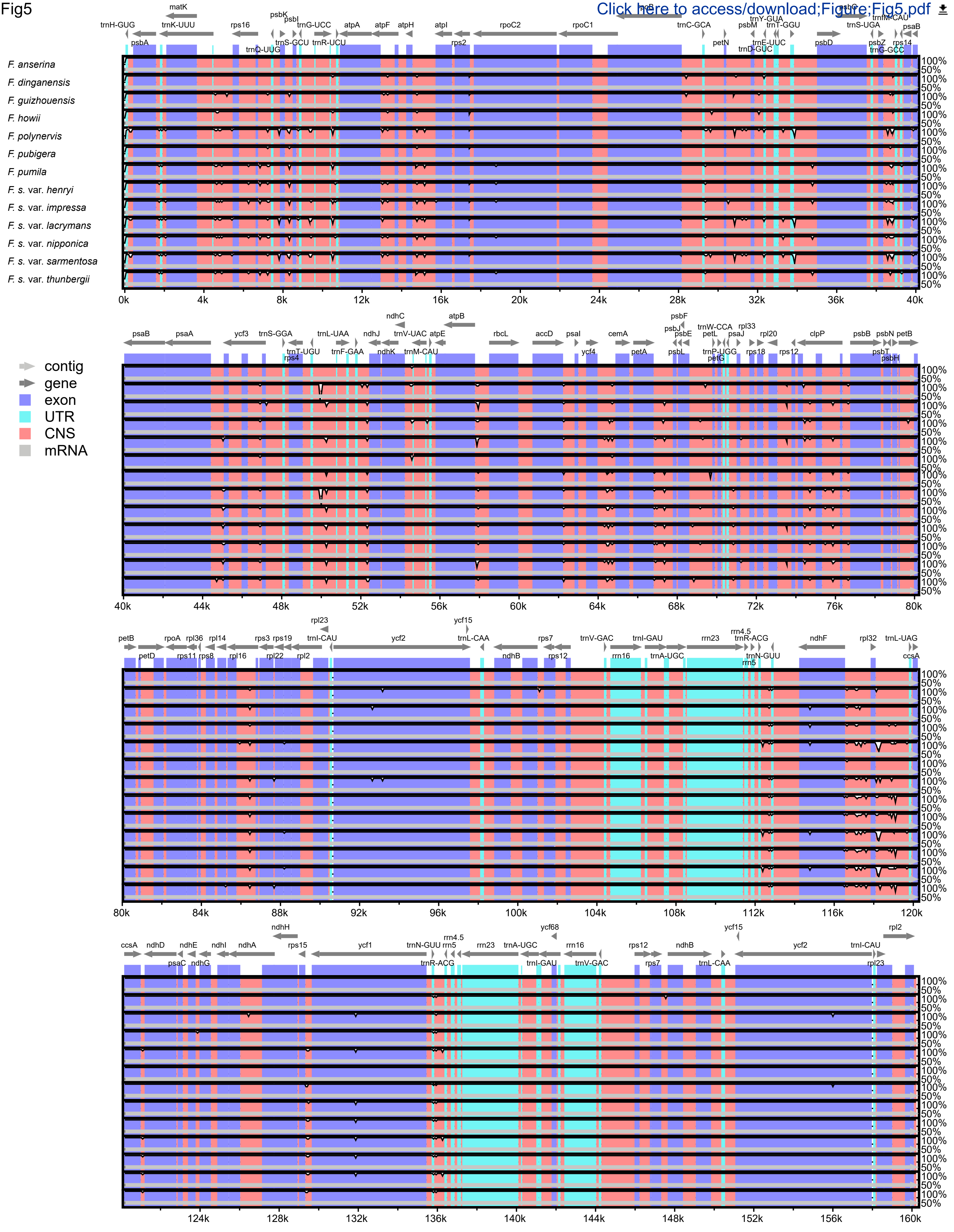

Fig6

Click here to access/download;Figure;Fig6.pdf

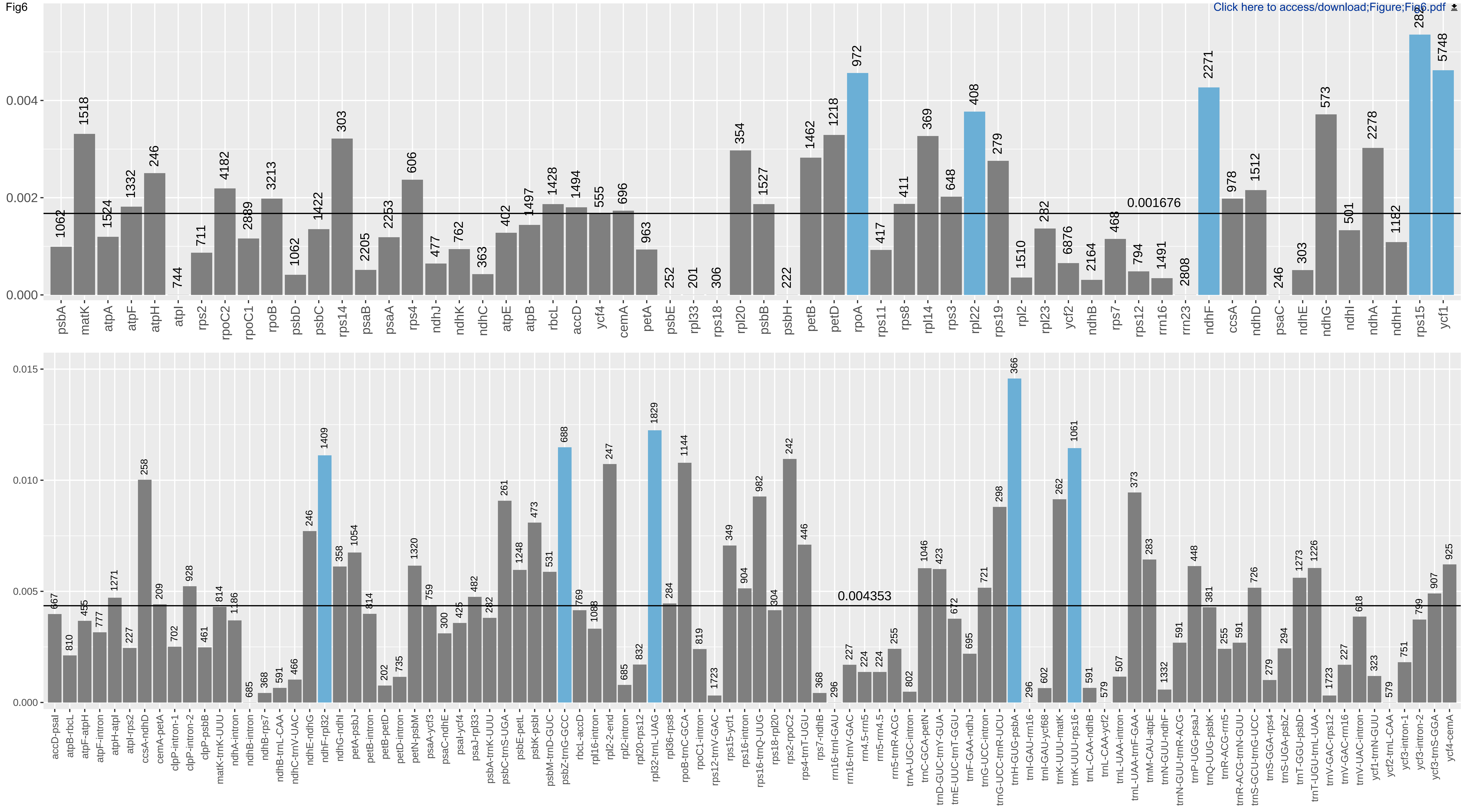

Fig7

Click here to access/download;Figure;Fig7.pdf

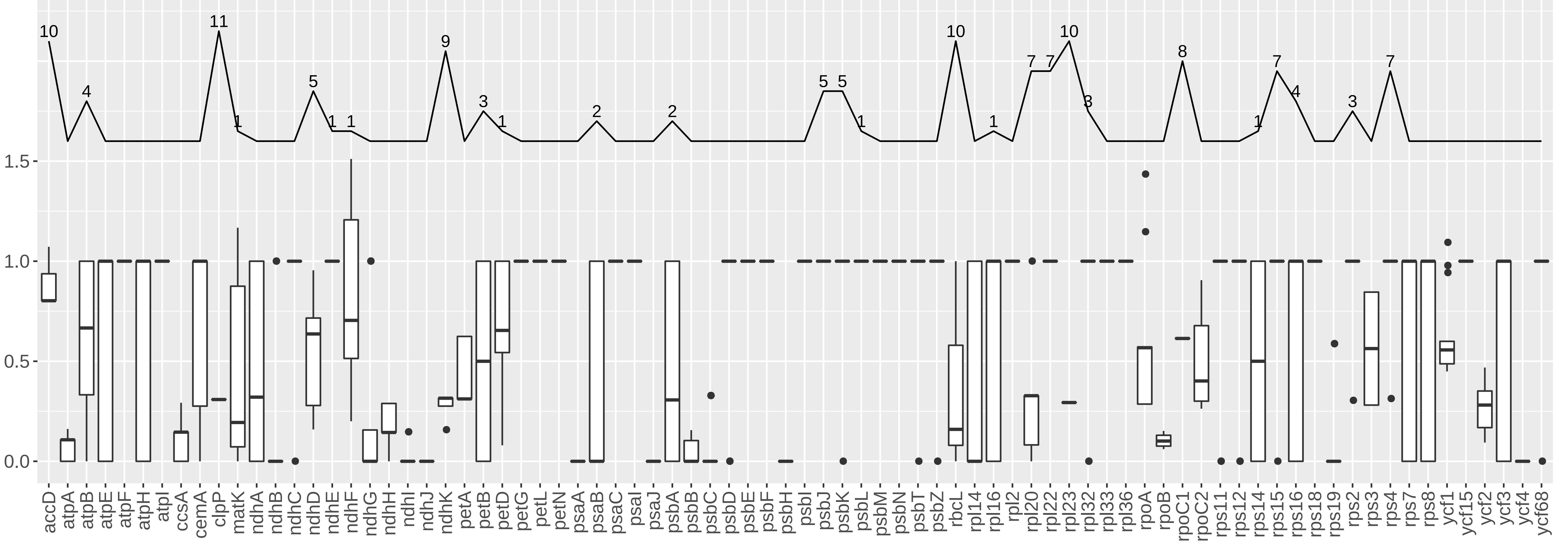

Fig8

Click here to access/download;Figure;Fig8.pdf

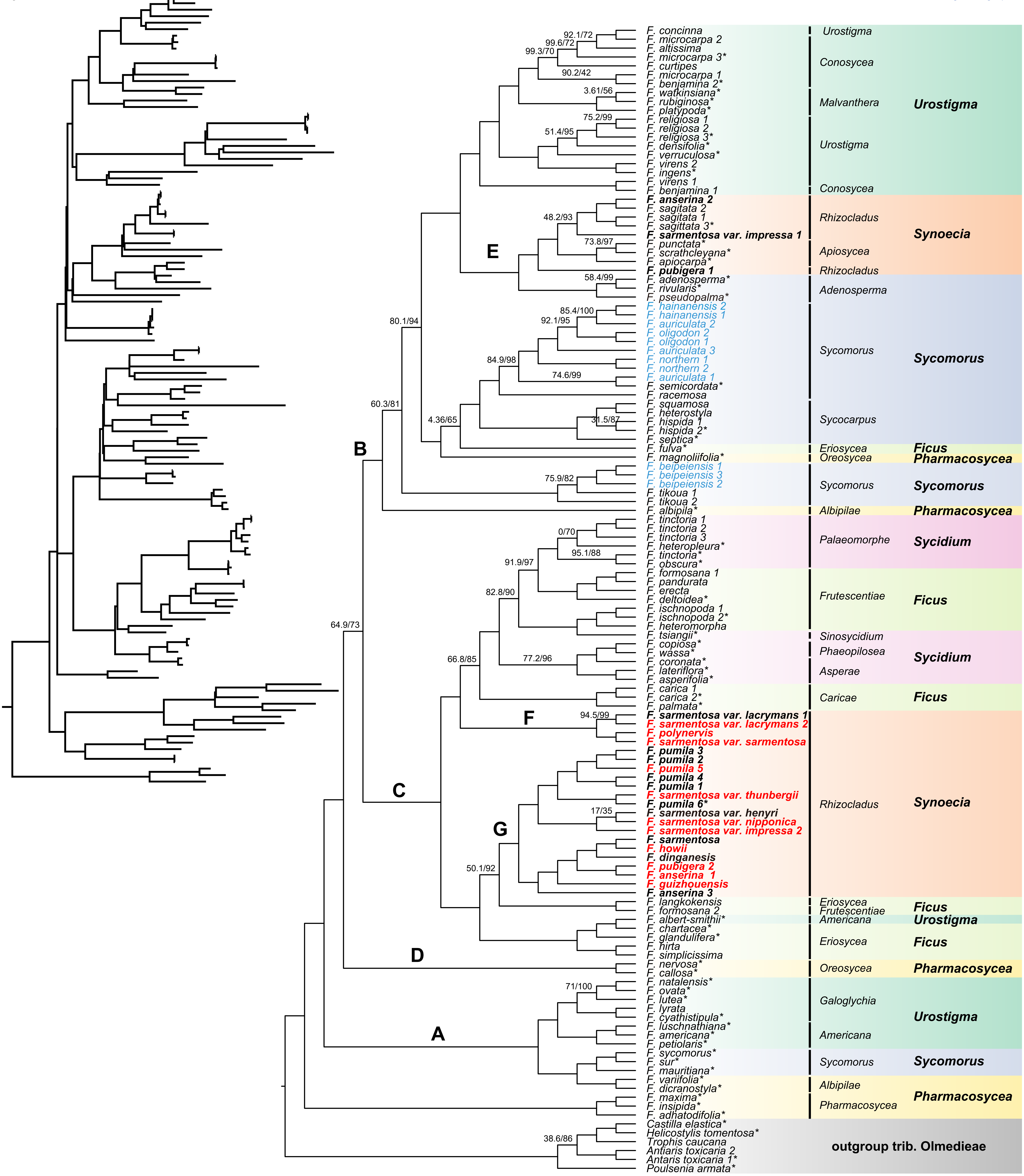

Supporting Information S1 Table

Click here to access/download
Supporting Information
S1_Table.xlsx

Supporting Information S2 Table

Click here to access/download
Supporting Information
S2_Table.xlsx

Revised Manuscript with Track Changes

~~The~~ C~~c~~omparison of chloroplast genomes and phylogenomics in the *Ficus sarmentosa* complex (Moraceae)

Zhen Zhang[1], De-Shun Zhang[1], Zou Lu[2], Chi-Yuan Yao[*1]

[1] College of Architecture and Urban Planning, Tongji University, Shanghai, China

[2] School of Life Sciences, East China Normal University, Shanghai, China

* Corresponding author

E-mail: cyyao@tongji.edu.cn (CYY)

Abstract

~~Owe~~ Due to ~~matrilineal~~ maternal inheritance and ~~low~~ minimal rearrangement, the chloroplast genome is an important genetic resource ~~in~~ for evolutionary ~~research~~studies. However, the evolutionary ~~pattern~~ dynamics and phylogenetic performance of chloroplast genomes in closely related species are poorly characterized, ~~have not drawn deserved attention,~~ particularly in~~especially for the~~ taxonomically ~~complicated~~ complex and species-rich ~~genera~~group. The taxonomically unresolved *Ficus sarmentosa* species complex (~~in~~ Moraceae) comprises ~~about~~ approximately 20 taxa with genetic background~~ically~~ unclear~~ and taxonomically unresolved~~. In this study, we~~To~~ explored the evolutionary ~~pattern~~dynamics, hotspot loci, and phylogenetic performance of thirteen chloroplast genomes ~~in closely related species,~~ ~~thirteen complete chloroplast genomes~~ (including eleven newly obtained and two downloaded from NCBI) representing the *F.~~icus~~ sarmentosa* complex. Their ~~were detailedly compared in the aspects of~~ sequence lengths, IR boundaries~~y~~, repeat sequences, and codon usage were compared. Both ~~S~~sequence length and IR boundaries~~y~~ were found ~~out~~ to be highly~~extremely~~ conserved. All ~~F~~four categories of long repeat sequences ~~exist~~ were found ~~in~~ across all 13 chloroplast genomes, with~~and the~~ palindromic and forward sequences ~~were outright dominant~~being the most common. The ~~SSR detection results~~ ~~showed the~~ number of simple sequence repeat (SSR) loci varied from 175 (*F. dinganensis* and *F. howii*) to 190 (*F. polynervis*), with ~~and~~ the dinucleotide motif ~~was at the highest~~appearing the most frequently~~cy~~. Relative synonymous codon usage (RSCU) analysis indicated that~~The~~ codons ending with A/T were ~~of preference in th~~prior to those ending with C/T~~e relative synonymous codon usage~~. The majority of coding sequence regions were found to have undergone negative selection ~~selective~~ ~~pressure analysis unearthed that the majority of CDS regions underwent the purifying selection except~~ ~~for~~with the exception of ten genes (*accD*, *clpP*, *ndhK*, *rbcL*, *rpl20*, *rpl22*, *rpl23*, *rpoC1*, *rps15*, and

Formatted: Font: Italic

rps4) ~~with~~ which exhibited potential positive selective signatures. Five hypervariable genic regions (*rps15*, *ycf1*, *rpoA*, *ndhF*, and *rpl22*) and five hypervariable intergenic regions (*trnH-GUG-psbA*, *rpl32-trnL-UAG*, *psbZ-trnG-GCC*, *trnK-UUU-rps*16 and *ndhF-rpl32*) were ~~recommended for barcoding and population genetics in related species~~identified. Overall, ~~P~~phylogenomic analysis based on ~~a total of~~ 123 *Ficus* chloroplast genomes showed promise for s~~in the genus *Ficus* showed~~ tudying the evolutionary relationships~~he chloroplast genome is high-resolution~~ in *Ficus*~~.~~, despite cyto-nuclear discordance. ~~but inconsistent with the previous nuclear phylogenetic framework.~~ Furthermore, based on the phylogenetic performance of the *F. sarmentosa* complex and *F. auriculata* complex, the chloroplast genome also ~~revealed~~ exhibited a promising phylogenetic resolution in closely related species.

Introduction

The genus *Ficus* L. (Moraceae) is a species-rich ~~genus~~ taxa which ~~with~~ contains at least 800 species and is –widely distributinged ~~in~~ across tropical and subtropical regions [1-3]. Due to insufficient genetic differentiation, ~~As the largest genus in Moraceae,~~the genus *Ficus* is taxonomically complex ~~there~~ and contains~~are~~ ~~numerous~~ many sympatric species ~~groups that are taxonomically difficult due to the insufficient genetic differentiation in *Ficus*~~, ~~such as~~including the *F. pedunculosa* group, *F. punctata* group, *F. chartacea* group, and *F. subulata* group, ~~and so on, leading the significant taxonomic complexity in *Ficus*~~among others [1, 3, 4]. Meanwhile, ~~currently confirmed~~ widely not-strict one-to-one obligate mutualism between fig trees and fig wasps has r~~r~~esults in ~~the~~ frequent hybridization and introgression among *Ficus* species [5-8], which have so far hinders~~ed~~ ~~the further~~ research on~~of~~ ~~the~~ the taxonomy and evolutionary history ~~genus in taxonomy and evolution~~ [9]. Our current understanding of ~~T~~the *Ficus* ~~present~~ phylogenetic framework ~~of *Ficus*~~ is the result of research

Formatted: Font: Italic

Formatted: Font: Not Italic

Formatted: Font: Italic

Formatted: Font: Italic

Formatted: Font: Italic

on~~ed from~~ a few nuclear loci, such as ITS, ETS, *G3pdh*, GBSSI, and *waxy* [3, 4, 10-14]. Al~~T~~though nuclear genome~~ic~~ data have been~~are beginning to~~ used to reconstruct the *Ficus* phylogeny ~~of *Ficus* in the latest research~~ [15-17], these studies represent less than ten percent of *Ficus* species, which is unlikely to accurately represent the ~~seems insufficient to qualify~~ evolutionary relationship in the genus *Ficus*.

While the use of ~~The~~ nuclear genome~~ic~~ data has~~ve shown unique~~ advantages ~~in~~ for the detection of~~ing~~ hybridization and introgression, ~~. Whereas~~ organellar~~e~~ genomic~~etic~~ resources are also of great importance to ~~also plays an important role in~~ evolutionary research ~~owing~~ due to ~~matrilineal~~ maternal inheritance ~~and evolution~~ as a single unit [18, 19]. In the last decade, due to the rapidly decreasing cost of whole-genome sequencing ~~WGS~~ (WGS~~whole genome sequencing~~) and the development of chloroplast genomic ~~resembling~~ assembling pipelines, such as GerOrganelle [20], Fast-Plast (https://github.com/mrmckain/Fast-Plast), NOVOPlasty [21], and ORG.asm (https://git.metabarcoding.org/org-asm/org-asm), ~~the acquirement of the chloroplast genome~~ plastome-based evolutionary research have become ~~is increasingly~~ easier and more cost-effective~~, which extremely promotes the application of plastomes in evolutionary research~~ [22-24]. However, chloroplast genomes ~~of less than five percent of species in *Ficus*~~ are publicly available for less than five percent of species in *Ficus* ~~in the public data repository GenBank for the genus *Ficus*~~ [25]. Bruun-Lund et al. [26] ~~innovatively proposed~~published a novel and innovative *Ficus* phylogenetic framework ~~within *Ficus*~~ based on 59 newly obtained chloroplast genomes, and the results of which ~~indicated~~ were obvious inconsistent~~cy~~ with phylogenies based on the nuclear ~~phylogenetic results~~genome. ~~Regrettably~~Unfortunately, many of the ~~genomic~~ genome sequences used by~~of~~ Bruun-Lund et al. ~~are with~~contained gaps (an average of 7% missing data ~~and~~ with a maximum of 65%), ~~which leads~~ leading

Formatted: Font: Italic

to the difficulty of in comparative chloroplast genomics and possible phylogenetic artifacts [27]. Even so, the chloroplast framework of Brunn-Lund et al. have been replicated by the subsequent research generally reappeared the chloroplast framework of Brunn-Lund et al. basedwith on an extended dataset [17]. On the wholeOverall, based onaccording to the studies on in both *Ficus* and the other taxa [28-30], chloroplast genomes it hasve been verified to be effective for the chloroplast genomes to reconstrcut the phylogenetic reference in the ranks ofat infra-, and inter-generic, and andthe higher onesranks.

However, little is known about the phylogenetic performance of the complete chloroplast genomes in terms studies of closelythe related species or even infra-species, especially particularly in the taxonomically complex genera such asus *Ficus*.–

The subsect. *Plagiostigma* in *Ficus* subg. *Synoecia* sect. *Rhizocladus* subsect. *Plagiostigma* comprises about approximately 10 species and 11 varieties which are widely distributed in across eEast Asia, parapatric to the distribution center of *Ficus*, sSoutheast Asia [1, 3, 13, 16, 31]. The species of the sSubsect. *Plagiostigma* is genetically unclear and taxonomically unresolved, forming a species complex called the *Ficus sarmentosa* species complex here [4]. After In the *F. sarmentosa* complex, since tthe first systematic treatment of the, *F. sarmentosa* complex by Corner in 1960 and 1965 [32, 33], few studies have attempted to unravel the tried to deal with its taxonomical puzzlescomplexity of this group with the exception of except that some currently descriptions of several controversial taxa taxa were descripted in 1980s [1, 34-37] (such as *F. dinganensis*, *F. guizhouensis*, and *F. polynervis*) and the rank elevation of some varieties (such as *F. pubigera* var. *anserina* and *F. sarmentosa* var. *thunbergii*) were considered the species rank [38-40]. More recently, some of Tthisese phylogenetic ambiguityissues wasere partly discovered and concluded by resolved through the molecular work of

Formatted: Font: Italic

Zhang et al. [4]. Zhang's work resulted in~~, such as~~ the rank elevation of *F. pubigera* var. *anserina*~~.~~ and the discovery ~~Furthermore, the work also showed~~ that *F. sarmentosa* is not monophyletic, ~~and has a~~ complicating the~~ed~~ relationship between the complex and ~~with~~ these ~~newly~~ previously described species in ~~the~~ 1980s. However, because ~~Considering~~ only three loci (ITS, ETS, and *G3pdh*) were used to ~~explore~~ resolve the genetic background ~~in~~ of the complex, ~~and~~ the results were neither stable nor high-resolution [4~~], ~~]. The inclusion of more variable genetic loci or genom~~e~~i~~c~~ data should be ~~indispensable~~ helpful to ~~discover~~ resolve the ~~evolutionary~~ taxonomic ~~research~~ uncertainty of~~in~~ the *F. sarmentosa* complex. ~~Hence~~In this study, we utilized both ~~based on the~~ comparative chloroplast genomics and phylogenomics ~~in the *F. sarmentosa* complex, we aim~~ to ~~obtain~~ characterize~~further information on~~ 1) the diversity of hotspot loci, 2) the variation ~~pattern of~~ among chloroplast genomes, and 3) the ~~phylogenetic~~ potential of chloroplast genomes to resolve the evolutionary relationships between ~~at~~ closely related species of the F. sarmentosa species complex.

Materials and methods

Sample collecti~~o~~on~~ng~~, DNA extraction and resequencing, and genome assembly~~,~~ and annotation

Healthy, ~~and~~ young leaves were ~~sampled~~ collected from the field in 2015-2021~~,1~~ and each ~~then~~was sealed in silica gel. Based on the phylogenetic relationships outlined in Zhang et al. [4], we sampled ~~A total of~~ eleven taxa within the *F. sarmentosa* species complex ~~were sampled based on the main clades in the phylogenetic tree from Zhang et al. [4],~~ in order to ~~aiming to~~ ~~maxim~~maximize~~ally cover its~~ genetic coverage~~diversity~~. ~~The d~~Detailed ~~collecting~~ sample information ~~of the samples was~~is shown in S1 Table. All~~The~~ voucher specimens were stored ~~in~~ at the herbarium of East China Normal University (HSNU).

Formatted: Font: Italic

T~~he t~~otal DNA was extracted ~~form~~ from 100 mg of dry leaf tissue ~~used by~~using the CTAB method [41]. After quality detection ~~by~~ with NanoDrop and Qubit 2.0, ~~the~~ purified DNA samples were randomly ultrasonicated into ~~lly randomly segmented to~~ ~350 bp segments, which were subsequently ~~and then~~ used to construct paired-end libraries. Whole-~~-~~genome resequencing (WGS) was ~~implemented~~ carried out using~~in~~ the Illumina NovaSeq 6000 platform, according to the ~~with~~ PE150 sequencing strategy. ~~The r~~Raw reads were filtered and cleaned according to the following ~~the~~ criteria~~—~~: reads ~~with over~~containing > 10% unidentified nucleotides, ~~more than~~> 50% ~~of~~ low-quality bases (Q≤5), or adapter sequences were omitted for further analyses. ~~Meanwhile~~Finally, the chloroplast genom~~e~~e sequences of ~~another~~ two more taxa in the *F. sarmentosa* complex, *F. sarmentosa* var. *henryi* (GenBank accession no. OL415083) and *F. dinganensis* (GenBank accession no. OK375500), were ~~added~~ included for further~~ollowed~~ comparative analyses.

Clean data were ~~GetOrganelle v1.6.4 [20] was~~ used to assemble the complete circular chloroplast genome with GetOrganelle v1.6.4 [20], utilizing ~~using all the clean data under~~ the "embplant_pt" model. In the case that ~~If~~ the output sequence was~~is~~ not circular, the ~~parameters~~ R (number of runs) and w (word size) parameters were~~are~~ adjusted until ~~to be~~ circularity was achieved. ~~The~~ Chloroplast genome annotation ~~of chloroplast genomes~~ was ~~implemented~~ carried out ~~by~~ using PGA [42], with ~~under~~ the default parameters. The chloroplast genome was visualized~~ation~~ using ~~of the chloroplast genome was accomplished on~~ the online ~~website~~ OGDRAW tool (https://chlorobox.mpimp-golm.mpg.de/OGDraw.html) [43]. For consistency, the ~~T~~two ~~added~~ supplementary plastome sequences (OL415083 and~~,~~ OK375500) were re-annotated ~~under the same parameters for consistent results~~according to the same routine.

Analysis of c~~C~~hloroplast genome structure ~~analyses~~

Aall 13 *F. sarmentosa* complex genomes were analyzed to determine the in the *F. sarmentosa* complex.The lengths and GC contents of the whole genomes, , four quadripartite regions ((large single copy (LSC), small single copy (SSC), and two inverted repeats (IRs))), and coding sequence (CDS)s regions (CDS). were surveyed for all 13 genomes in the *F. sarmentosa* complex.

Analysis of IR contraction and expansion analysis

To examine the boundary variation of LSC/IR/SSC of 13 genomes, the online R Sshinny application IRscope (https://irscope.shinyapps.io/irapp/) [44] was used to examine and visualize the boundary variation of LSC/IR/SSC of all 13 *F. sarmentosa* complex genomes..

Analysis of long repeat sequences and SSRs

The four categories of longer repeat sequences, forward (F), reverse (R), complement (C), and palindromic (P), were analyzed using the REPuter online tools (https://bibiserv.cebitec.uni-bielefeld.de/reputer, University of Bielefeld, Bielefeld, Germany) [45], with 50 maximum computed repeats set as 50 and a minimal repeat size set asof 8. One of the inverted repeat regions (IRb) was removed in the REPuter analysis to avoid repeatable results. Simple sequences repeats (SSRs) were detected via using MISA (https://webblast.ipk-gatersleben.de/misa/) [46], with under the following parameters: 8 repeat units for mono-nucleotide SSRs, 5 repeat units for dinucleotide SSRs, 4 repeat units for trinucleotide SSRs, and 3 repeat units for tetra-, penta-, and hexa-nucleotide SSRs. The Mmaximal sequence length of sequences between two SSRs was set toas 100 bp.–

The cComparison of whole complete chloroplast genomes and diversity hotspot analysis

All thirteen 13 *F. sarmentosa* complex genomes were overall compared via using the mVISTA online tool (https://genome.lbl.gov/vista/mvista/submit.shtml) [47], with in the gGlobal multiple

alignment model (LAGAN). The *F. anserine* chloroplast genome ~~of *F. anserine*~~ was ~~chosen~~ used as the reference and the RankVISTA probability threshold was set to~~as~~ 0.5. All ~~the~~ genes and intergenic regions were extracted from the Genbank annotation files ~~of all 13 genomes~~ in batches using ~~y~~ Perl scripts ~~from~~ created by ~~Qu~~ Xiao-Jian Qu (https://github.com/quxiaojian/Bioinformatic_Scripts). A~~nd then, respective a~~lignments of ~~all~~ the genic and intergenic loci ~~from all 13 genomes~~ were ~~finished~~ carried out using~~by~~ MUSCLE v5.1 [48], with ~~the~~ default parameters. ~~Based on the alignment loci~~After alignment, the nucleotide diversity (π~~Pi~~) was calculated ~~one by one~~ for all ~~the~~ genic and intergenic loci.

Codon usage analysis

To compare ~~C~~codon usage patterns of ~~analysis was explored for~~ all the CDS sequences across 13 *F. sarmentosa* complex genomes~~,~~. ~~To examine the relative preference of codon usage encoding the same amino acid,~~ the relative~~ly~~ synonymous codon usage (RSCU) was calculated using DAMBE v7.3.11 [49].

Formatted: Font: Italic

Selective pressure analysis

The ratio of nonsynonymous (Ka) to synonymous (Ks) substitution can be used to quantify ~~the~~ evolutionary selective pressure. ~~For all the 79 unique CDS gene sequences, t~~The ~~ratio~~ Ka/Ks ratio was calculated for all 79 unique CDSs ~~by~~using TBtools v1.09876 [50]. ~~When Ka/Ks > 1, it indicated a p~~Positive selection ~~effect~~was indicated by Ka/Ks > 1, negative (purifying) selection was indicated by~~;~~ Ka/Ks < 1, ~~purification selection effect;~~ and neutral selection was indicated by Ka/Ks = 1~~, neutral evolution~~. Based on the phylogenetic framework outlined in Zhang et al. [4], ~~*Ficus*~~ *F. simplicissima* ~~, a related species to the *F. sarmentosa* complex based on Zhang et al. [4],~~ was chosen as the reference to calculate Ka and Ks between *F. simplicissima* and our sampled representatives~~s in~~ of the *F. sarmentosa* complex. For visualization purposes, the NaN value (~~,~~i.e., both Ks and Ka ~~are zero~~ = 0) ~~,~~was manually

set as “1”~~one~~ ~~for the convenience of visualization, considering that the value one means~~to denote neutral ~~evolution~~selection. ~~Furthermore~~Finally, the infinity value (Ks > 0 and Ka = 0) was counted alone.

Phylogenetic analysis

The 13~~irteen~~ *F. sarmentosa* complex chloroplast genomes were combined with~~in the F. sarmentosa complex,~~ 36 ~~available~~ *Ficus* chloroplast genomes ~~in~~ from GenBank~~,~~ and 59 *Ficus* genomes ~~from the work o~~published by ~~f~~ Bruun-Lund et al. [26] ~~were chosen~~. ~~Furthermore~~Additionally, ~~eighteen~~ 18 genomes from the China National GeneBank (accession number: CNP0001337) and Genome Sequence Archive (accession number: PRJCA002187) [15, 51], including ~~eight~~ 8 samples belonging to the *F. sarmentosa* complex, were assembled to further explore the *Ficus* phylogenomics ~~of Ficus~~ and ~~phylogenetic~~ the potential of chloroplast genomes to resolve the evolutionary relationships ~~in~~ among the closely related species. Seven samples from~~of~~ the ~~tribe~~ Olmedieae tribe were chosen as the outgroup, according to ~~based on~~ the previous ~~works~~ studies [26, 52]. After discarding four samples found to be more than half ~~with more than~~ (> 50%) missing data (three in the genus *Ficus* and one in the outgroup), ~~–~~a total of 129 chloroplast genomes were used to construct the phylogenetic tree (detailed samples information shown in S1 Table). Additionally, ~~O~~one of the two ~~inverted regions~~IRs was removed. The 129 genomes were aligned using MAFFT v7.490 [53], with ~~was implemented to align the 129 genomes under~~ the “auto” model. The aligned sequences were trimmed ~~via~~ with trimAl ~~(~~and sites with ~~more than~~ > 10% gaps were removed~~)~~ [54]. The maximum likelihood (ML) tree was ~~implemented~~ constructed ~~by~~ using IQ-TREE 2 with ultrafast bootstrap (-bb) and ~~aLRT~~aLRT test (-alrt) numbers set at~~as~~ 10000. The optimal nucleotide substitution~~e~~ model was chosen ~~via~~ with ModelFinder [55].

Results

~~The s~~Summary of all 13 complete chloroplast genomes in the *F. sarmentosa* complex

For the eleven newly resequenced~~ing~~ samples, a total of ~~about~~ 79G bases were obtained ~~with~~ from 19,379,830 (*F. sarmentosa* var. *sarmentosa*) to 23,945,593 (*F. sarmentosa* var. *henryi*) clean paired-end reads. The ratios of chloroplast paired reads ~~from chloroplast~~ to ~~the~~ whole reads ranged from 0.87% (*F. sarmentosa* var. *henryi*) to 4.05% (*F. guizhouensis*). The average kmer-coverage values ~~were~~ ranged from 41.5 ~~(~~(*F. sarmentosa* var. *henryi*) to 128.2 (*F. sarmentosa* var. *thunbergii* and *F. pubigera*). Detailed information on high-throughput sequencing data can be found in ~~showed in~~ S2 Table.

All ~~the~~*F. sarmentosa* complex genomes, ~~of 13 fig trees in the *F. sarmentosa* complex (~~including 11 newly obtained genomes and two genomes downloaded from GenBank, ~~) showed~~exhibited a typical quadripartite structure (Fig 1), ~~including~~ containing one ~~large single copy~~LSC, one ~~small single copy~~SSC, and two ~~inverted repeats~~IRs. The lengths of the complete chloroplast genomes ranged from 160,018 (*F. dinganensis*) to 160,385 bp (*F. sarmentosa* var. *lacrymans*) (Table 1). The LSCs ~~of plastome~~ accounted for ~~about~~ 55.12-55.20-% of the total genome size and ranged from 88,200 (*F. dinganensis*) to 88,535 bp (*F. sarmentosa* var. *lacrymans*) in length.~~;~~ T~~t~~he SSCs accounted for ~~about~~ 12.51-12.55-% of the ~~whole~~ total genome size and ranged~~ing~~ from 20,064 (*F. sarmentosa* var. *lacrymans*) to 20,108 bp (*F. pubigera*) in length. ~~In addition, t~~The IRs ~~region of plastome~~ accounted for 16.14-16.17% of the total genome size and varied from 25,866 (*F. dinganensis*) to 25,898 bp (*F. sarmentosa* var. *nipponica* and *F. sarmentosa* var. *thunbergii*). The CDS regions ranged ~~accumulated length of CDS regions ranged~~ from 79,149 (*F. dinganensis*) to 79,308 bp (*F. sarmentosa* var. *impressa*)

in accumulated lengths. The overall GC content of the whole genome ranged from 35.94 to 35.99 %, within which *F. sarmentosa* var. *henryi* was having the highest one GC content, andbut *F. anserine* and *F. pubigera* were having the lowest. The GC contents of LSCs, SSCs, and IRs were also calculated. Overall, and , the IRs regions had the highest average GC content, which –rangeding from 42.63% (*F. anserina*, *F. howii*, and *F. pubigera*) to 42.65% (*F. pumila*, *F. sarmentosa* var. *lacrymans*, and *F. sarmentosa* var. *sarmentosa*). The GC content of SSCs had is the lowest GC content, which east rangeding from 28.94% (*F. sarmentosa* var. *lacrymans* and, *F. sarmentosa* var. *sarmentosa*) to 29.09% (*F. sarmentosa* var. *henryi*, *F. sarmentosa* var. *impressa*, and *F. sarmentosa* var. *nipponica*). The same to all 13 genomes, –A total of 131 genes were annotated for all the genomes equally, including 86 coding genes, 37 transfer RNA (tRNA) genes, and 8 ribosomal RNA (rRNA) genes. Finally, it was discovered that –genes withthe *infA* gene was intensively pseudogenized [17].

Fig 1. Chloroplast gGene maps of chloroplast genomes of *Ficus sarmentosa* var. *sarmentosa*, *Ficus guizhouensis*, and *Ficus howii*. Genes drawn inside are transcribed clockwise and genes drawn outside ones are counterclockwise. Genes belonging toof different functional groups are color coded. In the inner circle, The dark gray and light gray in the inner circle indicate the GC content and AT content, respectively. The boundaries of the large single copy (LSC), small single copy (SSC), and two inverted regions (IRa, IRb) were are also shown in the inner circle.

Table 1. Summary of complete chloroplast genomes of all thirteen taxa in the *F. sarmentosa* species complex.

Formatted: Font: Not Italic

Taxon	Total Length (bp)	LSC Length (bp)~~of LSC~~	SLC Length (bp)~~Length of SLC~~	IR Length ~~of~~ (bp)~~IR~~	CDS Length ~~of~~ (bp)~~CDS~~	Total GC (%)	LSC GC (%)~~of LSC~~	SSC GC (%)~~of SSC~~	IR GC (%)~~of IR~~
F. anserina	160267	88395	20106	25883	79173	35.94	33.61	28.97	42.63
F. dinganensis	160018	88200	20086	25866	79149	35.98	33.67	29.00	42.64
F. guizhouensis	160209	88330	20103	25888	79272	35.98	33.66	29.02	42.64
F. howii	160169	88328	20076	25882	79167	35.98	33.65	29.03	42.63
F. polynervis	160356	88507	20063	25893	79155	35.95	33.62	28.95	42.64
F. pubigera	160299	88425	20108	25883	79167	35.94	33.61	28.98	42.63
F. pumila	160229	88369	20100	25880	79152	35.98	33.64	29.08	42.65
F. sarmentosa* var. *henryi	160183	88307	20080	25898	79170	35.99	33.66	29.09	42.64
F. sarmentosa* var. *impressa	160293	88417	20080	25898	79308	35.97	33.63	29.09	42.64
F. sarmentosa* var. *lacrymans	160385	88535	20064	25893	79155	35.95	33.63	28.94	42.65
F. sarmentosa* var. *nipponica	160314	88436	20082	25898	79170	35.97	33.63	29.09	42.64
F. sarmentosa* var. *sarmentosa	160355	88501	20068	25893	79155	35.95	33.62	28.94	42.65
F. sarmentosa* var. *thunbergii	160282	88397	20089	25898	79179	35.98	33.65	29.06	42.64

IR contraction and expansion

Across all 13 *F. sarmentosa* complex genomes, f~~F~~our junctions among IRs, LSC, and SSC regions ~~for all 13 genomes in the *F. sarmentosa* complex~~ were ~~detailedly~~ compared ~~and shown in~~ (Fig 2). Invariably and consistently across all 13 genomes, ~~The result indicated that~~ the boundary~~ies~~ between the LSC ~~region~~ and IRb regions (JLB) ~~were~~ was located within the ~~gene~~ *rps19* gene, ~~with~~ 171 bp away from its starting base and 108 bp away from its ending base. ~~The result was invariant and consistent in~~

Formatted: Line spacing: single
Formatted: Line spacing: single
Formatted: Line spacing: single
Formatted: Line spacing: single
Formatted: Line spacing: single
Formatted: Line spacing: single
Formatted: Justified, Line spacing: single
Formatted Table
Formatted: Line spacing: single, Widow/Orphan control
Formatted: Line spacing: single, Widow/Orphan control
Formatted: Line spacing: single, Widow/Orphan control
Formatted: Line spacing: single, Widow/Orphan control
Formatted: Line spacing: single, Widow/Orphan control
Formatted: Line spacing: single, Widow/Orphan control

~~all 13 genomes.~~ The boundary between ~~rders between~~the IRb and SSC regions (JSB) was~~ere~~ ~~set~~ located within the ~~gene~~ *ndhF* gene, either~~with~~ 17 or 25 bp (~~only~~ *F. pumila*) away from its starting base and 2,236 ~~bp~~ (*F. anserina*, *F. pubigera*, *F. sarmentosa* var. *henryi*, and *F. sarmentosa* var. *sarmentosa*), 2,237 ~~bp~~ (*F. pumila*), or 2,245 bp (~~the remain of~~all other taxa) away from its ending base~~, respectively~~. The boundary~~rders of~~ between the SSC and IRa regions (JSA) was~~ere~~ located within the ~~gene~~ *ycf1* generegion, either ~~with~~ 4,713 ~~bp~~, 4,722 ~~bp~~, or 4,724 bp away from ~~the~~ its starting base ~~of *ycf1*~~ and~~as well as~~ 1,024 ~~bp~~ (~~only~~ *F. pumila*) or 1,026 bp away from its ending base. For the IRa and LSC regions, ~~their~~ the junctions between them ~~were~~was located between the ~~genes~~ *rpl2* and *trnH* genes, either~~with~~ 62 or 63 bp away from the starting base of the ~~gene~~ *trnH* gene.

Fig 2. Comparison of the boundaries between the large single copy (LSC), small single copy (SSC), and two inverted repeat ~~–~~regions (IRs) among 13 chloroplast genomes in the *F. sarmentosa* complex. The ~~N~~numbers around the vertical lines ~~are~~ indicate the distances between the boundaries and the starting or ending bases of their nearest genes.

Long repeat sequences and simple sequence repeats (SSRs)

Across all 13 *F. sarmentosa* complex genomes, ~~A~~ a total of 373 long repeat sequences were identified, ~~in all 13 genomes~~ ~~including~~ representing all four repeat categories:~~,~~ forward (F), reverse (R), complement (C), and palindromic (P) (Fig 3A)~~. The numbers of four categories for the thirteen genomes were shown in Fig 3 A~~. All four categories of repeats were detected ~~in~~ within all 13 chloroplast genomes. Four taxa, *F. anserina*, *F. dinganensis*, *F. polynervis*, and *F. sarmentosa* var. *sarmentosa*~~.~~, contained~~had~~ the ~~highest~~ greatest numbers of long repeat sequences (~~at~~ 31), while ~~those of~~

Formatted: Font: Italic

F. sarmentosa var. *thunbergii* contained~~was~~ the least (~~at~~ 26). Among all~~In~~ four repeats, ~~the palindromic~~ P repeats were the most common across~~in~~ all 13 genomes, ~~and~~ rang~~ing~~ed from 14 (~~only~~ *F. guizhouensis*) to 16. There were relatively fewer~~The~~ R~~reverse~~ and C~~complement~~ repeats, with ~~were relatively less and up to four in~~ *F. polynervis*, *F. sarmentosa* var. *lacrymans*, and *F. sarmentosa* var. *sarmentosa* containing the most (4). For the lengths of long repeat sequences, 30-39 bp is the most common ranging from 21 ~~times~~ (*F. sarmentosa* var. *thunbergii*) to 26 (*F. anserina* and *F. dinganensis*) times (Fig 3 B). The long repeat sequences over 60 bp in length was the least, ~~and only~~ appearing in only~~ed~~ once ~~in all 13 genomes~~. The maximum length among all~~in all~~ the long repeat sequences was 64 bp.

Fig 3. Comparison of long repeat sequences among~~in~~ 13 *F. sarmentosa* complex genomes ~~in the *F. sarmentosa* complex~~. A, The numbers of each of four long repeat types (P, palindromic; F, forward; R; reverse; C complement); B, The numbers of long repeat sequences ~~with~~ of different lengths.

~~The s~~Simple sequence repeats (SSRs) ~~with~~ consisting of the ~~motifs of~~ 1- to 6- nucleotides motifs were ~~identified~~ surveyed across~~in~~ the 13 *F. sarmentosa* complex genomes ~~taxa, while hexan-ucleotide SSR was absent in all the genomes~~ (Fig 4 A). ~~In the remaining types of SSR, m~~Mono-nucleotide ~~repeats~~ SSRs were the most abundant ~~repeats~~ (78.71%) ~~among~~ across all 13 genomes. Dinucleotide SSRs, the ~~dominant~~ most commonly used ~~application type~~motif in population genetics and phylogenetics~~ analysis~~, were also~~as~~ relatively abundant, appearing ~~ranging from~~ from 19 (*F. howii*, *F. polynervis*, *F. sarmentosa* var. *lacrymans*, and *F. sarmentosa* var. *sarmentosa*) to 22 (*F. pumila* and *F. sarmentosa* var. *thunbergii*) times. It is noteworthy that ~~the frequency of~~ tetra-nucleotide SSRs were~~as~~

Formatted: Font: Not Italic

more common than ~~higher than that of~~ trinucleotide SSRs (Fig 4 A), ~~which ranged~~ considering the former appeared from 9 (*F. guizhouensis*, *F. polynervis*, *F. sarmentosa* var. *lacrymans*, and *F. sarmentosa* var. *sarmentosa*) to 11 (*F. pumila*, *F. sarmentosa* var. *henryi*, *F. sarmentosa* var. *impressa*, and *F. sarmentosa* var. *nipponica*) times. Hexanucleotide SSRs were absent from all genomes. The total number of SSRs varied from 175 (*F. dinganensis* and *F. howii*) to 190 (*F. polynervis*). ~~For~~ Of the repeat motifs, A/T was the most common mono-nucleotide motif, ~~which~~ appear~~ed~~ing from 132 ~~times~~ (*F. guizhouensis*) to 149 ~~times~~ (*F. polynervis* and *F. sarmentosa* var. *sarmentosa*) times (Fig 4 B). AT/AT was the second most common motif, appearing between ~~at~~ 18 (*F. howii*, *F. polynervis*, *F. sarmentosa* var. *lacrymans*, and *F. sarmentosa* var. *sarmentosa*) and~~to~~ 21 (*F. pumila* and *F. sarmentosa* var. *thunbergii*) times. T~~Most of t~~he marjority of the remainding motifs~~er~~ appeared only once to four times, with the exception of ~~except~~ AATT/AATT ~~repeat motif~~ appearing up to seven times (Fig 4 B).

Fig 4. Comparison of simple sequence repeats (SSRs) ~~of~~ among 13 *F. sarmentosa* complex genomes~~s in the *F. sarmentosa* complex~~. A, The numbers of SSRs ~~with~~ containing one- to five-nucleotide motifs ~~repeat units~~; B, The numbers of different SSR motifs.

Codon Usage

Across~~among the~~ all 13 *F. sarmentosa* complex ~~chloroplast~~ genomes, ~~of the *F. sarmentosa* complex.~~ ~~All~~ the 79 unique protein-coding~~on~~ CDS ~~sequences~~ regions were encoded by between 22,867 (*F. polynervis* and *F. sarmentosa* var. *lacrymans*) and~~to~~ 22,918 (*F. sarmentosa* var. *impressa*) codons. ~~among the 13 chloroplast genomes of the *F. sarmentosa* complex.~~ The codon usage among~~of~~ all 81 protein-coding genes is ~~was~~ summarizsed in Table 2. Among these codons, CAU encoding histidine

(H) was the most frequnent, withappearing 12,800 times altogether acrossin all the 13 taxa., while Except the stop codon, UGU encoding L-Cysteine (C) was the next rarest, only appearinged 718 times only and was the rarest codon except the stop codon. Based onAccording to the RSCU relative synonymous codon usage (RSCU)analysis, GCU and CUU had the highest average values with of 1.858 and 1.818, respectively (Table 2), whereas UAC and CGC had the lowest average valuesleast ones were UAC and CGC, whose values were of 0.364 and 0.400, respectively. Among all three stop codons Besides, UAA was the most frequent stop codon in all three stop codons with a ratio of common (53.64%). Thirty out of the 64 codons with RSCU over > 1 entirely ended with base either A or U, while 32 out of 64 codons with RSCU less than< 1 ended with either G or C, with the exception offor the codon AUA codon.

Table 2. The relative synonymous codon usage (RSCU) of all 64 codons. The taxa are representeding by the numbers indicated shown in Table 1.

Codon	AA	RSCU (relative synonymous codon usage)												
		1	2	3	4	5	6	7	8	9	10	11	12	13
UGA	*	0.662	0.662	0.662	0.662	0.662	0.662	0.662	0.662	0.662	0.662	0.662	0.662	0.662
UAG	*	0.701	0.701	0.74	0.701	0.74	0.701	0.74	0.74	0.74	0.74	0.74	0.74	0.74
UAA	*	1.636	1.636	1.597	1.636	1.597	1.636	1.597	1.597	1.597	1.597	1.597	1.597	1.597
GCU	A	1.853	1.853	1.853	1.853	1.862	1.853	1.854	1.861	1.863	1.869	1.861	1.862	1.857
GCG	A	0.413	0.413	0.413	0.413	0.415	0.413	0.412	0.404	0.404	0.411	0.404	0.415	0.413
GCC	A	0.594	0.594	0.6	0.597	0.589	0.594	0.592	0.591	0.591	0.589	0.591	0.589	0.59
GCA	A	1.14	1.14	1.135	1.136	1.134	1.14	1.142	1.144	1.143	1.132	1.144	1.134	1.141
UGU	C	1.57	1.575	1.569	1.569	1.561	1.561	1.569	1.569	1.569	1.561	1.569	1.561	1.569
UGC	C	0.43	0.425	0.431	0.431	0.439	0.439	0.431	0.431	0.431	0.439	0.431	0.439	0.431
GAU	D	1.596	1.596	1.596	1.596	1.596	1.598	1.594	1.595	1.595	1.597	1.595	1.598	1.593
GAC	D	0.404	0.404	0.404	0.404	0.404	0.402	0.406	0.405	0.405	0.403	0.405	0.402	0.407
GAG	E	0.462	0.467	0.464	0.464	0.459	0.463	0.464	0.465	0.466	0.461	0.465	0.459	0.463
GAA	E	1.538	1.533	1.536	1.536	1.541	1.537	1.536	1.535	1.534	1.539	1.535	1.541	1.537
UUU	F	1.368	1.367	1.374	1.367	1.373	1.369	1.372	1.37	1.37	1.372	1.371	1.373	1.37
UUC	F	0.632	0.633	0.626	0.633	0.627	0.631	0.628	0.63	0.63	0.628	0.629	0.627	0.63
GGU	G	1.34	1.34	1.338	1.338	1.341	1.334	1.342	1.337	1.338	1.342	1.337	1.341	1.342
GGG	G	0.609	0.606	0.61	0.612	0.612	0.609	0.609	0.612	0.61	0.613	0.612	0.612	0.609
GGC	G	0.404	0.402	0.404	0.407	0.397	0.407	0.402	0.405	0.404	0.396	0.405	0.397	0.402
GGA	G	1.647	1.652	1.648	1.643	1.651	1.649	1.647	1.647	1.648	1.649	1.647	1.651	1.647
CAC	H	0.463	0.468	0.46	0.465	0.457	0.463	0.465	0.464	0.464	0.457	0.464	0.457	0.464
CAU	H	1.537	1.532	1.54	1.535	1.543	1.537	1.535	1.536	1.536	1.543	1.536	1.543	1.536
AUU	I	1.471	1.471	1.471	1.477	1.47	1.473	1.471	1.468	1.467	1.47	1.468	1.47	1.47
AUA	I	0.965	0.967	0.959	0.965	0.965	0.966	0.962	0.964	0.963	0.963	0.964	0.965	0.964
AUC	I	0.564	0.562	0.57	0.558	0.565	0.561	0.567	0.568	0.57	0.567	0.568	0.565	0.566
AAA	K	1.531	1.534	1.534	1.533	1.528	1.53	1.534	1.536	1.537	1.527	1.536	1.528	1.539
AAG	K	0.469	0.466	0.466	0.467	0.472	0.47	0.466	0.464	0.463	0.473	0.464	0.472	0.461
CUA	L	1.162	1.16	1.169	1.166	1.145	1.169	1.158	1.155	1.154	1.146	1.155	1.145	1.16
CUC	L	0.521	0.522	0.528	0.518	0.52	0.521	0.518	0.516	0.516	0.52	0.516	0.52	0.515
CUG	L	0.504	0.501	0.5	0.501	0.513	0.504	0.504	0.506	0.505	0.513	0.506	0.513	0.505
CUU	L	1.813	1.816	1.802	1.814	1.823	1.806	1.82	1.823	1.825	1.821	1.823	1.823	1.819
UUA	L	1.249	1.251	1.25	1.249	1.254	1.249	1.25	1.254	1.254	1.251	1.254	1.252	1.25
UUG	L	0.751	0.749	0.75	0.751	0.746	0.751	0.75	0.746	0.746	0.749	0.746	0.748	0.75
AUG	M	1	1	1	1	1	1	1	1	1	1	1	1	1
AAC	N	0.446	0.444	0.442	0.446	0.452	0.446	0.444	0.443	0.443	0.454	0.443	0.453	0.445
AAU	N	1.554	1.556	1.558	1.554	1.548	1.554	1.556	1.557	1.557	1.546	1.557	1.547	1.555
CCA	P	1.166	1.16	1.165	1.163	1.154	1.166	1.162	1.162	1.165	1.158	1.162	1.154	1.16
CCC	P	0.68	0.681	0.679	0.677	0.678	0.676	0.68	0.684	0.684	0.678	0.684	0.678	0.684
CCU	P	1.592	1.596	1.586	1.598	1.596	1.597	1.592	1.592	1.591	1.596	1.592	1.596	1.591
CCG	P	0.562	0.563	0.57	0.562	0.573	0.562	0.566	0.562	0.561	0.568	0.562	0.573	0.565
CAA	Q	1.585	1.588	1.58	1.586	1.587	1.585	1.582	1.585	1.583	1.584	1.585	1.587	1.582
CAG	Q	0.415	0.412	0.42	0.414	0.413	0.415	0.418	0.415	0.417	0.416	0.415	0.413	0.418
AGA	R	1.496	1.5	1.5	1.497	1.502	1.494	1.496	1.499	1.501	1.502	1.499	1.502	1.497
AGG	R	0.504	0.5	0.5	0.503	0.498	0.506	0.504	0.501	0.499	0.498	0.501	0.498	0.503
CGA	R	1.6	1.596	1.604	1.596	1.602	1.601	1.596	1.591	1.594	1.602	1.591	1.602	1.596
CGC	R	0.405	0.399	0.398	0.399	0.403	0.399	0.399	0.399	0.398	0.403	0.399	0.403	0.399
CGG	R	0.426	0.425	0.419	0.425	0.423	0.43	0.414	0.419	0.419	0.423	0.419	0.423	0.414
CGU	R	1.569	1.581	1.579	1.581	1.571	1.57	1.591	1.591	1.589	1.571	1.591	1.571	1.591
AGC	S	0.447	0.456	0.455	0.46	0.451	0.45	0.454	0.454	0.454	0.45	0.454	0.447	0.454
AGU	S	1.553	1.544	1.545	1.54	1.549	1.55	1.546	1.546	1.546	1.55	1.546	1.553	1.546
UCA	S	1.111	1.111	1.115	1.114	1.116	1.107	1.111	1.109	1.108	1.116	1.11	1.116	1.109
UCC	S	0.817	0.817	0.809	0.817	0.803	0.814	0.806	0.812	0.816	0.8	0.813	0.803	0.808
UCG	S	0.449	0.449	0.448	0.452	0.448	0.452	0.45	0.448	0.447	0.448	0.448	0.448	0.446
UCU	S	1.624	1.622	1.628	1.617	1.632	1.627	1.632	1.631	1.629	1.635	1.629	1.632	1.636
ACC	T	0.701	0.699	0.701	0.701	0.701	0.701	0.702	0.695	0.697	0.702	0.695	0.701	0.699
ACA	T	1.187	1.189	1.182	1.185	1.177	1.185	1.176	1.177	1.179	1.179	1.177	1.177	1.174
ACG	T	0.417	0.416	0.422	0.417	0.417	0.417	0.422	0.426	0.425	0.418	0.426	0.417	0.426
ACU	T	1.695	1.696	1.695	1.697	1.706	1.697	1.7	1.701	1.698	1.702	1.701	1.706	1.701
GUU	V	1.463	1.462	1.461	1.468	1.466	1.466	1.461	1.458	1.461	1.46	1.458	1.466	1.461
GUG	V	0.513	0.511	0.522	0.513	0.513	0.51	0.513	0.515	0.521	0.516	0.515	0.513	0.513
GUC	V	0.405	0.406	0.405	0.403	0.409	0.405	0.406	0.411	0.407	0.415	0.411	0.409	0.406
GUA	V	1.618	1.621	1.613	1.616	1.612	1.618	1.62	1.616	1.612	1.609	1.616	1.612	1.62
UGG	W	1	1	1	1	1	1	1	1	1	1	1	1	1
UAC	Y	0.365	0.364	0.364	0.365	0.364	0.361	0.366	0.366	0.365	0.364	0.366	0.364	0.363
UAU	Y	1.635	1.636	1.636	1.635	1.636	1.639	1.634	1.634	1.635	1.636	1.634	1.636	1.637

Genomic~~e~~ divergence and hotspot regions

The divergence of ~~The divergence of the w~~whole sequences ~~of~~ among the 13 *F. sarmentosa* complex ~~13~~ genomes ~~in the *F. sarmentosa* complex~~ was analyzed using ~~illustrated~~ the ~~by~~ mVISTA online platform with *F. anserina* as a reference. The results showed that the full-length chloroplast genomes ~~of 13 taxa~~ were ~~prominently~~ largely conserved across all 13 taxa ~~the whole length under the taxonomical ranks of the species complex~~. The majority of variable sites were located in intergenic spacer regions~~s~~ (marked red in Fig 5). Interestingly, ~~The alignment also cursorily demonstrated that~~ IRs ~~regions~~ were found to be more conserved than ~~large and small single copy regions~~ either LSCs or SSCs.

Formatted: Indent: First line: 3 ch

Fig 5. Comparison of ~~c~~Complete chloroplast genomes ~~comparison~~ among 13 ~~of thirteen *Ficus*~~ taxa in the *F. sarmentosa* complex with *F. anserina* as a reference. Thick, ~~G~~gray ~~thick~~ arrows above the alignment indicate~~showed~~ the orientation and position of each gene~~s~~. A cut-off of 70% identity was chosen for the plots. The Y-axis represent~~sed~~ the identity percentage, ~~–~~ranging from 50 to 100%.

Formatted: Font: Bold

Nucleotide diversity (π) was calculated ~~F~~for ~~every~~ each gene and intergenic region~~, we also calculated nucleotide diversity (π)~~ to evaluate genetic differentiation and detect hyper-variable segments. ~~Within~~ Among 60 genes ~~with a~~>~~–~~ 200 bp in length ~~over 200 bp~~, *rps15*, *ycf1*, *rpoA*, *ndhF*, and *rpl22* ~~had~~ exhibited the highest nucleotide diversity ~~:~~~~at~~ 0.00535, 0.00462, 0.00456, 0.00427 and 0.00377, respectively (Fig 6). The alignment lengths of these five genes radically ~~were with great changes~~ranged from 282 to 5,748 bp ~~(Fig 6)~~. ~~Besides, there were also s~~Seven genes (*atpI*, *psbE*, *rpl33*, *rps18*, *psbH*, *rrn23*, and *psaC*) ~~that~~ were found to be ~~–~~identical, with ~~i.e.,~~ ~~their~~a nucleotide

diversity ~~was~~ of zero. The average diversity of the 60 genes was 0.001676~~.~~, while~~However,~~ the average diversity of intergenic spacer regions~~s~~ was 0.00435, ~~about~~ approximately 2.6 times ~~as much as~~ that of the genes (Fig 6). Among the intergenic spacer regions, ~~T~~the highest ~~value of~~ nucleotide diversity was exhibited by~~in intergenic spacers reached 0.01458 for~~ *trnH-GUG-psbA* (0.01458), ~~successively~~ followed by *rpl32-trnL-UAG* ~~0.01225~~ (0.01225~~*rpl32-trnL-UAG*~~), *psbZ-trnG-GCC*~~0.01148~~ (0.01148~~*psbZ-trnG-GCC*~~), *trnK-UUU-rps16*~~0.01144~~ (0.01144~~*trnK-UUU-rps16*~~), and *ndhF-rpl32* ~~0.01112~~ (0.01112~~*ndhF-rpl32*~~)~~,~~. The ~~with the~~ alignment lengths of these regions ranged~~ing~~ from 366 to 1,829 bp (Fig 6).

Fig 6. ~~**Bar chart of n**~~**Nucleotide diversity of** ~~**all the**~~ **genes and intergenic spacer**~~**s**~~ **regions** ~~**in**~~**among** ~~**thirteen**~~**13 taxa in the *F. sarmentosa* complex.** The alignment lengths are indicated ~~were shown~~ on the bars. The horizontal lines indicate~~d~~ the average~~the mean values of~~ nucleotide diversity of genes and intergenic spacers regions, respectively. The ~~first~~ top five genes or intergenic spacers with the highest nucleotide diversity ~~were~~ are highlighted in blue~~ color~~.

Selective pressure analysis

The ratio of nonsynonymous (Ka) to synonymous (Ks) substitution was calculated to ~~assess~~ quantify the evolutionary selective ~~stress~~ pressure on~~in~~ the *F. sarmentosa* complex, with *F. simplicissima* used as a~~the~~ reference genome ~~from *F. simplicissima*~~. Overall, ~~The result denoted that the~~ Ka/Ks ratios ~~values~~ of most genes were ~~less than~~< 1 (Fig 7). ~~Especially~~Additionally, twenty genes were found to contain~~there are twenty genes without any~~ no substitutions~~es~~, i.e., both Ka and Ks are zero (~~here~~ shown as "1" in Fig 7). However, five genes (*accD*, *matK*, *ndhF*, *rpoA*, and *ycf1*) had ~~the~~

partial ~~values of~~ Ka/Ks ratios ~~in five genes (*accD*, *matK*, *ndhF*, *rpoA*, and *ycf1*) were~~ over 1, ~~indicating~~ which are ~~the p~~potential signals of positive selection. ~~The frequencies of~~An infinite Ka/Ks ratios ~~value~~ (Ka > 0 and Ks = 0) ~~were compared and found that infinite value~~ existed in 27 genes (Fig 7). Notably, ~~there were~~ ten genes (*accD*, *clpP*, *ndhK*, *rbcL*, *rpl20*, *rpl22*, *rpl23*, *rpoC1*, *rps15*, and *rps4*) possessed~~ing~~ infinite Ka/Ks ratios ~~values~~ in ~~over~~ more than half of the taxa.

Fig 7. **Boxplot of Ka/Ks ~~value~~ ratios for 80 unique CDS regions.** The value 1.0 represents~~ed~~ the situation~~s~~ ~~when~~ where both Ka and Ks ~~are~~ equal zero. The~~L~~ line chart superimposed upon the boxplot demonstrates~~ed~~ the frequency of infinite Ka/Ks ratios ~~value~~ (~~viz.~~ Ka > 0 and Ks = 0), with detailed numbers labeled simultaneously.

Phylogenetic analysis

The chloroplast phylogenomic ~~maximum likelihood~~ ML tree ~~showed~~ illustrated a well-supported phylogenetic relationship in the genus *Ficus* (Fig 8). Subgenus *Pharmacosycea* sect. *Pharmacosycea* was strongly supported as a sister group to the ~~entire~~ rest of the genus *Ficus* (~~Fig 8,~~ SH-~~alrt~~aLRT = 100 and MLBS = 100)~~,~~. Additionally, ~~and~~ a clade including subg. *Urostigma* sect. *Galoglychia* and *Americana*, subg. *Sycomorus* sect. *Sycomorus*, and ~~some~~ a few species of subg. *Pharmacosycea* sect. *Oreosycea* (subser. *Ablbipilae* in Corner's system) (~~Fig 8,~~ clade A) ~~was~~ was found to be~~the~~ sister to the remainder of *Ficus* except ~~for~~ sect. *Pharmacosycea*. ~~Except for~~Aside from these two clades, the remaining ~~*fig trees*~~*Ficus* taxa formed three clades (clades B, C, and D in Fig 8), with unstable support (SH-~~alrt~~aLRT = 64.9 and MLBS = 73). Clade B (SH-~~alrt~~aLRT = 99.9 and MLBS = 100) was comprised of five different subgenera, ~~and~~ while clade C (SH-~~alrt~~aLRT = 100 and MLBS = 100)

Formatted: Font: Not Italic

Formatted: Font: Italic

included all species of subg. *Sycidium* ~~and~~ as well as ~~the~~ members of each of the~~all the~~ other five subgenera. ~~Whereas c~~Clade D (SH-~~alrt~~aLRT = 100 and MLBS = 100) ~~only consisted of~~contained only two species in subg. *Pharmacosycea* sect. *Oreosycea*. On the whole, all six subgenera were found not to be monophyletic~~ groups~~.

Fig 8. **The maximum likelihood (ML) phylogenetic tree of 123 chloroplast genomes in *Ficus* with six ~~genomes in~~ Olmoideae genomes as the outgroup.** Only the branches with ~~the~~ either SH-~~alrt~~aLRT or ultrafast bootstrap ~~less than~~< 95% were ~~noted~~ annotated by corresponding values. The starred tip names indicate~~d~~ ~~the~~ genomes obtained from ~~the research of~~ Bruun-Lund et al.; the red ~~ones~~ names indicate ~~meant~~ genomes ~~newly~~ obtained~~ genomes~~ in thise study; the blue ~~ones~~ names ~~showed~~indicate ~~the~~ members of the *F. auriculata* complex; and the bold names~~ones~~ indicate~~were~~ members of the *F. sarmentosa complex*. The ~~division of~~ subgenus~~enus~~ and section division of~~for~~ *Ficus* ~~was~~ are annotated ~~at~~ to the right of tip names. The topology of the ML tree ~~was~~ is shown in the upper left corner (excluding the outgroup).

Formatted: Font: Not Italic

~~With~~ With the ~~addition~~ inclusion of ~~other~~ eleven additional ~~available~~ samples, a phylogenomic analysis was carried out on ~~total of~~ 24 samples representing the 13 taxa in the *F. sarmentosa* complex ~~were used in the phylogenomics~~(Fig 8). The results showed that~~ the taxa of~~ the *F. sarmentosa* complex failed to form a monophyletic group. Except ~~that~~ three samples which are ~~were~~ unexpectedly embedded in a distinct clade ~~including~~ with other members of~~in~~ subg. *Synoecia* (~~Fig 8,~~ clade E), two ~~obvious~~ distinct lineages were recognized, with ~~and~~ the majority~~most~~ of nodes ~~within these two lineages were~~ within these two lineages being well-supported (~~Fig 8,~~ clades F and G). The six~~ varieties~~

~~of~~ *F. sarmentosa* varieties were found to be scattered across~~failed to cluster to be a monophyletic group and scattered throughout all three aforementioned clades (Fig 8,~~ clades E, F, and G~~)~~, ~~.~~ and ~~O~~only two individuals of ~~the variety~~ *F. sarmentosa* var. *lacrymans* clustered together. The six *F. pumila* samples ~~of *F. pumila* also~~ were also found not to be~~unable to comprise~~ a monophyletic group embedded by *F. sarmentosa* var. *thunbergii*.

Discussion

The differentiation and diversity of the chloroplast genome ~~in~~ in the *F. sarmentosa* complex~~closely related species~~

Formatted: Font: Italic

~~Till now~~To date, vast ~~studies of~~ comparative chloroplast genomic~~s~~ studies have been ~~reported~~ conducted~~for taxa at~~ in a wide ~~variety~~ range of taxonomic levels, such as order (such as Dipsacales [56] and~~,~~ Saxifragales [57]), family (such as Orchidaceae [58] and~~,~~ Zingiberaceae [59]), and genus (such as *Camellia* [60], *Lindera* [61], *Gossypium* [62], and *Ficus* [17, 51, 63]). However, less research ~~touched~~ has focused on the comparative genomics of ~~the~~ taxa undergoing ~~the~~ recent speciation, such as the species complex [29]. Al~~T~~though the structural~~e~~ conservation of the chloroplast genome at ~~the~~ low taxonomic~~al~~ levels is well-~~acknowledged~~ characterized [64-67], the detailed patterns of genomic differentiation and diversity ~~in~~ among closely related species remain~~s~~ largely unknown. Therefore, ~~the deep and~~ comprehensive comparisons ~~of~~ between ~~these~~ closely related species are necessary to improve our ~~insights~~ understanding of~~into~~ the mechanisms, rates, or directionality of genome evolution ~~in~~ during the early stages after speciation [29].

In this study, we investigated the evolutionary ~~pattern~~ dynamics of thirteen high-quality chloroplast genomes from the *F. sarmentosa* complex. Overall, ~~The results demonstrated that~~ the length of both ~~the~~ whole genome~~s~~ and quadripartite regions were ~~extremely~~ quite similar ~~with~~among taxa, with ~~the~~

~~largest variations at~~only 0.2288%, 0.3784%, 0.2188%, and 0.1236% variation among~~for~~ ~~the~~ whole genomes, LSCs, SSCs, and IRs, respectively (Table 1, Fig 1). Furthermore, the number, content, and orientation of annotated genes among all 13 genomes were identical. The IR boundaries (JLB, JSB, JSA, and JLA) were also relatively coincident ~~in~~ among the 13 genomes, being ~~and~~ located at the same loci with only slight variation ~~at~~ in the distance to the starting or ending bases ~~placement of the located loci~~. For ~~the~~ long repeat sequences, ~~it figured out that~~ all four repeat units were shared among all 13 plastomes, and the number of repeat units and their proportions ~~were~~ exhibited only~~similar with~~ slight differentiation. For example, ~~, such as~~ the proportion of palindromic sequences ranged from 45.16~~%~~ to 61.54% (Fig 3). Similarly, ~~The same rule also took effect in the~~in SSR regions, ~~with~~ the proportion of the mono~~-~~nucleotide repeat units ranged~~ing~~ from 72.63~~%~~ to 85.96% (Fig 4).

~~Obviously, t~~The high conservation ~~was detected~~exhibited across~~in~~ the *F. sarmentosa* complex~~,~~ ~~which~~ is consistent with ~~several~~ other studies on ~~other~~ closely related taxa. For example, ~~the~~ a study ~~based on~~of 22 closely related *Oryza* species ~~demonstrated~~ indicated that ~~the~~ conservation was ~~general~~ common ~~in~~ at ~~the~~ lower taxonomic~~al~~ ~~ranks~~ levels [29]. A study of four peanut varieties serves as a more~~nother~~ extreme ~~exampl~~example, reporting~~e is from four peanut botanical varieties where ther~~ perfectly ~~e is no difference~~identical ~~in the junction positions of~~ IR boundary junction positions~~ies~~ [68]. Al~~T~~though ~~studies of~~ comparative chloroplast genomic~~s~~ studies have rarely focused on closely related species~~ till now~~, the high conservation of ~~the~~ chloroplast genome among closely related species is recognized ~~and reasonable~~ based on our work and other ~~existing~~ related research.

Chloroplast genomic ~~The~~ evolutionary hotspots ~~of chloroplast genomes~~

Al~~T~~though chloroplast genomes exhibit high conservation among~~between~~ closely related species

are of the conserved structure, discrepancy and heterogeneity have also been widely observed across the whole genomes. GenerallyOverall, the variable sites of the two single -copy regions (LSC and SSC) are more abundant than IR repeat regions in both the genus *Ficus* (Fig 5) and most of the other plant groups [56, 69, 70]. CThe chloroplast regions with different mutation rates are appropriate for a range of to a diversity of evolutionary research. In general, conservative coding genes are suitable for deeply phylogenetic inferences at the family-level [28, 71, 72] or even higher [73-75], whereas highly variable regions are appropriate tofor studies of biogeography, species delimitation, population genetics, and phylogenetic reconstruction at lower infra-generic rank levels [76, 77].

NowadaysTo date, over more than 20 regions have been recommended as alternative loci in for phylogenetics, species delimitation, and barcoding, such as including *matK*, *rbcL*, *trnH-psbA*, *ycf1*, *ycf1-ndhF*, and so onamong others [77-79]. NeverthelessHowever, these loci have relativelyappeared poor preferences at the aspect of informative sites in the *F. sarmentosa* complex (nucleotide diversity Pi, Fig 6), indicating suggesting that the evolutionary heterogeneity of chloroplast loci may beis relatively common among different plant groups. In this study, we mined five hyper-variable intergenic regions at the level of the species complex, i.e., *trnH-GUG-psbA*, *rpl32-trnL-UAG*, *psbZ-trnG-GCC*, *trnK-UUU-rps16* and *ndhF-rpl32*. However, another other studies of comparative chloroplast genomicsison research on plastomes in *Ficus* identified entirely different hyper-variable intergenic regions. For example, Xia et al. [63] identified (such as *trnS-GCU-trnG-UCC*, *trnT-GGU-psbD*, *trnV-UAC-trnM-CAU*, *clpP-psbB*, *ndhF-trnL-UAG*, *trnL-UAG-ccsA*, *ndhD-psaC*, and *ycf1*, and Zhang et al., in the work of Xia et al. [63] as well as [17] identified *trnL-UAG-rpl32*, *trnE-UUC-psbD*, *trnK-UUU-rps16*, *rpoB-trnC-GCA*, and *petN-psbM* in Zhang et al. [17]). These disparate resultsfacts suggest that the evolutionary dynamicsrates of chloroplast genomes varyied acrossat both the groups and taxonomical ranks. Therefore, we suggest

Formatted: Font: Not Italic

Formatted: Font: Not Italic

Formatted: Font: Not Italic

Formatted: Font: Not Italic

that ~~it is ne~~chloroplast loci should be~~cessary and meaningful to choose alternative chloroplast loci~~ chosen cautiously according to the research objective ~~aim~~, plant ~~focused~~ group, and taxonomic~~al~~ level.

The plastomice phylogeny of the genus *Ficus* and the phylogenetic performance~~reference~~ in closely related species

Based on a compilation of nearly all ~~the~~ available *Ficus* chloroplast genomes ~~in *Ficus*~~, we obtained a robust ~~maximum likelihood~~ML phylogenetic tree with ~~most~~ the majority of nodes ~~under~~ exhibiting high bootstrap and SH-~~alrt~~aLRT values, ~~especially for~~particularly the deep nodes (Fig 8). Overall, ~~O~~our ML phylogenetic tree ~~based on the extended samples~~ is ~~generally~~ largely consistent with previous research [17, 26], ~~such as~~including the systematic position of subg. *Pharmacosycea* sect. *Pharmacosycea* and ~~some~~ the mysterious displacment of certain ~~mysteriously displaced st~~ individuals (*F. fulva*, *F. magnoliifolia*, *F. albipila*, *F. albert-smithii*, and *F. pumila*). However, analysis of ~~based on the~~our extended dataset highlighted an increase in~~, more~~ non-monophyletic groups ~~were discovered~~, such as subg. *Urostigma* sect. *Urostigma* and *Conosycea* (Fig 8). Notably, there ~~are~~were numerous incongruences between the chloroplast cladogram and previously published nuclear trees. For example, the chloroplast-based tree failed to support either *Ficus* subg. *Sycomorus* ~~and~~ or subg. *Sycidium* as monophyletic groups, whereas ~~they~~ these groups are well~~-~~confirmed in nuclear phylogeniesy [3, 12-14, 80]. Subg. *Synoecia* was~~is~~ also divided into three different clades (Fig 8). Further research into ~~Additionally, these~~the ~~mysteriously~~ displaced species, ~~for instance~~such as~~,~~ *F. fulva*, *F. ablert-smithii*, *F. magnoliilolia*, and *F. albipila* ~~(Fig 8)~~, ~~might~~ may reveal ~~the clew of~~ host shifts or nonspecific pollination ~~or~~ between fig trees and fig wasps [26]. ~~Whereas, c~~Considering that a stable and comprehensively-sampled~~ing~~ nuclear phylogenomic framework for the genus *Ficus* is still lacking ~~to date~~, ~~the~~ precise identification of these hybridization events ~~needs~~ will require more robust nuclear genom~~e~~ic data ~~and~~

Formatted: Font: Italic

~~even~~as well as ~~the~~ data from the associated fig wasps.

Compared to the nuclear phylogeny [4], ~~For the *F. sarmentosa* complex,~~ we ~~a~~discovered a disparate evolutionary relationship among taxa in the *F. sarmentosa* complex.~~,~~ including three distinct clades (Fig 8, clades E, F, and G)~~ was discovered compared to the nuclear phylogeny [4]~~. ~~In clade E, t~~Three ~~strangely displaced~~ samples from the complex dispersed into the clade E mixing with~~including~~ *F. sagittata* and the members of subg. *Synoecia* sect. *Apiosycea*. ~~Only if~~Unless more data support ~~the evidence of~~ hybridization, ~~the~~ misidentification may~~ight~~ be an ~~briefer~~ alternative explanation. ~~In the clades F and G,~~ ~~n~~Neither~~o~~ geographic nor morphological traits could be detected to support the split of clades F and G~~such phylogenetic analysis~~. ~~In addition, the h~~Hybridization between *F. pumila* and *F. sarmentosa* var. *thunbergii* might exist, considering that the latter embedded into the former within clade G. Although ~~the~~ our current samples are ~~deficient~~ insufficient to fully resolve the taxonomy ~~ical mystery in~~of the *F. sarmentosa* complex, the phylogenetic resolution provided by~~of~~ chloroplast genomes in the complex ~~should be~~ appears to be promising, ~~in the view the fact that~~ as almost all the nodes are strongly supported. The chloroplast genome has also shown ~~Such a promising~~high discriminability ~~of the chloroplast genome is also confirmed in~~across the *F. auriculata* complex (Fig 8, blue labels) [81-83]. The relationships ~~of~~ between four taxa (*F. auriculata*, *F. oligodon*, *F. hainanensis*, and *F. beipeiensis*) in the complex ~~was~~ were well-resolved, while *F. beipeiensis* ~~has~~ shared a distinct phylogenetic ~~place~~ relationship ~~together~~ with the climbing fig tree *F. tikoua*. Moreover, a new linage (*F. northern*) was identified based on ~~the~~ chloroplast genomics~~es~~, ~~indicating~~ suggesting a promising segue into further ~~phylogenetic resolution in~~ exploration~~ring~~ of the cryptic species (Fig 8, blue labels) [17]. Chloroplast genomes have been used to reconstruct high-resolution phylogenetic trees i~~I~~n ~~some~~ other closely related species groups, such as peanut [84], rice [29], and Orchardgrass [85]~~, chloroplast genomes often can acquire high resolution~~

~~phylogenetic trees~~. ~~Therefore~~Taken together, the chloroplast genome ~~shows~~ appears to be ~~an expectant~~a promising ~~resolution~~ tool for~~in~~ exploring the evolutionary relationships between closely related species and even ~~the~~ species complexes, although cyto-nuclear discordances often exist~~s~~.

Conclusions

In this study, ~~The~~ eleven *F. sarmentosa* complex chloroplast genomes ~~in the *F. sarmentosa* complex in Moraceae~~ were newly sequenced and characterized. ~~Furthermore, we compared the s~~Sequence lengths, IR boundaries~~y~~, repeat sequences, and codon usage were compared among ~~the~~ these eleven, ~~11~~ and ~~another~~ two previ~~downloaded~~ously-reported, chloroplast genomes ~~in the complex.~~, indicating that these parameters were ~~Ex~~ highly~~treme~~ conserved ~~ation was detected in all the respects aforementioned~~across taxa. However, ~~obvious~~ heterogeneity ~~also~~ was found ~~exists~~ in both nucleotide diversity and selective pressure among segments. ~~Therefore, w~~We ~~recommended~~ characterized ten~~–~~ evolutionary hotspot regions (*rps15*, *ycf1*, *rpoA*, *ndhF*, *rpl22*, *trnH*-GUG-*psbA*, *rpl32-trnL-UAG*, *psbZ-trnG-GCC*, *trnK-UUU-rps*16 and *ndhF-rpl32*). Phylogenomic analysis~~construction~~ indicated that chloroplast genomes show ~~a~~ promising~~e~~ ~~resolution~~ for inferring the phylogenetic relationships between~~of the~~ closely related groups, despite~~e the evident~~ cyto-nuclear discordance.

Acknowledgments

We are grateful to Dr. Hong-Qing Li at East China Normal University, Dr. Zhi-Hui Su and Ms. Sasaki Ayako at Osaka University, Dr. Yong Chen and Dr. Kai-Liang Liu at Ningde Normal University, and Mr. Zhen Liu ~~in~~ at Forestry and Grassland Administration of Motuo County for their support in wild collecting. The authors would like to thank TopEdit (www.topeditsci.com) for its linguistic assistance

during the preparation of this manuscript.

Funding

This research was funded by National Natural Science Foundation of China from Dr. De-Shun Zhang (grant number 32071824). The funder De-Shun Zhang implemented the conceptualization, visualization, and draft review of thise study.

Competing Interests

NO authors have competing interests.

Data Availability Statement

The eleven newly obtained complete chloroplast genome sequences that support the findings in the study are deposited in the NCBI with accession numbers as followed: ON257105-ON257115. The corresponding high throughput Illumina sequencing reads are deposited to Genome Sequence Archive (GSA) in BIG Data Center (https://bigd.big.ac.cn/gsa/) under BioProject accession: PRJCA012975.

Author Contributions

Conceptualization: Chi-Yuan Yao, De-Shun Zhang.

Data curation: Lu Zou.

Formal analysis: Zhen Zhang.

Funding acquisition: De-Shun Zhang.

Investigation: Zhen Zhang.

Methodology: Zhen Zhang.

Project administration: Chi-Yuan Yao.

Resources: Chi-Yuan Yao.

Software: Zhen Zhang.

Writing—original draft: Zhen Zhang.

Writing—review & editing: Zhen Zhang, De-Shun Zhang, Lu Zou, and Chi-Yuan Yao.

Visualization: De-Shun Zhang.

Formatted: Indent: First line: 0 ch

References

1. Berg CC, Corner EJH. Moraceae (*Ficus*). In: Nooteboom HP, editor. Flora Malesiana. 17. Leiden: National Herbarium of the Netherlands; 2005. p. 1-730.
2. Clement WL, Weiblen GD. Morphological evolution in the mulberry family (Moraceae). Syst Bot. 2009;34(3):530-52. doi: 10.1600/036364409789271155.
3. Clement WL, Bruun-Lund S, Cohen A, Kjellberg F, Weiblen GD, Rønsted N. Evolution and classification of figs (*Ficus*, Moraceae) and their close relatives (Castilleae) united by involucral bracts. Bot J Linn Soc. 2020;193(3):316-39. doi: 10.1093/botlinnean/boaa022
4. Zhang Z, Wang XM, Liao S, Zhang JH, Li HQ. Phylogenetic reconstruction of *Ficus* subg. *Synoecia* and its allies (Moraceae), with implications on the origin of the climbing habit. Taxon. 2020;69(5):927-45. doi: 10.1002/tax.12282.
5. Machado CA, Robbins N, Gilbert MTP, Herre EA. Critical review of host specificity and its coevolutionary implications in the fig/fig-wasp mutualism. P Natl Acad Sci USA. 2005;102(suppl

1):6558-65. doi: 10.1073/pnas.0501840102.

6. Wachi N, Ku sumi J, Tzeng HY, Su ZH. Genome- wide sequence data suggest the possibility of pollinator sharing by host shift in dioecious figs (Moraceae, *Ficus*). Mol Ecol. 2016;25(22):5732-46. doi: 10.1111/mec.13876.

7. Wang G, Cannon CH, Chen J. Pollinator sharing and gene flow among closely related sympatric dioecious fig taxa. P Roy Soc B-Biol Sci. 2016;283(1828):20152963. doi: 10.1098/rspb.2015.2963.

8. Yu H, Liao YL, Cheng YF, Jia YJ, Compton SG. More examples of breakdown the 1:1 partner specificity between figs and fig wasps. Bot Stud. 2021;62(1):1-12. doi: 10.1186/s40529-021-00323-8.

9. Yu H, Zhao NX, Yao J, Chen YZ. The influence of species-specific coevolution of figs and fig wasps on *Ficus* (Moraceae) classification. J Trop Subtrop Bot. 2006;14(5):439-43. doi: 10.3969/j.issn.1005-3395.2006.5.014.

10. Rønsted N, Weiblen GD, Cook JM, Salamin N, Machado CA, Savolainen V. 60 million years of co-divergence in the fig–wasp symbiosis. P Roy Soc B-Biol Sci. 2005;272(1581):2593-9. doi: 10.1098/rspb.2005.3249.

11. Rønsted N, Weiblen GD, Clement W, Zerega N, Savolainen V. Reconstructing the phylogeny of figs (*Ficus*, Moraceae) to reveal the history of the fig pollination mutualism. Symbiosis. 2008;45:45-55.

12. Xu L, Harrison RD, Yang P, Yang DR. New insight into the phylogenetic and biogeographic history of genus *Ficus*: Vicariance played a relatively minor role compared with ecological opportunity and dispersal. J Syst Evol. 2011;49(6):546-57. doi: 10.1111/j.1759-

6831.2011.00155.x.

13. Cruaud A, Rønsted N, Chantarasuwan B, Chou LS, Clement WL, Couloux A, et al. An extreme case of plant–insect codiversification: figs and fig-pollinating wasps. Syst Biol. 2012;61(6):1029-47. doi: 10.1093/sysbio/sys068.

14. Zhang Q, Onstein RE, Little SA, Sauquet H. Estimating divergence times and ancestral breeding systems in *Ficus* and Moraceae. Ann Bot. 2019;123(1):191-204. doi: 10.1093/aob/mcy159.

15. Zhang XT, Wang G, Zhang SC, Chen S, Wang YB, Wen P, et al. Genomes of the banyan tree and pollinator wasp provide insights into fig-wasp coevolution. Cell. 2020;183(4):875-89. e17. doi: 10.1016/j.cell.2020.09.043.

16. Rasplus JY, Rodriguez LJ, Sauné L, Peng YQ, Bain A, Kjellberg F, et al. Exploring systematic biases, rooting methods and morphological evidence to unravel the evolutionary history of the genus *Ficus* (Moraceae). Cladistics. 2021;37(4):402-22. doi: 10.1111/cla.12443.

17. Zhang ZR, Yang X, Li WY, Peng YQ, Gao J. Comparative chloroplast genome analysis of *Ficus* (Moraceae): Insight into adaptive evolution and mutational hotspot regions. Front Plant Sci. 2022;13:1-17. doi: 10.3389/fpls.2022.965335.

18. Soltis DE, Albert VA, Savolainen V, Hilu K, Qiu YL, Chase MW, et al. Genome-scale data, angiosperm relationships, and 'ending incongruence': a cautionary tale in phylogenetics. Trends Plant Sci. 2004;9(10):477-83. doi: 10.1016/j.tplants.2004.08.008.

19. Jansen RK, Cai ZQ, Raubeson LA, Daniell H, Depamphilis CW, Leebens-Mack J, et al. Analysis of 81 genes from 64 plastid genomes resolves relationships in angiosperms and identifies genome-scale evolutionary patterns. P Natl Acad Sci USA. 2007;104(49):19369-74. doi: 10.1073/pnas.0709121104.

20. Jin JJ, Yu WB, Yang JB, Song Y, DePamphilis CW, Yi TS, et al. GetOrganelle: a fast and versatile toolkit for accurate de novo assembly of organelle genomes. Genome Biol. 2020;21(1):1-31. doi: 10.1186/s13059-020-02154-5.

21. Dierckxsens N, Mardulyn P, Smits G. NOVOPlasty: de novo assembly of organelle genomes from whole genome data. Nucleic Acids Res. 2017;45(4):e18-e. doi: 10.1093/nar/gkw955.

22. Yang JB, Tang M, Li HT, Zhang ZR, Li DZ. Complete chloroplast genome of the genus *Cymbidium*: lights into the species identification, phylogenetic implications and population genetic analyses. BMC Evol Biol. 2013;13(1):1-12. doi: 10.1186/1471-2148-13-84.

23. Li XW, Yang Y, Henry RJ, Rossetto M, Wang Y, Chen SL. Plant DNA barcoding: from gene to genome. Biol Rev. 2015;90(1):157-66. doi: 10.1111/brv.12104.

24. Tonti- Filippini J, Nevill PG, Dixon K, Small I. What can we do with 1000 plastid genomes? Plant J. 2017;90(4):808-18. doi: 10.1111/tpj.13491.

25. Zhang Z, Zhang DS. The complete chloroplast genome sequence of *Ficus sarmentosa* (Moraceae, Rosales), a widely distributed fig tree in East Asia. Mitochondrial DNA B. 2022;7(9):1597-8. doi: 10.1080/23802359.2022.2115860.

26. Bruun-Lund S, Clement WL, Kjellberg F, Rønsted N. First plastid phylogenomic study reveals potential cyto-nuclear discordance in the evolutionary history of *Ficus* L. (Moraceae). Mol Phylogenet Evol. 2017;109:93-104. doi: 10.1016/j.ympev.2016.12.031.

27. Roure B, Baurain D, Philippe H. Impact of missing data on phylogenies inferred from empirical phylogenomic data sets. Mol Biol Evol. 2013;30(1):197-214. doi: 10.1093/molbev/mss208.

28. Zhang SD, Jin JJ, Chen SY, Chase MW, Soltis DE, Li HT, et al. Diversification of Rosaceae since the Late Cretaceous based on plastid phylogenomics. New Phytol. 2017;214(3):1355-67. doi:

10.1111/nph.14461.
29. Gao LZ, Liu YL, Zhang D, Li W, Gao J, Liu Y, et al. Evolution of *Oryza* chloroplast genomes promoted adaptation to diverse ecological habitats. Commun Biol. 2019;2(1):1-13. doi: 10.1038/s42003-019-0531-2.
30. Liu ZF, Ma H, Ci XQ, Li L, Song Y, Liu B, et al. Can plastid genome sequencing be used for species identification in Lauraceae? Bot J Linn Soc. 2021;197(1):1-14. doi: 10.1093/botlinnean/boab018.
31. Berg CC. Flora Malesiana precursor for the treatment of Moraceae 4: *Ficus* subgenus *Synoecia*. Blumea. 2003;48(3):551-71. doi: 10.3767/000651903X489546.
32. Corner EJH. Taxonomic notes on *Ficus* L., Asia and Australasia. Gard Bull Singapore. 1960;18(1):1-69.
33. Corner EJH. Check-list of *Ficus* in Asia and Australasia with keys to identification. Gard Bull Singapore. 1965;21:1-186.
34. Chang SS. Three new species of the Moraceae from China. Acta Phytotax Sin. 1982;20(1):95-8.
35. Chang SS. New taxa of Moraceae from China. Guihaia. 1983;3(4):295-306.
36. Chang SS. New Taxa of Moraceae from China and Vietnam. Acta Phytotax Sin. 1984;22(1):64-76.
37. Chang SS, Wu CY, Cao ZY. Moraceae (*Ficus*). In: Chang SS, Wu CY, editors. Flora Reipublicae Popularis Sinicae. 23. Beijing: Science Press; 1998. p. 66-169.
38. Yamazaki T. Taxonomic review of the Moraceae from Japan, Korea, Taiwan and adjacent areas (2). J Phytogeo Taxon. 1983;31(1):1-15.
39. Tsai L, Hayakawa H, Fukuda T, Yokoyama J. A breakdown of obligate mutualism on a small island: An interspecific hybridization between closely related fig species (*Ficus pumila* and *Ficus*

thunbergii) in Western Japan. Amer J Pl Sci. 2015;6(01):126-31. doi: 10.4236/ajps.2015.61014.

40. Berg CC. Precursory taxonomic studies on *Ficus* (Moraceae) for the Flora of Thailand. Thai Forest Bull. 2007;35:4-28.

41. Doyle JJ, Doyle JL. A rapid DNA isolation procedure for small quantities of fresh leaf tissue. Phytochem Bull. 1987;19:11-5.

42. Qu XJ, Moore MJ, Li DZ, Yi TS. PGA: a software package for rapid, accurate, and flexible batch annotation of plastomes. Plant Methods. 2019;15(1):1-12. doi: 10.1186/s13007-019-0435-7.

43. Greiner S, Lehwark P, Bock R. Organellar Genome DRAW (OGDRAW) version 1.3.1: expanded toolkit for the graphical visualization of organellar genomes. Nucleic Acids Res. 2019;47(W1):W59-W64. doi: 10.1093/nar/gkz238.

44. Amiryousefi A, Hyvönen J, Poczai P. IRscope: an online program to visualize the junction sites of chloroplast genomes. Bioinformatics. 2018;34(17):3030-1. doi: 10.1093/bioinformatics/bty220.

45. Kurtz S, Choudhuri JV, Ohlebusch E, Schleiermacher C, Stoye J, Giegerich R. REPuter: the manifold applications of repeat analysis on a genomic scale. Nucleic Acids Res. 2001;29(22):4633-42. doi: 10.1093/nar/29.22.4633.

46. Beier S, Thiel T, Münch T, Scholz U, Mascher M. MISA-web: a web server for microsatellite prediction. Bioinformatics. 2017;33(16):2583-5. doi: 10.1093/bioinformatics/btx198.

47. Frazer KA, Pachter L, Poliakov A, Rubin EM, Dubchak I. VISTA: computational tools for comparative genomics. Nucleic Acids Res. 2004;32(suppl_2):W273-W9. doi: 10.1093/nar/gkh458.

48. Edgar RC. MUSCLE: multiple sequence alignment with high accuracy and high throughput. Nucleic Acids Res. 2004;32(5):1792-7. doi: 10.1093/nar/gkh340.

49. Xia X. DAMBE 6: new tools for microbial genomics, phylogenetics, and molecular evolution. J Hered. 2017;108(4):431-7. doi: 10.1093/jhered/esx033.

50. Chen CJ, Chen H, Zhang Y, Thomas HR, Frank MH, He YH, et al. TBtools: an integrative toolkit developed for interactive analyses of big biological data. Mol Plant. 2020;13(8):1194-202. doi: 10.1016/j.molp.2020.06.009.

51. Huang YY, Li J, Yang ZR, An WL, Xie CZ, Liu SS, et al. Comprehensive analysis of complete chloroplast genome and phylogenetic aspects of ten *Ficus* species. BMC Plant Biol. 2022;22(1):1-15. doi: 10.1186/s12870-022-03643-4.

52. Gardner EM, Garner M, Cowan R, Dodsworth S, Epitawalage N, Arifiani D, et al. Repeated parallel losses of inflexed stamens in Moraceae: phylogenomics and generic revision of the tribe Moreae and the reinstatement of the tribe Olmedieae (Moraceae). Taxon. 2021:946-88. doi: 10.1002/tax.12526.

53. Katoh K, Standley DM. MAFFT multiple sequence alignment software version 7: improvements in performance and usability. Mol Biol Evol. 2013;30(4):772-80. doi: 10.1093/molbev/mst010.

54. Capella-Gutiérrez S, Silla-Martínez JM, Gabaldón T. trimAl: a tool for automated alignment trimming in large-scale phylogenetic analyses. Bioinformatics. 2009;25(15):1972-3. doi: 10.1093/bioinformatics/btp348.

55. Kalyaanamoorthy S, Minh BQ, Wong TK, Von Haeseler A, Jermiin LS. ModelFinder: fast model selection for accurate phylogenetic estimates. Nat Methods. 2017;14(6):587-9. doi: 10.1038/nmeth.4285.

56. Fan WB, Wu Y, Yang J, Shahzad K, Li ZH. Comparative chloroplast genomics of Dipsacales species: insights into sequence variation, adaptive evolution, and phylogenetic relationships. Front

Plant Sci. 2018;9:1-13. doi: 10.3389/fpls.2018.00689.

57. Dong WP, Xu C, Cheng T, Zhou SL. Complete chloroplast genome of Sedum sarmentosum and chloroplast genome evolution in Saxifragales. PloS One. 2013;8(10):e77965. doi: 10.1371/journal.pone.0077965.

58. Luo J, Hou BW, Niu ZT, Liu W, Xue QY, Ding XY. Comparative chloroplast genomes of photosynthetic orchids: insights into evolution of the Orchidaceae and development of molecular markers for phylogenetic applications. PloS One. 2014;9(6):e99016. doi: 10.1371/journal.pone.0099016.

59. Gao BM, Yuan L, Tang TL, Hou J, Pan K, Wei N. The complete chloroplast genome sequence of *Alpinia oxyphylla* Miq. and comparison analysis within the Zingiberaceae family. PloS One. 2019;14(6):e0218817. doi: 10.1371/journal.pone.0218817.

60. Yang JB, Yang SX, Li HT, Yang J, Li DZ. Comparative chloroplast genomes of *Camellia* species. PloS One. 2013;8(8):e73053. doi: 10.1371/journal.pone.0073053.

61. Zhao ML, Song Y, Ni J, Yao X, Tan YH, Xu ZF. Comparative chloroplast genomics and phylogenetics of nine *Lindera* species (Lauraceae). Sci Rep. 2018;8(1):1-11. doi: 10.1038/s41598-018-27090-0.

62. Wu Y, Liu F, Yang DG, Li W, Zhou XJ, Pei XY, et al. Comparative chloroplast genomics of *Gossypium* species: insights into repeat sequence variations and phylogeny. Front Plant Sci. 2018;9:376. doi: 10.3389/fpls.2018.00376.

63. Xia X, Peng JY, Yang L, Zhao XL, Duan AA, Wang DW. Comparative Analysis of the Complete Chloroplast Genomes of Eight *Ficus* Species and Insights into the Phylogenetic Relationships of *Ficus*. Life. 2022;12(6):848. doi: 10.3390/life12060848.

64. Palmer JD. Comparative organization of chloroplast genomes. Annu Rev Genet. 1985;19(1):325-54. doi: 10.1146/annurev.ge.19.120185.001545.
65. Sugiura M. The chloroplast genome. Plant Mol Biol. 1992:149-68. doi: 10.1007/978-94-011-2656-4_10.
66. Jansen RK, Ruhlman TA. Plastid genomes of seed plants. In: Bock R, Knoop V, editors. Genomics of Chloroplasts and Mitochondria. Dordrecht: Springer; 2012. p. 103-26.
67. Mower JP, Vickrey TL. Structural diversity among plastid genomes of land plants. Adv Bot Res. 2018;85:263-92. doi: 10.1016/bs.abr.2017.11.013.
68. Wang J, Li CJ, Yan CX, Zhao XB, Shan SH. A comparative analysis of the complete chloroplast genome sequences of four peanut botanical varieties. PeerJ. 2018;6:e5349. doi: 10.7717/peerj.5349.
69. Li XQ, Zuo YJ, Zhu XX, Liao S, Ma JS. Complete chloroplast genomes and comparative analysis of sequences evolution among seven *Aristolochia* (Aristolochiaceae) medicinal species. Int J Mol Sci. 2019;20(5):1045. doi: 10.3390/ijms20051045. PubMed PMID: doi:10.3390/ijms20051045.
70. Guo XX, Qu XJ, Zhang XJ, Fan SJ. Comparative and phylogenetic analysis of complete plastomes among Aristidoideae species (Poaceae). Biology. 2022;11(1):63. doi: 10.3390/biology11010063. PubMed PMID: doi:10.3390/biology11010063.
71. Zhang R, Wang YH, Jin JJ, Stull GW, Bruneau A, Cardoso D, et al. Exploration of plastid phylogenomic conflict yields new insights into the deep relationships of Leguminosae. Syst Biol. 2020;69(4):613-22. doi: 10.1093/sysbio/syaa047.
72. Liu LM, Du XY, Guo C, Li DZ. Resolving robust phylogenetic relationships of core Brassicaceae using genome skimming data. J Syst Evol. 2021;59(3):442-53. doi: 10.1111/jse.12666.

73. Moore MJ, Soltis PS, Bell CD, Burleigh JG, Soltis DE. Phylogenetic analysis of 83 plastid genes further resolves the early diversification of eudicots. P Natl Acad Sci USA. 2010;107(10):4623-8. doi: 10.1073/pnas.0907801107.

74. Gitzendanner MA, Soltis PS, Wong GKS, Ruhfel BR, Soltis DE. Plastid phylogenomic analysis of green plants: a billion years of evolutionary history. Am J Bot. 2018;105(3):291-301. doi: 10.1002/ajb2.1048.

75. Li HT, Luo Y, Gan L, Ma PF, Gao LM, Yang JB, et al. Plastid phylogenomic insights into relationships of all flowering plant families. BMC Biology. 2021;19(1):1-13. doi: 10.1186/s12915-021-01166-2.

76. Dong WP, Liu J, Yu J, Wang L, Zhou SL. Highly variable chloroplast markers for evaluating plant phylogeny at low taxonomic levels and for DNA barcoding. PloS One. 2012;7(4):e35071. doi: 10.1371/journal.pone.0035071.

77. Shaw J, Shafer HL, Leonard OR, Kovach MJ, Schorr M, Morris AB. Chloroplast DNA sequence utility for the lowest phylogenetic and phylogeographic inferences in angiosperms: the tortoise and the hare IV. Am J Bot. 2014;101(11):1987-2004. doi: 10.3732/ajb.1400398.

78. Dong WP, Xu C, Li CH, Sun JH, Zuo YJ, Shi S, et al. *ycf1*, the most promising plastid DNA barcode of land plants. Sci Rep. 2015;5(1):1-5. doi: 10.1038/srep08348.

79. Amar MH. *ycf1-ndhF* genes, the most promising plastid genomic barcode, sheds light on phylogeny at low taxonomic levels in *Prunus persica*. J Genet Eng Biotechnol. 2020;18(42):1-10. doi: 10.1186/s43141-020-00057-3.

80. Pederneiras LC, Gaglioti AL, Romaniuc-Neto S, Mansano VF. The role of biogeographical barriers and bridges in determining divergent lineages in *Ficus* (Moraceae). Bot J Linn Soc.

2018;187(4):594-613. doi: 10.1093/botlinnean/boy036.

81. Wei ZD, Kobmoo N, Cruaud A, Kjellberg F. Genetic structure and hybridization in the species group of *Ficus auriculata*: can closely related sympatric *Ficus* species retain their genetic identity while sharing pollinators? Mol Ecol. 2014;23(14):3538-50. doi: 10.1111/mec.12825.

82. Zhang LF, Zhang Z, Wang XM, Gao HY, Tian HZ, Li HQ. Molecular phylogeny of the *Ficus auriculata* complex (Moraceae). Phytotaxa. 2018;362(1):039-54. doi: 10.11646/phytotaxa.362.1.3.

83. Zhang Z, Wang XM, Liao S, Tian HZ, Li HQ. Taxonomic treatment of the *Ficus auriculata* complex (Moraceae) and typification of some related names. Phytotaxa. 2019;399(3):203-8. doi: 10.11646/phytotaxa.399.3.4.

84. Wang J, Li Y, Li C, Yan C, Zhao X, Yuan C, et al. Twelve complete chloroplast genomes of wild peanuts: great genetic resources and a better understanding of *Arachis* phylogeny. BMC Plant Biol. 2019;19(1):1-18. doi: 10.1186/s12870-019-2121-3.

85. Jiao YJ, Feng GY, Huang LK, Nie G, Li Z, Peng Y, et al. Complete Chloroplast Genomes of 14 Subspecies of *D. glomerata*: Phylogenetic and Comparative Genomic Analyses. Genes. 2022;13(9):1621. doi: 10.3390/genes13091621.

Supporting information

S1 Table. The collecting information of samples in the study. (xlsx)

S2 Table. Detailed information for the high-throughput sequencing data in the study. (xlsx)

Comments of Editor:

Thank you for submitting your manuscript to PLOS ONE. After careful consideration, we feel that that the revision was sufficient regarding the content, but the language still needs to be improved. Specifically the newly inserted text has flaws in grammar and wording. Please ask a native English speaker for help or use a professional editing service.

Answer: Done. We sought a professional editing service to improve our language. All the changes were tracked, so another clean version was provided meanwhile. We hope that the revised manuscript could meet the language requirement of PLOS ONE.
