# Supplementary material for: Comparison of chloroplast genomes and phylogenomics in the Ficus sarmentosa complex (Moraceae)
Source: PLoS One. 2022 Dec 30;17(12):e0279849. doi: 10.1371/journal.pone.0279849 (PMC9803296; doi:10.1371/journal.pone.0279849)
Supplement: Attachment — Submitted filename: Response to academic editor and reviewers.docx [file pone.0279849.s003.docx]

**Response to academic editor:**

1. Please ensure that your manuscript meets PLOS ONE's style requirements, including those for file naming. The PLOS ONE style templates can be found at https://journals.plos.org/plosone/s/file?id=wjVg/PLOSOne_formatting_sample_main_body.pdf and <https://journals.plos.org/plosone/s/file?id=ba62/PLOSOne_formatting_sample_title_authors_affiliations.pdf>

Answer: Done.

2. Please include your tables as part of your main manuscript and remove the individual files. Please note that supplementary tables (should remain/ be uploaded) as separate "supporting information" files.

Answer: Done.

3. Thank you for stating the following financial disclosure: “This research was funded by National Natural Science Foundation of China from Dr. De-Shun Zhang, grant number 32071824. The funder De-Shun Zhang implemented the conceptualization and visualization of the study.” Please state what role the funders took in the study. If the funders had no role, please state: "The funders had no role in study design, data collection and analysis, decision to publish, or preparation of the manuscript." If this statement is not correct you must amend it as needed. Please include this amended Role of Funder statement in your cover letter; we will change the online submission form on your behalf.

Answer: Done.

4. Thank you for stating the following in your Competing Interests section: “NO authors have competing interests” Please complete your Competing Interests on the online submission form to state any Competing Interests. If you have no competing interests, please state "The authors have declared that no competing interests exist.", as detailed online in our guide for authors at <http://journals.plos.org/plosone/s/submit-now>  This information should be included in your cover letter; we will change the online submission form on your behalf.

Answer: Done.

5. We note that you have stated that you will provide repository information for your data at acceptance. Should your manuscript be accepted for publication, we will hold it until you provide the relevant accession numbers or DOIs necessary to access your data. If you wish to make changes to your Data Availability statement, please describe these changes in your cover letter and we will update your Data Availability statement to reflect the information you provide.

Answer: Done. We provided the accession numbers for the sequencing data and assembled genomes. Besides, we stored our raw sequencing data in Genome Sequence Archive BIG Data Center (<http://bigd.big.ac.cn/gsa>). If this database is not permitted for our Journal, we will reload to NCBI instead.

**Response to Reviewer 1.**

Reviewer #1: The manuscript entitled "The comparison of chloroplast genomes and phylogenomics at closely related species: a case of Ficus sarmentosa complex (Moraceae)" by Zhang et al. tried to estimate phylogenetic relationships of a species complex of the genus Ficus using newly obtained 13 chloroplast sequences mined from whole genome sequencing reads. The manuscript are well-written, and I think it seems be satisfying most of publication criteria of this journal but I have some concerns as follows.

Major comments:

1. The taxonomic groups used by the authors will be not used by some other authors as in table 1 of Rasplus et al. (2021), such as the subsection class. So it will be better to provide the information on which classification followed by the authors as the table 1 of Rasplus et al. (2021).

Rasplus, J. Y., Rodriguez, L. J., Sauné, L., Peng, Y. Q., Bain, A., Kjellberg, F., ... & Cruaud, A. (2021). Exploring systematic biases, rooting methods and morphological evidence to unravel the evolutionary history of the genus Ficus (Moraceae). Cladistics, 37(4), 402-422.

Answer: Done. We checked the full text and added the taxonomical classification of the names at the subsection level. Please see Line 68 in the manuscript of clean version rather than version under track changes (similarly hereinafter).

2. Although the authors did not mention, all subgenera seems paraphyletic in the phylogeny. The authors should discuss this result itself and the utility of chloroplast genome to estimate phylogeny of the genus Ficus. And lack of the section names in each clade of fig 8, some sentences especially results of phylogenetic analysis are difficult to follow.

Answer: Yes, all six subgenera are paraphyletic. In the revised manuscript, we redid the phylogenomic analysis based on the new dataset, only *Ficus* and outgroup trib. Olmedieae. So we rewrote the results and discussion sections. Figure 8 was also redone and the section names were added. Please see the results and discussion sections as well as Figure 8.

Minor comments:

Line 2 and elsewhere: Ficus sarmentosa complex -> the Ficus sarmentosa complex

Answer: Done.

Lines 15-16: The authors seem used more numbers of sequences in the phylogenetic analysis than two, please rephrase to make clear how many sequences obtained from the previous studies and which analysis used which sequences.

Answer: Done. The samples in the comparative genomics and phylogenomics are different. We rewrote the words to avoid ambiguity. Please see Lines 19-20 and 31-32.

Lines 42-44: "the latest research": the following research seems more recent than that the authors cite, please refer to this research also, if it related to this sentences.

Rasplus, J. Y., Rodriguez, L. J., Sauné, L., Peng, Y. Q., Bain, A., Kjellberg, F., ... & Cruaud, A. (2021). Exploring systematic biases, rooting methods and morphological evidence to unravel the evolutionary history of the genus Ficus (Moraceae). Cladistics, 37(4), 402-422.

Answer: Done. We supplemented some genomic references. Please see Line 46.

Lines 56 and elsewhere: "What’s more", although I'm not native speaker of English, but this phrase may seem too colloquial.

Answer: Done. We rewrote them or replaced this phrase with more normal ones. Please see Lines 59, 284, and 367.

Line 61: "complete genome": please make clear which of complete nuclear genome or chloroplast genome is meant by this phrase.

Answer: Done. Please see Line 66.

Lines 62-63: Please cite the reference for Plagiostigma because some authors do not use this taxonomic grouping, such as Cruaud et al. 2012.

Cruaud, A., Rønsted, N., Chantarasuwan, B., Chou, L. S., Clement, W. L., Couloux, A., ... & Savolainen, V. (2012). An extreme case of plant–insect codiversification: figs and fig-pollinating wasps. Systematic Biology, 61(6), 1029-1047.

Answer: Done. Please see Line 70.

Lines 106-107: "IR, LSC, SSC": Please spell out these abbreviations in the first use.

Answer: Done. We defined the abbreviations in Lines 110-111.

Lines 142-144: Please provide the accession numbers of these 126 sequences such as in the supplemental table.

Answer: Done. We updated the dataset and reconstructed the phylogenomic tree, and the accession numbers were added in S1 Table.

Lines 142-144: "these genomes are unavailable in GenBank currently)": if so, how did the authors obtained those sequences?

Answer: The authors (Bruun-Lund et al.) provided the genomic sequences as the alignment nexus file in the Dryad website, rather than in GenBank. We rewrote the sentence, and please see Lines 152-153.

Line 144: "138": this may be typo but 126 and 13 sequences will be 139. Please explain what was happen if the number of sequences truly is 138.

Answer: 138 is right, and 126 should be 125 because one more sample was counted. However, we redid the phylogenomics based on the new dataset, and the number of samples is 129 now. Please see Line 159.

Lines 151: Not only the reads of chloroplast but also the basic information on the raw reads containing nuclear reads, such as total reads and percentage of chloroplast reads in the total reads will be informative.

Answer: Done, we added the statement about these related resequencing data. Please see Lines 170-175 and S2 Table.

Lines 170-171: Please add the citation for the statement, "infA gene is lost in Ficus".

Answer: Done. Please see Line 193.

Lines 254-274: Although the authors did not mention, all subgenera seems paraphyletic in the phylogeny. Please see the major comment.

Answer: Done. We rewrote the result and discussion sections about phylogeny. Please see Lines 332-333.

Line 258 and elsewhere: Ficus -> the genus Ficus

Answer: Done.

Lines 260 and 261: "basal group": it is better to avoid using this phrase according to the following article.

Krell, F. T., & Cranston, P. S. (2004). Which side of the tree is more basal?. Systematic Entomology, 29(3), 279-281.

Answer: Done. We rewrote the sentences. Please see Lines 322-327.

Fig. 8: Please add the section name to each clade, some sentences especially results of phylogenetic analysis are difficult to follow because of lack of this information. Please see the major comment.

Answer: Yes, all six subgenera are paraphyletic. In the revised manuscript, we redid the phylogenomic analysis based on the new dataset, only *Ficus* and outgroup trib. Olmedieae. So, we rewrote the results and discussion sections. Figure 8 was also redone and the section names were added. Please see the results and discussion sections as well as Figure 8.

Table 1: If these accession numbers are only for the chloroplast sequence, please provide the accession numbers for the raw reads from NGS, too.

Answer: Done. The accession numbers and sequencing information were shown in S2 Table.

**Response to Reviewer 2.**

Reviewer #2:The title and objective do not match the described study with respect to the sampling and discussion. The authors should revisit their objectives or sampling to ensure the manuscript is well-aligned from introducing the background and scope to the chosen sampling and the presentation and discussion of the results.

While the 11 new chloroplast genomes are a valuable contribution and a lot of detailed analysis of the genomes are provided and is clearly the authors strength, unfortunately with the chosen sampling strategy, the study adds very little to its objective of understanding the usefulness of chloroplast genomes to resolve species complexes or the taxonomic problems of the targeted species complex.

Answer: Thanks very much for your worthwhile suggestions. In the revised manuscript, we focused on the comparative chloroplast genomics and phylogenetic potential in the *F. sarmentosa* in the study, rather than trying to solve the taxonomical puzzle based on our limited samples in the *F. sarmentosa* complex. On the basis of the high-resolution phylogenetic relationship in the *F. sarmentosa* complex and another well-sampled species complex (*F. auriculata* complex)(Fig 8), chloroplast genomes showed a promising resolution in the closely related species. For these aims, we rewrote the title and the corresponding result and discussion sections. We hope the revised manuscript has a distinct improvement in the matching between the title and the sampling and discussion.

Introduction

l.52. The authors state that only a few chloroplast genomes have been reported in Ficus, but in the next line refer to a study by Bruun-Lund et al. (2017) including 59 species of Ficus, which I would not call only a few genomes.

Answer: What we intend to mean is that *Ficus* genomes in the public data repository GenBank are relatively limited (less than five percent). We have rewritten the words to avoid ambiguity. Please see Lines 55-57 in the manuscript of clean version rather than version under track changes (similarly hereinafter).

The authors of the submitted manuscript rightly explain that the results from Bruun-Lund showed conflicts between the new plastome topology and previous nuclear studies for both individual species as well as relationships among some sections at deeper levels.

Answer: Indeed, the inconsistency between the plastome and nuclear genome is common in *Ficus* and other taxa.

l. 56. However, the authors of the present manuscript appear to ignore this issue arguing "that the majority of genome sequences of Bruun-Lund et al. were relatively incomplete and perhaps disturbed the accuracy of phylogenetic tree due to an abundant of missing data". However, it is not explained what relatively incomplete is and no examples or argumentation is provided for this potentially being an issue.

Answer: Based on the work of Roure et al., abundant missing data will negatively affect the precision of the phylogenetic tree, while slight missing data (such as less than 20%) might not matter. Besides, the incomplete genomes are different to utilize in the comparative chloroplast genomics. Based on these points, we rewrote the sentences. Please see Lines 55-59.

*Roure, B.; Baurain, D.; Philippe, H. Impact of missing data on phylogenies inferred from empirical phylogenomic data sets. Molecular Biology and Evolution* ***2013****, 30, 197-214. doi:10.1093/molbev/mss208.*

l. 61. The authors state that "The species of subsect. Plagiostigma is under taxonomically unresolved and genetically unclear, formed a species complex and an ideal candidate for the researches of hybridization, radiation speciation, biogeography, and interaction between plant and pollinator". In addition to a need for revisiting this sentence for unclear English and meaning, the authors should provide a short discussion of the taxonomic history of the F. sarmentosa complex if this is the primary focus of the manuscript. What was learned from the study by Zhang et al. (reference 3, a well-written manuscript by several of the same authors) compared to Berg and Corner (2005).

Answer: Our study doesn’t seek to deal with the taxonomical puzzles in the *F. sarmentosa* complex. The comparative chloroplast genomics and the examination of the phylogenetic potential of chloroplast genomes in closely related species.

Even so, we also tried to rewrite the part to improve our language and provide a brief statement on the taxonomical history of the complex based on your advice. Please see Lines 72-82.

l. 65. The F. sarmentosa complex or subsect. Plagiostima sensu Berg and Corner (2005) includes at least 10 species and is part of larger taxonomic problem which at the moment includes all the root climbers of subg. Synoecia and it would have been logical and much more interesting to expand on the sampling of this entire subgenus.

If the aim is indeed to resolve the F. sarmentosa complex and discuss the usefulness of chloroplast genomes to resolve closely related species, it would be more appropriate to expand the sampling of this complex with several samples per taxon.

It is also not clear why sampling extends to 61 additional chloroplast genomes of Moraceae. It would appear to be enough to include the Olmedieae based on previous studies.

Answer: The *F. sarmentosa* complex indeed is a complicated taxonomical issue, while it should be a separate lineage in the subg. *Synoecia*. Based on our published work (Zhang et al. 2020) or some unpublished data, the complex is far away from the rest of subg. *Synoecia*, though the latter also includes some other unsolved groups, such as *F. villosa* group, *F. disticha* group, and *F. punctata* group.

In the revised manuscript, the aim is limited to comparative genomics and the exploration of the resolution of the chloroplast genome in related species, rather than to resolve the taxonomy in the *F. sarmentosa* complex. Multiple samples per taxon in the complex or sampling for the entire subgenus should be unnecessary and digressed under the current study aim. We think the support values (bootstrap values) of nodes within the complex (Figure 8) can be useful to access the phylogenetic performance of plastid genomes in closely related species.

For another, we agree with the suggestion about the extent of sampling, so eight samples were added for the complex. The samples beyond *Ficus* and Olmedieae in Moraceae were discarded.

l. 77. Following the objectives, the authors state "we sampled the plastomes of eleven taxa and then finished the comparative genomics and phylogenomic analysis of F. sarmentosa complex", which does not adequately reflect the results obtainable with this sampling or the discussion of these results.

Answer: Done. We rewrote the sentence. Please see Lines 82-85.

Materials and methods.

l. 82 onwards. Here you should refer back to the introduction outline of the F. sarmentosa complex and explain your sampling strategy and how well it represents the diversity of your study group.

Answer: Done. Please see Lines 90-91.

l. 143. It is stated that: "Thirteen chloroplast genomes, additional 61 available Moraceae chloroplast genomes in GenBank and 65 genomes from the work of Bruun-Lund et al [22] (these genomes are unavailable in GenBank 144 currently) were chosen". Do you mean these genomes are available? It is not clear since they were obviously included in your study.

Answer: The authors (Bruun-Lund et al.) provided the chloroplast genomic sequences as an alignment nexus file on the Dryad website, rather than in GenBank. We rewrote the words for a clearer statement on the sampling. Please see in Lines 152-157.

Results.

Considering your hypothesis is the introduction "that the majority of genome sequences of Bruun-Lund et al. were relatively incomplete and perhaps disturbed the accuracy of phylogenetic tree due to an abundant of missing data", you need to provide a detailed comparison of whether and how your results have indeed improved on completeness and whether this has significantly improved relationships.

This would also provide the necessary background for the discussion l. 332 stating "However, in our focused group Ficus, 35 newly added genomes didn’t affect the linage relationship compared to the previous study of Bruun-lund et al."

l. 259. How does your topology compare to the one published by Bruun-Lund et al. and the taxonomic framework provided by Clement et al. (2020)?

Answer: The comparison between our phylogenetic result and previous research (such as Bruun-Lund et al. and Clement et al.) was finished in the Discussion section. Please see Lines 408-422.

l. 268. "For the 13 taxa in F. sarmentosa complex, two obvious linages were recognized and the most of nodes within these two linages were well-supported (Figure 8)". Please discuss how the findings match previous taxonomic understanding of the complex and in particular clarify that two of the F. sarmentosa samples result in polyphyly of Synoecia.

Answer: Based on our new extended dataset, the phylogenetic relationship of the *F. sarmentosa* complex has a great change. Thus, we rewrote the results section. The discussion about how our tree contributed to the taxonomy of the *F. sarmentosa* complex and the cladistics of *Ficus* was finished in the Discussion section. Please see Lines 423-432.

Discussion.

L. 278-300. If it has been widely demonstrated that the chloroplast genome is relatively conserved and provide little information among closely related species, it is not clear why the authors would focus on exploring this in close relatives or a megadiverse genus like Ficus.

Answer: Though the structure conservation is well acknowledged in the chloroplast genomes, the detailed variation pattern and evolutionary hotspot in the context of recent speciation (such as species complex) know little. The deep comparisons of these closely related species can improve our insight into mechanisms, rates, or directionality of genome evolution, etc.

Simultaneously, we rewrote the words for a clear statement of our study aims. Please see Lines 355-363.

l. 328. The authors state that "The ML phylogenetic tree is generally consistent to previous researches based on hundreds of nuclear genes [63], such as Morus and Streblus are reconfirmed as non-monophyletic groups. The consistent results revealed the great potential of chloroplast genome in phylogenomics of Moraceae". It is not clear what the point is and why re-analysing previously published data would not be expected to result in more or less the same topology for Moraceae. If you want to discuss the great potential of chloroplast genome in phylogenomics of Moraceae, you could focus on discussing stability of the results among previous studies of which there are quite a few.

Answer: As our study has shown, our ML tree of Moraceae is not a re-analysis, because our tree is reconstructed based on as many as possible chloroplast genomes but previous studies often are based on the nuclear loci. However, we redid the tree based on the new dataset (only *Ficus* and tribe Olmedieae) in the revised manuscript, so these sentences were removed.

l. 340. Please revisit the manuscript including the discussion for sentence clarity possibly with the help of a professional English speaker or consultant. See for example the sentences: "Whereas, current phylogenetic frameworks of Ficus were acquired by only up to six nuclear loci [12, 64] or limited and disproportional samples [14]. So, it is still deficient to distinguish the origin of the cyto-nuclear conflict accurately, such as gene flow (hybridization or introgression) or incomplete linage sorting".

Answer: Done. Based on the helpful comments from the reviewer and a professional English speaker, we tried to improve our text, and we hope it will be an obvious improvement.

l.343. Again, it should be discussed what your results learns us about the F. sarmentosa complex.

Answer: Done. Considering the taxonomical issue of the *F. sarmentosa* complex is not our key aim, we only briefly discussed their phylogenetic relationship. Please see Lines 423-432

L. 351. Several times it is mentioned that the chloroplast genome helps to identify potential cyto-nuclear discordance, but it is not really discussed how this could be further explored and verified.

Answer: Again, considering the phylogeny of the complex is not our aim, we rewrote the words without discussing it. Please see in Lines 420-422 and 439-441.

l. 365. The conclusion: "Phylogenomic construction indicated that chloroplast genomes show a promising resolution for phylogenetic relationship of closely related group with the evident cyto-nuclear discordance" is not well-explained and is an over-statement and contra=diction of what is actually analysed and the results obtained in this study.

Answer: We added more samples of the *F. sarmentosa* complex and another species complex *F. auriculata* complex, and then reanalysis the phylogeny. Based on our phylogenetic results and other related studies on the closely related species, we think that we have a reasoned argument for the promising resolution. Of course, we revised the corresponding parts in the revised manuscript. Please see Results and Discussion sections.
