# Supplementary material for: Comparison of chloroplast genomes and phylogenomics in the Ficus sarmentosa complex (Moraceae)
Source: PLoS One. 2022 Dec 30;17(12):e0279849. doi: 10.1371/journal.pone.0279849 (PMC9803296; doi:10.1371/journal.pone.0279849)
Supplement: Attachment — Submitted filename: Response to Reviewers.docx [file pone.0279849.s004.docx]

Comments of Editor:

Thank you for submitting your manuscript to PLOS ONE. After careful consideration, we feel that that the revision was sufficient regarding the content, but the language still needs to be improved. Specifically the newly inserted text has flaws in grammar and wording. Please ask a native English speaker for help or use a professional editing service.

**Answer: Done. We sought a professional editing service to improve our language. All the changes were tracked, so another clean version was provided meanwhile. We hope that the revised manuscript could meet the language requirement of PLOS ONE.**
