# Supplementary material for: Comparison of chloroplast genomes and phylogenomics in the Ficus sarmentosa complex (Moraceae)
Source: PLoS One. 2022 Dec 30;17(12):e0279849. doi: 10.1371/journal.pone.0279849 (PMC9803296; doi:10.1371/journal.pone.0279849)
Supplement: Attachment — Submitted filename: Response to Comments.docx [file pone.0279849.s006.docx]

The language mistakes pointed out by the editor have been simply accepted, so only one key comment was detailedly responded to here.

**Response to comments of the editor:**

For example, a study of 22 closely related *Oryza* species indicated that conservation was common at lower taxonomic levels [29]. A study of four peanut varieties serves as a more extreme example, reporting perfectly identical IR boundary junction positions [69]. Although comparative chloroplast genomic studies have rarely focused on closely related species, the high conservation of chloroplast genome among closely related species is recognized based on our work and other related research.

Answer: Done, the study is very relevant, and we have added it. Please see Lines 376-377. The serial numbers of the reference were changed, too.

**A list of corrections beyond the comments of the editor:**

Line 38: widely distributing across tropical and subtropical regions [1-3]. Due to insufficient genetic

Correction: …distributed…

Line 53: ORG.asm (https://git.metabarcoding.org/org-asm/org-asm), plastome-based evolutionary research have become easier and more cost-effective [22-24].

Correction: …has…

Line 57: results of which were obvious inconsistent with phylogenies based on the nuclear genome.

Correction: …obviously…

Line 60-61: Even so, the chloroplast framework of Brunn-Lund et al. have been replicated by subsequent research generally with an extended dataset [17].

Correction: …has…

Line 73: several controversial taxa in 1980s [1, 34-37] (such as *F. dinganensis*, *F. guizhouensis*, and *F.*

Correction: …in the 1980s…

Line 78: relationship between the complex and these previously described species in 1980s. However, because

Correction: …in the 1980s…

Line 156: samples from the Olmedieae tribe were chosen as the outgroup, according to the previous studies [26, 52].

Correction: …according to previous…

Line 183: *sarmentosa* var. *nipponica* and *F. sarmentosa* var. *thunbergii*). The CDS regions ranged from 79,149

Correction: Delete one extra space.

Line 234: 60 bp in length was the least, appearing in only once. The maximum length among all the long repeat

Correction: …were…appearing only…

Line255: times. The marjority of the remainding motifs appeared only once to four times, with the exception of

Correction: …majority…remaining…

Line 290-291: exhibited the highest nucleotide diversity: 0.00535, 0.00462, 0.00456, 0.00427 and 0.00377, respectively (Fig 6).

Correction: …0.00427, and 0.00377…

Line 311: signals of positive selection. An infinite Ka/Ks ratios (Ka > 0 and Ks = 0) existed in 27 genes (Fig 7).

Correction: …ratio…

Line364: genomes from the *F. sarmentosa* complex*.* Overall, the length of both whole-genome and quadripartite

Correction: …lengths…

Line 404: The plastomic phylogeny of the genus *Ficus* and the phylogenetic

Done. …plastome…

Line 410: mysterious displacment of certain individuals (*F. fulva*, *F. magnoliifolia*, *F. albipila*, *F. albert-smithii*, and *F. pumila*).

Correction: …displacement…

Line 438: rice [29], willow [68], and Orchardgrass [86]. Taken together, the chloroplast genome appears

Correction: …orchardgrass…
